# Supplementary material for: Using nasal sprays to prevent respiratory tract infections: a qualitative study of online consumer reviews and primary care patient interviews
Source: BMJ Open. 2022 Jun 30;12(6):e059661. doi: 10.1136/bmjopen-2021-059661 (PMC9247325; doi:10.1136/bmjopen-2021-059661)
Supplement: Supplementary data [file bmjopen-2021-059661supp001.pdf]

Supplementary material 1: RECUR ("Immune Defence") Guiding principles – Nasal sprays

User context	Key design objective	Intervention design features
Our users have a range of nasal spray experience. Some have used sprays before, such as for hayfever, but perhaps not using the techniques we recommend for this spray. Some have never used a nasal spray before.<br><br>The idea of using sprays preventatively is a novel idea for most target users.	To support new and experienced nasal spray users to build skills to develop a correct spray technique.	• Provide an instructional video on a 'live model' demonstrating correct spray technique and modelling behaviour to build skills for new users.<br>• Provide an instructional booklet to be kept with the spray for ease of use and a reminder of how to use the spray correctly.<br>• Highlight specific advice which will be helpful for new and experienced sprays users (e.g. do not inhale deeply when the spray is in your nose).<br>• Advise everyone to watch the video including experienced sprays users, highlighting that this spray is administered in a different way.
Our target group perceive the spray as exciting and they are hopeful it will work. However, they may not always experience benefits when they begin using it and this could lead to disappointment and (depending on other factors, including side effects) discontinuation or suboptimal adherence. Some users also have previous experience of trying other prevention strategies with little success, leading to a fatalistic perspective of RTIs.	To support people to set and maintain positive expectations of spray efficacy, even when RTIs still occur.	• Explain the mechanism of how the spray works so that people understand that the aim of the spray is to reduce viral load not prevent viruses entering the body at all.<br>• Emphasise how sprays reduce the duration and severity of an RTI, as well as preventing them in the first place, to avoid feelings of disappointment if people do get an RTI after using the spray.<br>• Address the potential lack of efficacy beliefs by providing advice for future use (e.g. using the spray quickly at first signs of infection, using the correct technique) and motivating the user to keep trying the spray, even if they got an infection.

Concerns about nasal spray side effects are fairly common and may lead to discontinuation of use.	To reduce people's concerns about possible negative effects of spray use.	• Provide reassuring information to address concerns about potential side effects, specifically regarding the mildness and tolerability of the spray.<br>• Change perceptions of side effects – framing them positivity as 'normal' by describing what to expect. Explaining side effect symptoms as a sign that the spray is working effectively (e.g. getting to the areas it needs to in order to work).<br>• Compare side effects to worse outcomes if nasal spray was not used e.g. getting an infection.<br>• Provide advice and skills training on how to optimise your nasal spray technique to avoid side effects.<br>• Advise on how to cope with side effects if they did happen e.g. how to deal with a nosebleed.
People may see nasal sprays as medicines because of their mode of administration and previous experiences with sprays that are medicines (e.g. hayfever, sinus). Many people have concerns about over-use of medicines.	To help develop an alternative way of thinking about the spray, to reassure people about safety and to persuade people that nasal sprays are safe.	• Explain that nasal sprays work in a similar way to handwashing/hand gel. This provides a familiar example of something that is not a medicine but helps prevent infections. Both prevention methods are common behaviours, simple and acceptable, and they neutralise/remove germs/viruses before they can infect you and make you ill.<br>• Address the concern that the spray is a medicine by clarifying that it is not a medicine and that it is safe and non-addictive.

The intervention will be delivered during the COVID-19 pandemic where the threat levels and national recommendations are constantly changing.<br><br>Emerging evidence suggests that nasal sprays may be useful in providing additional protection against viruses like COVID-19, which act similarly to common RTIs but have severe consequences, particularly for vulnerable groups.<br><br>Our target group clearly spot the similarities between COVID and seasonal/normal RTIs. Those using the intervention in a pandemic context may be concerned with either or both COVID/normal RTIs.<br><br>Some of our target group have incorrect beliefs that the spray can replace government COVID recommendations or that the spray is not needed because of other behaviours mitigating the usefulness (e.g. mask wearing, social distancing).	To ensure the intervention is suitable for delivery during rapidly changing COVID-19 pandemic context.	• Be able to quickly update intervention content when needed to reflect latest guidelines and research evidence.<br>• Explain how effective the spray might be for COVID-19.<br>• Correct misconceptions about nasal sprays and COVID-19 by explaining that the spray is another layer of protection to be used with other behaviours to ensure the best protection possible again infections.
