# Supplementary figures and images for: Using nasal sprays to prevent respiratory tract infections: a qualitative study of online consumer reviews and primary care patient interviews
Source: BMJ Open. 2022 Jun 30;12(6):e059661. doi: 10.1136/bmjopen-2021-059661 (PMC9247325; doi:10.1136/bmjopen-2021-059661)

Supplementary material 2: Logic model

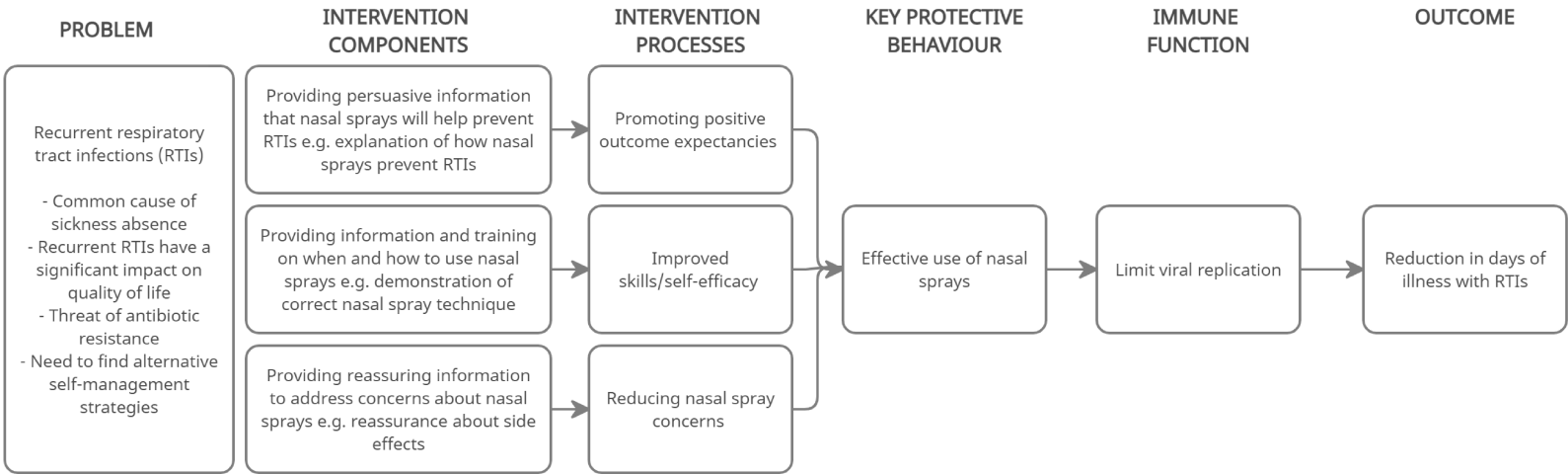

Supplement: Supplementary data [file bmjopen-2021-059661supp002.pdf]
