# Supplementary material for: Using nasal sprays to prevent respiratory tract infections: a qualitative study of online consumer reviews and primary care patient interviews
Source: BMJ Open. 2022 Jun 30;12(6):e059661. doi: 10.1136/bmjopen-2021-059661 (PMC9247325; doi:10.1136/bmjopen-2021-059661)
Supplement: Supplementary data [file bmjopen-2021-059661supp003.pdf]

Supplementary material 3: Table of Changes extract

This is a simplified extract from a "table of changes" used to optimise the Immune Defence nasal spray intervention. It has been edited for clarity for readers outside of research team.

Website Section/content referred to	Original website content wording	Participant comments: positive, or likely to promote engagement/adherence	Participant comments: negative, or likely to impair engagement/adherence	Action/Changes	Final website content wording
"When do I use the spray?"<br><br>Description of first signs of an infection that should trigger use of the spray.	There are 3 ways to use the spray.<br><br>**1.When you first feel an infection coming on.**<br><br>It works best if you use it **as soon as you notice any symptoms.**<br><br>Often people say their first signs are:<br>• Sneezing;<br>• A runny nose;<br>• A tickle in the nose; or<br>• A tickle in the back of the throat	**INTERVIEWER:** So what would you say are your kind of first signs of infection? **PARTICIPANT 1:** Mine are usually sneezing and a tickle. **INTERVIEWER** Yes, so you'd be able to identify yourself in those symptoms? **PARTICIPANT 1:** Oh yes, yes.<br><br>**PARTICIPANT 3:** So I suppose when I first feel an infection coming on is when the sore throat starts. **INTERVIEWER:** So that would be your first sign of an infection happening? **PARTICIPANT 3:** That would be the warning sign, definitely.<br><br>**PARTICIPANT 4:** [participant reads website] "*Often people say the first signs are sneezing*", yes, agree with that; "*runny nose*", yes; "*tickle in the back of the nose or a tickle in the back of the throat*", yes. The other thing is a headache or feeling hot and cold - feeling hot is another sign for me anyway.<br><br>**PARTICIPANT 3**: It's great. It's absolutely everything that I and my family feel and experience.	**PARTICIPANT 7:** I think that makes sense, and you haven't said, 'These are necessarily the signs that you would get when you feel an infection coming on.' You say, 'That's what often people say they are,' so... **INTERVIEWER**: So are your first signs recognisable in there, or not? **PARTICIPANT 7:** No. I don't know. I think I tend to just feel more rundown, tired, a bit headachy. I don't know. I wouldn't say I get a runny nose at all. No, I wouldn't say they are, to be honest!<br><br>**PARTICIPANT 1:** I can't think of any other additional things that would indicate that I had a nasal infection coming on. I would perhaps personally, sometimes I get a thick throat, like the equivalent of catarrh building up… but whether that comes under a tickle, I don't know, but that's what I personally would get as an indication, like just a thickening of the mucus<br><br>**INTERVIEWER**: Would you say those first signs of infection are similar to what you experience, or is it different? **PARTICIPANT 10**: Yes.	We added a catch-all statement about a wide range of early signs of RTIs, acknowledging idiosyncrasies and building confidence in spotting own first signs.	There are 3 ways to use the spray.<br><br>**1.When you first feel an infection coming on.**<br><br>It works best if you use it **as soon as you notice any symptoms.**<br><br>Everyone's first signs of infection are different. Often people say their first signs are:<br>• Sneezing;<br>• A runny nose;<br>• A tickle in the nose;<br>• A tickle in the back of the throat;<br>• Your skin feeling sensitive;

			I think the first signs, they are but the first signs are often an aching, aren't they and sore? I get sore skin and aching li-, just a general ache as a first sign quite often of these bugs. So sometimes they are, but I suppose that might be the difference between colds and flu, I don't know. Sore throat I'd say, rather than a tickle in the back of the throat, but you say often…I'm not sure about these symptoms. Maybe sometimes, but not always. I think for me I often feel achy and have this very funny sensitive skin which seems a bit sore all the time, that would be the first sign, but maybe as I say, that's the difference between flu and cold, I don't know. If that's what people say then that's what most people say then that's a thing, isn't it?		• Having slightly achy muscles; or<br>• Having a mild headache.
"How does the spray work?"<br><br>Comparison of using the nasal spray to washing.<br><br>In various places the content refers to a washing/washing out metaphor and a comparison to washing hands with soap and	It's a bit like using soap when you wash your hands, only better.<br><br>The spray traps viruses and washes them out of the nose; and make the nose and throat **a very unfriendly place for viruses.**<br><br>This means it's **much harder for them to survive**	**PARTICIPANT 4** [Reading website] How does it work? *"It's a bit like using soap and wash your hands only better!*" Oh, I like that.<br><br>**INTERVIEWER:** What sounds particularly appealing about giving it a go? **PARTICIPANT 5**: It says that, well, the whole bit of how does it work? *"The spray traps the viruses and washes them out of your nose. Makes your nose and throat a very unfriendly place for the viruses. This means it's much harder for them to survive"*. I'd be willing to give that a go!	**PARTICIPANT 4**: The spray traps the viruses and washes them out of the nose. Ooh, how does it wash it out of the nose? You spray it up and then it all runs down. That sounds disgusting. Do you spray it and then blow out? **INTERVIEWER**: You've got a question there about how to use it essentially, is that right? **PARTICIPANT 4**: It says here just - well, I know you know what it says but, *'Spray traps the viruses and washes them out of the nose.*' All right. Let's read the next sentence. "*Makes the nose and throat a very unfriendly place for viruses. This means it's much harder for them to*	We retained the hand cleaning metaphor but change to a comparison with hand gel rather than soap and water to provide a closer match to the spray action and avoid the procedure sounding difficulty or unpleasant. Hand gel use is currently (early	It's a bit like a hand gel, but specially designed for your nose.<br><br>The spray helps to clean the virus from your nose.<br><br>The spray also makes the nose and throat a very **unfriendly place for viruses.** This means it's much **harder for them survive** so they

water to remove germs before they can do harm.	so they can't take hold and make you ill.	**INTERVIEWER:** Does the explanation about how it works make sense to you, about the soap and hands? **PARTICIPANT 3:** Yes, indeed. Yes, I'll say!<br><br>**INTERVIEWER:** and the explanation saying 'it's a bit like using soap when you wash your hands', does that seem to make sense to you? **PARTICIPANT 1:** Well, it does, particularly in the current situation with coronavirus!<br><br>**INTERVIEWER:** in terms of that explanation about how it works, being like soap for washing your hands, does that make sense to you, that explanation? **PARTICIPANT 7:** Yes, it does. I'm wondering if soap changes your pH levels because I've never really thought about that! It makes sense though, yes, and you said it traps viruses and washes them out of the nose, so that makes sense, I think.	*survive so they can't take hold and make you ill".* Right. Well, I'd like to know this washing out of the nose... **INTERVIEWER** Yes, because your first reaction to that was that sounds a bit gross - almost a bit disgusting. **PARTICIPANT 4**: Yes! It's like do I spray the liquid up my nose and then it all runs down my face? Or are you meant to spray it up your nose; then sniff it up; and then you swallow it? Which sounds equally disgusting I might add. I don't like that... The spray traps viruses, absolutely happy with that but washes them out of the nose has all sorts of horrible connotations.<br><br>**INTERVIEWER** You were anticipating almost that it has to come out of the nose essentially whereas this is suggesting... **PARTICIPANT 4**: Yes, exactly. On that video, all you're doing is placing the liquid spray into the nose as the barrier to the virus but there's no washing out. It's like putting some deodorant on. You put it on, and you leave it in place because it's got a job to do.	COVID pandemic) a common anti-infection product people are using with confidence.	can't take hold and make you ill.
Video – instructions how to use the spray	Part of the video demonstrates high risk situations in which you should use the nasal spray. It depicts a person being sneezed on by	-	**INTERVIEWER**: I heard you had a little bit of a giggle at one point. **PARTICIPANT 3**: [Laughs] Yes, that was just the second person sneezed all over the girl who was doing the demonstration.	No change. Content is engaging, enjoyable (and the intended message was clearly understood).	-

	somebody else (acting).				
Video – instructions how to use the spray	Part of the video demonstrates exactly how to prime the spray, insert into nostrils, spray and sniff.	**PARTICIPANT 5:** Yes, no, I think it's good. It's very informative and it's good that it gives people, like it shows people how to correctly use the spray because sometimes, the instructions on the boxes and in the packets and things, they aren't always as clear. **INTERVIEWER**: Do you think you'd find that useful yourself if you were trying out the spray for the first time? **PARTICIPANT 5:** Yes, definitely.	**INTERVIEWER:** we do have a link there to a video. You don't need to click on that. I just wanted to ask you, with the information that's here would you need to click on that link to see the video, or do you think that you'd be okay with the instructions there? **PARTICIPANT 7:** I wouldn't because obviously I've used sprays before. Somebody that hasn't used, and is a bit wary of it, would probably click on it.<br><br>**INTERVIEWER** Would you be inclined to watch the video yourself if you wanted instructions, or would you be more…? **PARTICIPANT 1**: No, to be honest, it's so straightforward using a nasal spray… I wouldn't bother with the video… Particularly at my age range, you've probably used nasal sprays several if not many times over your lifetime so you just would just use it.<br><br>**INTERVIEWER** What did you think about that video? **PARTICIPANT 4**: Yes, it's common sense really. I've been using [another] spray for years... I do keep my head straight. I do do my one or two good puffs. I don't breathe out and spray it everywhere. It's common sense.<br><br>**PARTICIPANT 2** When I first got [the spray], I used it once, even though I didn't have any need to use it and	We attempted to get more people to watch the video, by emphasising how the way of using the spray might be different.<br><br>Given that we know that incorrect use/angle can increase likelihood of side effects, and that our instructions are different to other sprays (e.g. hay fever, sinus medications) it is vital people use it as per instructions rather than according to common sense.	Click **here** to see a short video to help you master the spray technique.<br><br>This video is worth watching even if you have used nasal sprays before. The technique for **this spra**y might be a bit different. Using the spray correctly give you the best chance of fighting infections!

			within seconds it had given me a headache, exactly as it did the very first time I used it about a year or so ago on the recommendation that was given to me. I felt, right, I'm not going to use it anymore. The only way I can describe the headache is it's like a freezer headache. It's exactly the same, if you take a bite of an iced lolly or something. That sort of, right between the eyes. I read about how it says that if that happens, you should aim it more towards your ear, rather than straight up…..[later in interview]……I**NTERVIEWER**: Why did you decide not to watch the video? **PARTICIPANT 2:**I think I was probably rushing off to do something, or I got distracted or, no, I didn't. I think probably arrogant, I probably thought, 'Oh, for goodness sake, I don't need to be shown how to use a nasal spray.' Although, clearly I did because once I used it as recommended, I didn't get a headache.		

Readers interested in using the Person-Based Approach to intervention development and who wish to use a Table of Changes to assist the process may find resources on this website helpful: https://www.lifeguideonline.org/pba
