# Supplementary material for: Using nasal sprays to prevent respiratory tract infections: a qualitative study of online consumer reviews and primary care patient interviews
Source: BMJ Open. 2022 Jun 30;12(6):e059661. doi: 10.1136/bmjopen-2021-059661 (PMC9247325; doi:10.1136/bmjopen-2021-059661)
Supplement: Supplementary data [file bmjopen-2021-059661supp004.pdf]

Supplementary material 4:

Study 2 - Interview Schedules for Phase A and Phase B

Phase A: Think-aloud interviews

Prompts about key pages of intervention content [e.g. menus/ first page etc.]

- What are your first impressions of this page?
- What are you thinking now?
- What made you choose that option?
- What do you think about [this activity, this information, this strategy/tool/idea]?
- Can you tell me a bit more about why you think that?
- [in response to an expression of like/dislike] What is it you like/don't like about that?
- That's really interesting…..
- [picking up on vocalisations/tone of voice etc] I noticed that you paused/groaned/laughed/sighed etc. at……… Can you tell me what you thought about that?

After working through the key pages of intervention content:

- Overall, what do you think about the web pages?
- Can you tell me about anything you thought was particularly good about the web pages?
- Can you tell me anything about the web pages that you were less keen on?
- Which parts did you find most relevant to you? Which parts were the least relevant to you?
- Having looked at the web pages, can you tell me how you feel about trying to use a nasal spray to try to reduce these sorts of infections
- How much of what you've seen today do you think is relevant to coronavirus?*
- How at risk do you feel about getting these infections at the moment?
- What do you feel about the recommendation to use the spray when at high risk and how this applies to coronavirus?*
- What device did you use to look at the website today?
    - If you were using the website over a longer period of time, how would you access the website?
    - Would you use mobile phone at all?

Phase B: Post-intervention interviews

- Can you start by telling me overall how you got on with trying the spray?

Questions if they have tried the spray:

- Can you tell me all about how you found using the nasal spray?
- Can you tell me about anything you liked or found easy?
- Can you tell me about anything you disliked or found difficult?
    - Can you let me know if there was anything you found helped with that?
    - Can you tell me about anything that worried you about using the spray?
- Can you tell me about when you used the nasal spray?
    - When did you think to use it?
    - Can you tell me about whether any situations came up where you could have used the spray (e.g. first symptoms, feeling a risk of catching an infection)? Can you tell about how you decided whether to use the spray?
- Can you tell me about what you thought were the advantages of using the nasal spray?
- Can you tell me about what you thought were the disadvantages of using the nasal spray?
- Can you tell me what it's been like for you trying these activities/changes whilst in (partial) lockdown because of coronavirus/COVID-19*?
    - Explore the context – have they been self-isolating? Shielding?
    - What have your infections been like during this time? (More/less?)
    - What aspects of lockdown have made it easier to try these activities/manage your infections?
    - What aspects of lockdown have made it harder to try these activities/manage your infections?
    - Can you tell me about any information or advice that was difficult for you to follow during lockdown?

Questions if they have not managed to try the spray:

- Can you tell about what you thought about the idea of using a spray to try to prevent infections?
- Can you tell me about anything about the spray that you liked or found easy?
- Can you tell about anything about the spray that seemed off-putting or difficult for you?
- Can you tell me about anything that worried you about using the spray?
- Can you tell me anything you feel would help you in the future with trying the spray?
- Can you tell me about whether any situations came up where you could have used the spray (e.g. first symptoms, feeling a risk of catching an infection)?
- Can you tell about how you decided whether to use the spray?
- Can you tell me about what you thought were the advantages of using the nasal spray?

- Can you tell me about what you thought were the disadvantages of using the nasal spray?
- Can you tell me what it's been like for you trying these activities/changes whilst in (partial) lockdown because of coronavirus/COVID-19?*
    - Explore the context – have they been self-isolating? Shielding?
    - What have your infections been like during this time? (More/less?)
    - What aspects of lockdown have made it easier to try these activities/manage your infections?
    - What aspects of lockdown have made it harder to try these activities/manage your infections?
    - Can you tell me about any information or advice that was difficult for you to follow during lockdown?

Website questions:

- What did you think of website that gave you information and advice about using the nasal spray?
- Can you tell me about anything that you liked about the website?
- Can you tell me about anything that you disliked?
- Can you tell me about anything that you would change in the website?
- Can you tell me about anything that you thought was particularly relevant to you?
- Can you tell me about anything that you thought was not particularly relevant to you?
- Can you tell me about any information or advice that didn't make sense?
- How do you think that could be changed?
- Could you tell me about anything that you thought didn't work properly?
- Can you tell me about whether you went onto the website more than once? (explore why they returned/whether they found what they needed).
- Since looking at the website, how do you feel about infections now?

Spray Instructions:

- On the website, it mentions 3 situations where you should use the spray. What did you think about these instructions?
- On the website, it mentions how often to use the spray in each of these 3 situations. What did you think about these instructions? (prompting around the instructions).
- On the website, there is a video about how to use the spray. What did you think about this?
- Can you tell me what you thought about the paper booklet about the spray? (repeat questions above as necessary- liked, disliked etc).

Open-ended Questions about personal experiences of RTIs:

1. Can you tell me all about your experience of these sorts of infections [repeat list of RTIs if necessary: colds, flu, coughs, chest infections, bronchitis, ear infections, sinusitis, sore throats, throat infections and tonsillitis].

- Prompts:
    - Can you tell me about the types of illnesses you tend to get?
    - Can you tell me about when you tend to get these illnesses?
    - What's it like for you when you have them?
    - How often do you get them?
    - How long do they last?

2. Can you tell me about why you think you get these sorts of illnesses?
    - Prompts:
        - Can you tell me about what you think the causes of these illness?
        - Any other reasons why you think you get them?
3. Can you tell me about things you do to try and stop getting these illnesses?
    - Prompts:
        - What made you decide to use these things? Why is it important for you to x/y/z? (e.g. eat healthy, exercise, get the flu jab)
        - How helpful do you find these things?
        - Why do you think they work?
4. When you have these sorts of illnesses is there anything you do to try and make it go away quicker?
    - Prompts
        - Any things you take, or things you do, or avoid doing?
        - What made you decide to use these things? Why is it important for you to x/y/z?
        - How helpful do you find these things?
        - Why do you think they work?

[*coronavirus question and probing was not in the original interview schedule and was added in for later interviews]
