# Supplementary material for: Using nasal sprays to prevent respiratory tract infections: a qualitative study of online consumer reviews and primary care patient interviews
Source: BMJ Open. 2022 Jun 30;12(6):e059661. doi: 10.1136/bmjopen-2021-059661 (PMC9247325; doi:10.1136/bmjopen-2021-059661)
Supplement: Supplementary data [file bmjopen-2021-059661supp005.pdf]

Supplementary material 5: COREQ checklist

Tong et al 2007, 32 item check list: https://academic.oup.com/inghc/article/19/6/349/1791966

		Item	Guide questions/description	Manuscript section where information can be found
**Domain 1: Research team and reflexivity**				
Personal Characteristics	1	Interviewer/facilitator	Which author/s conducted the interview or focus group?	Method- data collection – Phase B: Post-intervention interviews (page 8)
	2	Credentials	What was their occupation at the time of the study?	Method- data collection – Phase B: Post-intervention interviews (page 8)
	3	Occupation	What was their occupation at the time of the study?	Method- data collection – Phase B: Post-intervention interviews (page 8)
	4	Gender	Was the researcher male or female?	Method- data collection – Phase B: Post-intervention interviews (page 8)
	5	Experience and training	What experience or training did the researcher have?	Method- data collection – Phase B: Post-intervention interviews (page 8)
Relationship with participants	6	Relationship established	Was a relationship established prior to study commencement?	Method- recruitment – Study 2 (page 7)
	7	Participant knowledge of the interviewer	What did the participants know about the researcher? e.*g. personal goals, reasons for doing the research*	Method- recruitment – Study 2 (page 7)
	8	Interviewer characteristics	What characteristics were reported about the interviewer/facilitator? e.g. *Bias, assumptions, reasons and interests in the research topic*	Method- data collection (page 8)<br>Funding statement (page 25)
**Domain 2: study design**				
Theoretical framework	9	Methodological orientation and Theory	What methodological orientation was stated to underpin the study? *e.g. grounded theory, discourse analysis, ethnography, phenomenology, content analysis*	Method (page 6, page 7, page 9)
Participant selection	10	Sampling	How were participants selected? *e.g. purposive, convenience, consecutive, snowball*	Method- recruitment (page 7)
	11	Method of approach	How were participants approached? e.*g. face-to-face, telephone, mail, email*	Method- recruitment (page 7)
	12	Sample size	How many participants were in the study?	Method- recruitment (page 7)
	13	Non-participation	How many people refused to participate or dropped out? Reasons?	n/a

				Not reported in main manuscript for conciseness.<br><br>Our recruitment method does not allow us to know why participants did not respond to our invitation to participate.
	14.	Setting of data collection	Where was the data collected? e.*g. home, clinic, workplace*	Method- data collection (page 7, page 8)
	15.	Presence of non-participants	Was anyone else present besides the participants and researchers?	Not reported in main manuscript for conciseness.<br><br>Participants were asked to be in a quiet room with no interruptions but we do not know for sure if it was always possible as most interviews were via telephone. Field notes and interview recordings from one face-to-face interview suggest a spouse was present and commenting occasionally.
	16.	Description of sample	What are the important characteristics of the sample? *e.g. demographic data, date*	Findings – Table 2 (page 14)
Data collection	17.	Interview guide	Were questions, prompts, guides provided by the authors? Was it pilot tested?	Method- PPI section (page 9), data collection – post-intervention interviews (page 8); Supplementary Material 4
	18.	Repeat interviews	Were repeat interviews carried out? If yes, how many?	Method- data collection – post-intervention interviews (page 8), Table 2 (page 14).
	19.	Audio/visual recording	Did the research use audio or visual recording to collect the data?	Method- data collection – post-intervention interviews (page 8)
	20	Field notes	Were field notes made during and/or after the interview or focus group?	Method- data collection – post-intervention interviews (page 8)
	21.	Duration	What was the duration of the interviews or focus group?	Method- data collection – post-intervention interviews (page 8)
	22.	Data saturation	Was data saturation discussed?	Not reported in main manuscript for conciseness.<br><br>The authors are very cautious about claims of data saturation.<br>Data saturation for the current analysis was not aimed for but may have been achieved or approached.<br><br>Recruitment ceased when iterative intervention development was concluded i.e. the research team were satisfied that the interventions were as engaging

				as possible and a range of different viewpoints from patients with different clinical and demographic characteristics had been heard and used.
	23.	Transcripts returned	Were transcripts returned to participants for comment and/or correction?	n/a<br><br>(member checks with participants were not conducted, professional transcribers transcribed the interviews and researchers checked for accuracy)
**Domain 3: analysis and findings**				
Data analysis	24.	Number of data coders	How many data coders coded the data?	Method- data collection – analysis (page 7, page 9)
	25.	Description of the coding tree	Did authors provide a description of the coding tree?	Method- data collection – analysis (page 7, page 9) A coding tree was not used. We present a description of our process of inductive thematic analysis.
	26.	Derivation of themes	Were themes identified in advance or derived from the data?	Method- data collection – analysis (page 7, page 9)
	27.	Software	What software, if applicable, was used to manage the data?	Method- data collection – analysis (page 7, page 9)
	28.	Participant checking	Did participants provide feedback on the findings?	n/a<br><br>(member checks were not conducted)
Reporting	29.	Quotations presented	Were participant quotations presented to illustrate the themes / findings? Was each quotation identified? e*.g. participant number*	Findings Table 1 (page 12) and throughout the findings section.
	30.	Data and findings consistent	Was there consistency between the data presented and the findings?	Findings (page 10)
	31.	Clarity of major themes	Were major themes clearly presented in the findings?	Findings (page 10)
	32.	Clarity of minor themes	Is there a description of diverse cases or discussion of minor themes?	n/a
