# Supplementary material for: Using nasal sprays to prevent respiratory tract infections: a qualitative study of online consumer reviews and primary care patient interviews
Source: BMJ Open. 2022 Jun 30;12(6):e059661. doi: 10.1136/bmjopen-2021-059661 (PMC9247325; doi:10.1136/bmjopen-2021-059661)
Supplement: Supplementary data [file bmjopen-2021-059661supp006.pdf]

Supplementary material 6: Themes and how they were used for intervention development

**Study findings**<br><br>**Theme title (and study where it was identified)**	**Summary of theme/finding**	**"Immune Defence" nasal spray Intervention component/content**<br>(NB previous research, theory, stakeholder opinions also fed into these decisions alongside Study 1 and 2 findings)
Motivation to avoid infections (study 1)<br><br>Excitement and optimism about a novel prevention method (study 2)	High motivation to avoid RTI (for a range of health, work, social reasons)<br><br>Explanation of spray mechanism and ways of using generated interest, hope, willingness to try.	These findings, in conjunction with our target group characteristics (recurrent RTIs/vulnerable to RTIs) meant we decided to not include significant content to convince of the necessity of avoiding infections. We kept content about the impact of RTIs and necessity of avoiding them brief and used this section predominantly to show empathy, establish a connection with users and help convince them that the intervention was relevant to them.
Inevitability (study 1)	Beliefs/experiences that RTIs are inevitable and can't be prevented or course altered once they have begun	Acknowledge current feeling and experiences of lack of control/inevitability but then build a convincing rationale for how the spray provides a chance to prevent/avoid RTIS. Describe a novel, interesting, plausible mechanism that people can understand as working in a different way to current/past prevention strategies they may have tried and experienced as ineffective.
Alternative approaches to infection prevention (study 1)	Belief that other approaches are (more) helpful for preventing RTIs	Do not attempt to persuade that any specific existing behaviours/habits/prevention methods are unhelpful/unnecessary, but refer to overall experience of wanting to gain more control and protection from infections.<br><br>Position the spray as an extra protection measure (along with novelty message and convincing rationale about how it works).
Recommendations from others (study 1)	Other people's recommendations are important	Provide a strong message of recommendation. This is given authority by NHS, University involvement and 'meet the team' of experts page and reference to scientific research.

Protection from risky situations (study 1)<br><br>Considering use in risky situations (study 2)	Interest in using spray to protect oneself from RTIs in situations perceived to be high risk<br><br>Considerable interest in using spray to protect oneself from RTIs n situations perceived to be high risk, especially during COVID-19 pandemic.<br><br>Some ability to correctly identify high risk situations but also some difficulty/uncertainty, especially in the context of the COVID-19 pandemic and its restrictions/mitigations.	Provide a positive message about being able to take steps to protect yourself.<br><br>Help users identify high risk situations to use the spray in.<br>Provide examples of when to use the spray (using some examples that study participants spontaneously came up with – e.g. Public transport, aeroplanes, childcare/grandchildren situations).<br><br>Use follow-up intervention emails to revisit/remind about the types of situations and the ability of the spray to work in addition to existing mitigations. We had to edit intervention emails in real time to ensure situations and examples are well aligned with pandemic risk levels, lockdowns, restrictions.<br><br>Description of the spray as an extra layer of protection in addition to existing mitigations (e.g. Face coverings, handwashing).
Ease or difficulty (study 1)<br><br>Familiarity, confidence and information needs (study 2)	Participants vary in how easy or difficult they find using the spray. Overall, it is easy but some aspects of it require attention for best results.<br><br>Depending, in part, on past experiences of nasal sprays people may be under or over-confident in using the spray. This could lead to either anxiety about using the spray or failing to follow the instructions.	Persuasion (text) and demonstration (video) that the spray use is easy, quick and convenient.<br><br>Acknowledgment that it may take more than one use to perfect the technique (e.g. "After a few tries you will work out what feels comfortable for you", "you'll soon get the hang of it").<br><br>Clear instructions to ensure that it is experienced as easy and identified uncertainties and concerns are eliminated.<br><br>Short instructions, supplemented by optional more detail (website: drop down sections; booklet=short infographic style instructions plus reference to website for further information)<br><br>Persuasive text to stop people skipping important information by highlighting why it is useful/new (e.g. "check out this video to see how to use your nasal spray. This video is worth watching even if you have used nasal sprays before. The technique for **this spray**

		might be a bit different. Using the spray correctly give you the best chance of fighting infections!")<br><br>Inclusion of a suggestion to try the spray out when it arrives to boost confidence prior to time it is needed.
Experiencing side effects (study 1)<br><br>Reactions to possible/actual side effects (study 2)	Side effects of the spray are common, milder ones are tolerated if benefits are expected/experienced. Strong side effects can prevent further use.<br><br>Reassuring information about side effects is valued. People describe being willing to try the spray despite minor side effects. Severe side effects seem likely to influence discontinuation.	Explanation that side effects are minor<br><br>Framing of sensations in nose and throat (e.g. tingling, noticeable taste) as normal and a positive sign the spray is working/reaching the right place rather than a side effect.<br><br>Comparisons of mild side effects with more severe and prolonged symptoms of 'full blown infection'.<br><br>Instruction and demonstration on how to avoid the more severe side effects (spray technique).<br><br>Instructions on how to cope with side effects (e.g. position to adopt for nose bleeds, use of saline solution for dry/irritated nose, eating/drinking to eliminate unpleasant taste).
Identifying early signs of infection (Study 2)	Participants often but not always have awareness of first signs of infection and confidence in being able to use the spray in response	Give sufficient information about which first signs are relevant by listing main signs that people recognise as relevant to RTIs (feeling in throat, malaise) but also allowing for idiosyncratic first signs.<br><br>Acknowledge the difficulty distinguishing some symptoms (e.g. Runny nose, sneezing - hayfever & RTI overlap).<br><br>Explain and reassure that it would be advisable and safe to use on a symptom that turned out not to be an RTI symptom.<br><br>Given that we know people may miss first signs, refer to failure to act quickly enough as a possible explanation for situations where

		the spray does not appear to have helped (to try to prevent users from concluding that the spray is ineffective).
Expectations and experiences of success and failure (study 1)<br><br>Excitement and Optimism about a novel prevention method (study 2)	Users experience combinations of success and failure with the spray which then influence the continuation of use.<br><br>Idea of spray elicits interest, hope, willingness to try. For some this is very pronounced, for others it is more muted or sceptical.	Promote excitement, interest, positivity and expectations of success (via convincing explanation of how it works)<br><br>Provide a rationale for how spray use might lead to partial success (not avoiding infections but having shorter and less severe).<br><br>Provide a rationale for why it might not always work (using too late)<br><br>Encourage persistence if it does not appear to work, including attributing failure to using it too late and explaining that it may nonetheless have reduced infection severity and duration.<br><br>Encourage formation of the spray as a low risk / safe / easy intervention suitable for regular use in order to help them to conclude that potential for benefit outweighs concerns, even if they do not experience clear cut evidence of success (i.e. It is like a hand gel, not a medicine, and with no serious side effects)
Excitement and optimism about a novel prevention method (study 2)<br><br>Consequences of feeling protected (study 2)	Idea of spray elicits interest, hope, willingness to try. For some this is very pronounced, for others it is more muted or sceptical.<br><br>Feeling protected may make people feel safer, more confident and more able to participate in valued activities. It could also make people take more risks.	Boost positive expectancies of the spray's psychosocial effects including how the spray can make you feel more confident and in control and that it feels good to feel more protected [emails]<br><br>Alongside positive expectations of spray efficacy, promote continued adherence to COVID-19 regulations/guidance/mitigations; positioning the spray as an additional, not a replacement behaviour.<br><br>Do not refer to or recommend against any current infection control behaviours (e.g. Keeping distance from ill people, good respiratory hygiene, good diet, being physically fit)
Concern about medicines (Study 2)	People see nasal sprays as a medicine, eliciting medication-related	Explicitly position the spray as not a medicine, whilst maintaining expectations that it will be a powerful and effective product.

	concerns such as overuse, allergies, contraindications	Compare it instead to hand gel (noting the similar mechanisms- cleaning away virus before it can cause illness).<br><br>Provide reassuring information about how often it is safe to use it and how it can be used with any prescription and over-the-counter medications.<br><br>Position spray as a means of avoiding using medications such as antibiotics, over-the-counter cold relief.<br><br>NB- we expect concerns related to medicines to persist to some degree in some participants despite our 'not a medicine' message. The spray might, to a layperson, feel like a medicine in terms of its mode of administration and anticipated efficacy. Our content nonetheless promotes beliefs about it being a simple, safe and effective intervention.
Disgust and hygiene (Study 2)	Noses and nasal sprays can be considered disgusting and/or messy and unhygienic	Reassurance that the spray procedure is not wet, messy or unpleasant.<br><br>This required a change (between study 2a and study 2b) from our original description of the spray being not like a medicine but like washing hands with soap and water. We adopted a neater/cleaner explanation (like hand gel). The public were becoming very familiar with hand gel as an important infection control product at this point in the COVID-19 pandemic.<br><br>Emphasise easiness of using the spray.<br><br>Instructions on how to use hygienically.
