# Supplementary material for: Using nasal sprays to prevent respiratory tract infections: a qualitative study of online consumer reviews and primary care patient interviews
Source: BMJ Open. 2022 Jun 30;12(6):e059661. doi: 10.1136/bmjopen-2021-059661 (PMC9247325; doi:10.1136/bmjopen-2021-059661)
Supplement: Author's manuscript [file bmjopen-2021-059661.draft_revisions.pdf]

BMJ Open is committed to open peer review. As part of this commitment we make the peer review history of every article we publish publicly available.

When an article is published we post the peer reviewers' comments and the authors' responses online. We also post the versions of the paper that were used during peer review. These are the versions that the peer review comments apply to.

The versions of the paper that follow are the versions that were submitted during the peer review process. They are not the versions of record or the final published versions. They should not be cited or distributed as the published version of this manuscript.

BMJ Open is an open access journal and the full, final, typeset and author-corrected version of record of the manuscript is available on our site with no access controls, subscription charges or pay-per-view fees (http://bmjopen.bmj.com).

If you have any questions on BMJ Open's open peer review process please email info.bmjopen@bmj.com

BMJ Open

Using nasal sprays to prevent respiratory tract infections: A QUALITATIVE STUDY OF ONLINE CONSUMER REVIEWS AND PRIMARY CARE PATIENT INTERVIEWS

Journal:	*BMJ Open*
Manuscript ID	bmjopen-2021-059661
Article Type:	Original research
Date Submitted by the Author:	07-Dec-2021
Complete List of Authors:	Williamson, Sian; University of Southampton, School of Psychology<br>Dennison, Laura; University of Southampton School of Psychology, Psychology<br>Greenwell, Kate; University of Southampton School of Psychology,<br>Denison-Day, James; University of Southampton<br>Mowbray, Fiona; University of Southampton School of Psychology,<br>Richards-Hall, Samantha; No institution, Patient and Public Contributor<br>Smith, Deb; No institution, Patient and Public Contributor<br>Bradbury, Katherine; University of Southampton, Psychology<br>Ainsworth, Ben; University of Bath, Department of Psychology; University of Southampton<br>Little, Paul; University of Southampton, Primary Care and Population Science;<br>Geraghty, Adam; University of Southampton, Primary Care and Population Sciences<br>Yardley, Lucy; University of Southampton, Psychology; University of Bristol, School of Health Sciences
Keywords:	QUALITATIVE RESEARCH, Respiratory infections < THORACIC MEDICINE, PREVENTIVE MEDICINE

SCHOLARONE™
Manuscripts

BMJ

I, the Submitting Author has the right to grant and does grant on behalf of all authors of the Work (as defined in the below author licence), an exclusive licence and/or a non-exclusive licence for contributions from authors who are: i) UK Crown employees; ii) where BMJ has agreed a CC-BY licence shall apply, and/or iii) in accordance with the terms applicable for US Federal Government officers or employees acting as part of their official duties; on a worldwide, perpetual, irrevocable, royalty-free basis to BMJ Publishing Group Ltd ("BMJ") its licensees and where the relevant Journal is co-owned by BMJ to the co-owners of the Journal, to publish the Work in this journal and any other BMJ products and to exploit all rights, as set out in our licence.

The Submitting Author accepts and understands that any supply made under these terms is made by BMJ to the Submitting Author unless you are acting as an employee on behalf of your employer or a postgraduate student of an affiliated institution which is paying any applicable article publishing charge ("APC") for Open Access articles. Where the Submitting Author wishes to make the Work available on an Open Access basis (and intends to pay the relevant APC), the terms of reuse of such Open Access shall be governed by a Creative Commons licence – details of these licences and which Creative Commons licence will apply to this Work are set out in our licence referred to above.

Other than as permitted in any relevant BMJ Author's Self Archiving Policies, I confirm this Work has not been accepted for publication elsewhere, is not being considered for publication elsewhere and does not duplicate material already published. I confirm all authors consent to publication of this Work and authorise the granting of this licence.

For peer review only

USING NASAL SPRAYS TO PREVENT RESPIRATORY TRACT INFECTIONS: A QUALITATIVE STUDY OF ONLINE CONSUMER REVIEWS AND PRIMARY CARE PATIENT INTERVIEWS

Sian Williamson*[1], Laura Dennison*[1], Kate Greenwell[1], James Dennison-Day[1],, Fiona Mowbray[1], Samantha Richards-Hall[5]; Debs Smith[5], Katherine Bradbury[1,4], Ben Ainsworth[6], Paul Little[2] and Adam W A Geraghty[2] Lucy Yardley[1,3]

[1]Centre for Clinical and Community Applications of Health Psychology, Faculty of Environmental and Life Sciences, University of Southampton, Southampton, UK; [2]School of Primary Care, Population Sciences and Medical Education, Faculty of Medicine, University of Southampton, Southampton, UK; [3]School of Psychological Science, University of Bristol, Bristol, UK. [4]NIHR Applied Research Collaboration (ARC) Wessex, Southampton, UK. [5] No affiliation (Patient and Public Contributors), [6] Department of Psychology, University of Bath.

*Joint First Authors

Corresponding Author: Laura Dennison, School of Psychology, University of Southampton, Highfield Campus, Southampton, SO17 1BJ, l.k.dennison@soton.ac.uk

For peer review only

Word count (excluding title page, abstract, references, figures and tables)=5189

Keywords: Respiratory Tract Infections, Common Cold, Influenza, Prevention, Qualitative Research, Interviews, Thematic Analysis

ABSTRACT

Objectives: Nasal sprays could be a promising approach to preventing Respiratory Tract Infections (RTIs). This study explored lay people's perceptions and experiences of using nasal sprays to prevent RTIs to identify barriers and facilitators to their adoption and continued use.

Design: Qualitative research.

Setting: Primary Care, UK.

Methods: Study 1 thematically analysed 407 online consumer reviews of a RTI prevention nasal spray. Study 2 purposively recruited 13 primary care patients who experience recurrent infections and/or risk factors for severe infections. They were interviewed about their reactions to and experiences of a digital intervention that promotes and supports nasal spray use for RTI prevention (reactively: at 'first signs' of infection and preventatively: following possible/probable exposure to infection). Interview transcripts were analysed using thematic analysis.

Findings: Both studies identified various factors that might influence nasal spray use including: high motivation to avoid RTIs, particularly during the COVID-19 pandemic; fatalistic views about RTIs; beliefs about alternative prevention methods; the importance of personal recommendation; perceived complexity and familiarity of nasal sprays; personal experiences of spray success or failure; tolerable and off-putting side effects; concerns about medicines; and the nose as unpleasant and unhygienic.

Conclusions: People who suffer disruptive, frequent or severe RTIs or who are vulnerable to RTIs are interested in using a nasal spray for prevention. They also have doubts and concerns and may encounter problems. Some of these may be reduced or eliminated by providing nasal sprays users with information and advice that addresses these concerns or helps people overcome difficulties.

For peer review only - http://bmjopen.bmj.com/site/about/guidelines.xhtml

ARTICLE SUMMARY: STRENGTHS AND LIMITATIONS OF THIS STUDY

- This is the first research about how people think and feel about using a nasal spray to prevent respiratory tract infections (RTIs) so adopting an exploratory, inductive, qualitative approach allowed insight into key issues that are important to the population studied.

- The paper benefits from its coverage of multiple *populations* (people who had already purchased and used the spray, primary care patients with relevant health conditions, healthcare use and/or RTI history), *data collection approaches* (online product reviews, think-aloud interviews and post-intervention interviews) and *contexts* (before COVID-19 and during the early months of the pandemic).

- The pandemic context (social distancing restrictions, shielding), short study period (2-3 weeks) and season (summer) meant Study 2 participants had little exposure to viruses and limited opportunities to try out their sprays.

- Although Study 2 participants varied in terms of number and types of RTI, chronic health conditions and some demographic characteristics the sample lacked ethnic diversity, tended to have low levels of deprivation, and consisted of more females than men.

- Nesting the current research within the development of a digital intervention ("Immune Defence") to support nasal spray use allowed key issues relating to the acceptability and engagement with our intervention to be gathered, interpreted and addressed; this paper therefore demonstrates the benefit of conducting in-depth qualitative research with target users during intervention planning, development and refinement.

For peer review only

INTRODUCTION

Respiratory tract infections (RTIs) such as the common cold, influenza, bronchitis, tonsillitis and sinusitis are commonly experienced by most adults. RTIs are usually caused by viruses and tend to be self-limiting. However, these illnesses are experienced as disruptive and unpleasant[1-5] and they are a key cause of workplace sickness absence[6]. RTIs also contribute significantly to pressures on primary care[7 8] and consultations for RTIs also result in unnecessary antibiotics prescriptions, thus contributing to antibiotic resistance[9 10]. Older adults and people with chronic conditions such as asthma or COPD experience higher morbidity and mortality from RTIs[11 12]. For these reasons, efforts to prevent common RTIs are essential. Furthermore, prevention measures are crucial during pandemics (e.g. COVID-19) to reduce mortality, morbidity and social, educational and economic disruption.

Some RTI prevention approaches reduce the likelihood of transmission and initial infection, such as social distancing[13], face-coverings[13] and handwashing[13 14]. Other prevention methods try to improve individuals' immune responses to viruses, for example through vaccination[15-17], nutrition[18 19], physical activity[20 21], and stress reduction[21]. Prevention approaches can also target the nose and the mouth as entry points for viruses, which are transmitted by inhalation or through contact with infected droplets. These approaches include mouthwashes and rinses and nasal sprays, douches, and irrigation regularly or pre/post exposure to possible/probable infection. The purported mechanisms involve either blocking or washing out virus and/or changing the environment of the nose and/or throat to reduce the risk the virus will survive/thrive. These approaches could either prevent infection entirely or reduce the severity of any infection which occurs by reducing viral load. Although some of these approaches have been commonplace in certain countries for many years, the COVID-19 pandemic has brought about a resurgence of interest in these approaches to prevention[22-28]. Many formulations and products are under investigation, with some promising findings and ongoing randomised-controlled-trials. For example, the RECUR trial (ICTRN17936080) is evaluating preventative use of nasal sprays to reduce the frequency, duration and severity of non-pandemic RTIs in recurrent and at-risk primary care patients whilst the ICE-COVID trial[24]

For peer review only - http://bmjopen.bmj.com/site/about/guidelines.xhtml

evaluates throat and nasal sprays for COVID-19 prevention in healthcare professionals.

As evidence on the efficacy of nasal sprays and related products and procedures grows, it is also essential to accrue evidence on the acceptability of these for the people who may be encouraged to adopt them. Much of the existing research on nasal sprays (and some of the research on nasal irrigation, mouthwashes, rinses) comes from laboratory studies. To be effective in real world conditions, human thought and behaviour, including acceptance and adherence, is critical. A nasal spray or rinse with 100% efficacy in vitro will less effective or ineffective if there is low uptake or sub-optimal use when implemented in real world settings. Some researchers appear optimistic about the simplicity of these approaches. For example, Kramer and colleagues[23] describe nasal rinsing as "easily implementable" as a public health measure for the prevention and control of COVID-19. However, lay people/patients may not find these approaches easy or acceptable[29]. They may have concerns or encounter barriers to adopting or persisting. Identifying these concerns and difficulties (along with more positive beliefs and experiences) would allow patient education and support to be tailored to include persuasive and reassuring messages and appropriate support to help people overcome barriers.

So far, no published research has investigated views or experiences of using nasal sprays, rinses and mouthwashes for preventing RTIs. However, there is some research on interventions that share similar characteristics in the context of managing symptoms or conditions. People with chronic rhinosinusitis describe problems with using steroid nasal sprays including difficulties remembering to use them, and confusion or lack of confidence with spray technique[30]. They may consider nasal irrigation (a procedure where a saline solution is poured in and out of the nostrils) awkward and prohibitively time-consuming[30]. Nasal irrigation can also cause discomfort or even pain in some chronic or recurrent sinusitis patients; which they balance against perceived symptom reduction; 'putting up' with it if beneficial[4]. These patients also describe using management approaches irregularly and stopping once relief is gained[4]. Furthermore, COVID-19 outpatients managed to self-administer a povidone iodine solution through nasal pulverisation 4 times per day for 5 days but experienced unpleasant nasal tingling[31]. Together, these studies indicate that RTI prevention strategies requiring nasal application of a substance may be off-

For peer review only - http://bmjopen.bmj.com/site/about/guidelines.xhtml

putting for at least some patients and regular, long-term persistence may difficult to achieve.

This paper extends the literature by investigating people's perceptions and experiences of using a nasal spray for the purpose of RTI prevention. We report findings from two qualitative studies nested within the RECUR research programme which focuses on the development and evaluation of interventions to prevent RTIs. The first study reported in this paper is an analysis of online customer reviews of a RTI prevention nasal spray. The second study analyses interviews with patients heavily burdened by and/or at higher risk from RTIs about their perceptions of and experiences of using a nasal spray for RTI prevention. Our aim for both studies was to explore how people think and feel about using nasal sprays to prevent RTIs and to identify barriers and facilitators to the adoption and continued use of sprays. The findings will be valuable to researchers and clinicians seeking to develop or implement RTI prevention approaches, especially those involving nasal sprays or similar prophylactic products such as nasal and mouth rinses and washes.

METHOD

Intervention development context

The studies reported in this paper were undertaken as part of the development of a digital behavioural intervention to encourage and support people to use a nasal spray to prevent RTIs (NIHR programme grant RP-PG-0218-20005; 'RECUR'). A randomised-controlled-trial is currently evaluating the efficacy of the nasal spray intervention; within the trial the brand name of the spray is masked. Therefore, this paper simply refers to it as 'the nasal spray'. The manufacturer instructions advise use at the first signs of a cold. In the intervention under evaluation, participants are also advised to use the spray at first signs of *any* suspected RTI and also in situations where exposure to RTIs is likely (e.g., crowded places, close proximity to infected people).

The intervention development work used the person-based approach[29], which prioritises in-depth qualitative data collection to explore the views and experiences of

potential intervention users, in order to understand the context in which users engage with interventions and behaviour change. Figure 1 shows how the studies reported here were used alongside primary qualitative research[32], a scoping review, behaviour change theory (Protection Motivation Theory[33 34], Social Cognitive Theory[35], Necessity-Concerns Framework[36 37], Sense Model[38 39]) and stakeholder and PPI involvement to develop and optimise the intervention. The two studies reported here influenced the development of 'guiding principles'[29] (Supplementary Materials 1) and the articulation of programme theory through a logic model for the intervention[40 41](Supplementary Materials 2), then enabled iterative changes to the intervention (Supplementary Materials 3).

Figure 1: overview of nasal spray intervention development activities.

[INSERT FIGURE 1 HERE]

Study 1: online consumer reviews of the nasal spray

Data collection

407 customer reviews of the nasal spray were taken from four large, commercial websites (comprising 263, 93, 30 and 21 spray reviews each). The websites were selected based upon having a large number of spray reviews. All reviews were included (positive, negative) except those which focused on supplier-based issues (e.g. damaged product). We also removed reviews that were duplicated across websites. The search for reviews was conducted in August 2019.

Analysis

We used an inductive thematic analysis approach. Although the review data was 'thin' and brief (typically several sentences for each review) we selected this approach to remain open and explorative and to generate broad themes that summarised important topics. Coding was undertaken by SW and FM who separately coded half of the reviews each in NVivo12 and then worked together to review, combine, discuss and refine coding. They then developed preliminary descriptive themes to capture key issues within the data. These were subsequently inspected, reorganised and relabelled by LD and SW.

For peer review only

Study 2: Interviews about using a nasal spray to prevent RTIs

Recruitment

We sought participants who experience frequent or recurrent infections and/or who are at risk of more severe RTIs. Three UK GP practices identified possible participants by searching their patient lists and posting invitations and information sheets to patients who consulted for ≥1 RTI within the last year and were prescribed antibiotics. They also wrote to patients who had asthma, COPD or chronic sinusitis who were at higher risk of RTIs. Patients interested in participating returned reply slips, on which they self-reported their recent RTI history. We then purposively sampled from these responses to prioritise interviewing those with higher RTI frequency and co-morbid health conditions. We also sought variation with regards age and gender. We interviewed 13 participants in total.

Data collection

Interviews took place from April to August 2020, coinciding with the beginning of COVID-19 pandemic. Consequently, interviews were conducted by telephone. Participants provided written consent prior to taking part. Before the interview, participants answered brief questions about demographics and the number and type of RTIs they experienced.

Phase A: Think aloud interviews (n=10).

Participants were emailed a link to our prototype web-based intervention promoting nasal spray use for RTI prevention (Figure 2 provides an overview of this intervention). They worked through the website whilst simultaneously sharing their reactions aloud. The researcher prompted them to verbalise their thoughts and feelings as they encountered different pages, sections, messages, images and features.

Phase B: Post-intervention interviews (n=7)

Participants were emailed a link to the digital intervention (now optimised based on phase A feedback). A nasal spray was posted to them along with a short booklet summarising spray instructions. They were asked to use the website and the spray independently over a period of 2-3 weeks They then participated in an in-depth interview about their experiences. All participants also answered open-ended

For peer review only

questions about their personal experiences of RTIs; findings from this part of the interview are published elsewhere[32].

Supplementary Material 4 contains the interview schedules. SW and LD conducted the interviews; both are female postdoctoral researchers with health psychology and qualitative interviewing expertise. Interviews lasted between 46 to 104 minutes and were audio-recorded and transcribed verbatim with identifying details removed. Participants received a £10 voucher to thank them for their time.

Figure 2: Overview of nasal spray intervention

[INSERT FIGURE 2 HERE]

Analysis

We used an inductive thematic analysis approach, broadly corresponding to that described by Braun & Clarke[42]. Transcripts from phase A and B were analysed together. The analysts familiarised themselves with the audio-recordings and transcripts. Line-by-line coding of the data was conducted in Nvivo12 whereby codes were identified and labelled to capture references to perceptions or experiences of nasal sprays for preventing RTIs. The codes were then reviewed, compared, discussed and progressively clustered and merged in order to create themes. This was an iterative process which progressed to refining and organising final themes that captured important patterns and features in the data. SW and LD led the analysis, and all other authors were involved in interpreting, discussing and finalising themes. The research team have health psychology and medical backgrounds and the lead analysts are experienced qualitative researchers.

Ethics approval

For study 1, ethics and research governance approvals were granted by the University of Southampton (ERGOII:52394).

For study 2, ethics approvals were granted by NHS and the University of Southampton review boards (REC/HRA19/SC/0354; ERGO:48223).

For peer review only

Patient and Public Involvement (PPI)

A panel of PPI contributors with experience of recurrent RTIs and/or health conditions that mean they are vulnerable to frequent or severe infections have inputted into the study planning and conduct, some from the grant application stage. Contributions included editing and improving our participant information sheets, consent forms and interview schedules and participating in pilot interviews helping to interpret findings and drafting this paper and lay summary of the research findings sent to participants. Two members of the PPI panel are co-authors on this paper (DS and SRH).

This research has been reported in line with the COREQ checklist (Supplementary Material 5)

FINDINGS

Study 1: online consumer reviews of the nasal spray

Eight themes about nasal spray experiences were developed from the customer review data. These are described below and supporting quotations are provided in Table 1. The wording of illustrative quotations has been edited slightly to prevent the original reviews and reviewers being identifiable (e.g., through entering the quotation into a search engine). SW reworded the quotations, keeping meaning as close to the original as possible. LD checked and further edited reworded quotes to ensure it retained the meaning and could not be traced back to the original review.

Motivation to avoid infections

Reviewers described strong motivations to avoid becoming ill with cold-like illnesses. For some this was to avoid disruption to responsibilities and routines. Others were focused on avoiding unpleasant or severe symptoms or health complications for themselves or others that they might infect (e.g. vulnerable family members).

For peer review only

Inevitability of infections

Some reviewers were fatalistic about catching colds and similar infections and believed that symptoms would inevitably develop and progress despite using the spray.

Alternative approaches to infection prevention

Some reviewers described alternative, competing or perceived superior approaches to avoiding RTIs. This included measures such as good hand hygiene, healthy eating and vitamin supplements. Some expressed a perceived confidence in the body's own ability to fight off infections.

Recommendations from others

Reviewers sometimes described being influenced to buy and try the spray because of success stories and recommendations from others such as friends, family or healthcare professionals.

Protection from risky situations

Some reviewers described adapting the way that the spray was used, beyond first signs and symptoms of an infection (i.e. recommended use as advised on product instructions). They adopted it as a preventative measure for when they perceived a high threat of infection, for example when travelling or in busy public places.

Ease of spray use

Reviewers often described sprays as quick and convenient to use and easily incorporated into daily life. However, some drew attention to the importance for correct technique and timely usage for efficacy. Some found that this is not always easily achievable.

Experiencing side effects

Reviewers commonly reported side effects including an unpleasant taste or feel in throat or nose, sinus pain, headache, or watery eyes. Side effects differed in severity across reviewers. When describing side effects, reviewers often referred to weighing up the experience of side effects against the impact of having a cold-like infection, reaching a range of conclusions about which was most desirable.

For peer review only

Expectations and experiences of success and failure

Some reviewers expressed confidence in the efficacy of the spray and referred to its ability to completely prevent colds and flu from developing or at least reduce the severity of symptoms and shorten their duration. Some reported lack of success or inconsistent results whereby sometimes infections happened despite use (although sometimes these were perceived as possibly milder than they would have otherwise been). Some reviewers emphasised the difficulties in judging whether the spray worked or not, given that it was uncertain how symptoms would have developed over time without spray use. However, doubts and uncertainties did not necessarily deter future use.

For peer review only

Table 1: themes from Study 1

Theme	Illustrative Quotations
Motivation to avoid infections	"As a mum, I can't afford to be ill – so it's wonderful that I now don't even though the rest of the family do."<br><br>"Because of my COPD I have to be careful cos colds can turn into a chest infection."
Inevitability of infections	"In my opinion, when you've got a cold there is no way to stop it."
Alternative approaches to infection prevention	"My body would probably have got rid of the cold – it usually does with vitamin c, drinking honey and using a salt water spray for my nose."<br><br>"In my opinion, if you don't touch your face (mouth, eyes and nose), this will prevent a cold. Germs live on surfaces for hours, so we need to be aware of this when we are out and about but especially if any of our family have an infection."
Recommendations from others	"I bought the spray because a nurse recommended it."<br><br>"My husband is a strong believer in this stuff."

For peer review only

Protection from risky situations	"I use it for the Tube where lots of people might be unwell - sneezing and stuff. The spray says to use it for when you have a cold coming but I have been using it every day regardless."<br><br>"I purchased it for when I go on holiday, when I usually catch infections when travelling by airplane. Since using it, I've not had any colds on my last two trips."
Ease of spray use	"The spray is easy to use and you can take it anywhere with you. I don't go anywhere without it."<br><br>"The instructions say you should aim towards your ear, and I thought I did do that. It's difficult to do it right."<br><br>"If you don't catch you first signs really early (e.g. the first odd feeling like tickling in the back of your throat) it will be too late. If your nose is already stuffy, it probably won't work."<br><br>"You must use the spray for a couple of days after your symptoms have gone away. If you stop when your symptoms are improving, your infection comes back."
Experiencing side effects	"The negative part is throat pain for 5 minutes or so, but that's the only negative. It's really bad pain but it's worth it to avoid getting a cold."<br><br>"I had extreme side effects. I don't want to have them again so I got rid of it. I reckon it works but the side effects were too bad for me!"
Expectations and experiences of success and failure	"Since the start of the year, I'd been unwell all the time. Then I used the spray at first signs and it stopped my cold (or at least made it tolerable and easier to deal with)."<br><br>"I've used the spray before and believed it had stopped my colds. However, it failed this time even though I followed the instructions exactly! The cold was the worst I've had in ages so now I just don't know if the spray DID work when I used it before."<br><br>"There's no way to be sure if my infection would have continued to get worse without the spray but, if there's any chance it was crucial in stopping the cold, then it's worth it!"

Study 2: Interviews about using a nasal spray to prevent RTIs

Participants

Table 2 describes the characteristics of the Study 2 participants. Their ages ranged from 18 to 83, with an average of 54. More women than men participated. Participants reported a mean of 2.9 infections in the previous year, and around half experienced ≥3 RTIs per year over the last 3 years.

For peer review only

Table 2: Demographic and clinical characteristics of Study 2 participants (n=13)

Characteristic	Summary Statistics
**Type of interview participation, N(%)**	
Think aloud interview only	8 (61.54%)
Post intervention interview only	3 (23.08%)
Both think aloud and post intervention	2 (15.38%)
**Age (years), mean (SD), range**	54.34 (22.24), 18-83
**Gender, N(%)**	
Men	3 (23.1%)
Women	10 (76.9%)
**Marital status, N(%)**	
Married or living with partner	5 (38.46%)
Single	3 (23.08%)
Divorced	2 (15.38%)
Widowed	3 (23.08%)
**Employment status, N(%)**	
In paid work (full or part time, employed, self-employed)	4 (30.77 %)
Retired	4 (30.77%)
Not working because of illness/disability	2 (15.38%)
Other (unemployed, homemaker, student)	3 (23.08%)
**Education (age left education), N(%)**	
16 or before	2 (15.38%)
17 or 18	3 (23.08%)
Over 18	8 (61.54%)
**Deprivation (IMD[1]), MDn (IQR), range**	10 (6.0), 3-10
**Ethnicity, N(%)**	
White British	7 (53.85%)
White Irish	1 (7.69%)
Mixed- White British/Asian	1 (7.69%)
Not provided	4 (30.77%)
**Health Conditions, N(%)** [2]	
Asthma	6 (46.15%)
COPD	2 (15.38%)
Chronic Sinusitis	1 (7.69%)
None of these conditions	7 (53.85%)

**Number of RTIs in last 12 months, Mean (SD), range**	2.92 (1.38), 1-5
**RTIs per year in last 3 years, N(%)**	
≥1	12 (92.31%)
≥3	7 (53.85%)
**Types of RTIs experienced at least once in last 12 months, N(%)**	
Cold	10 (76.92%)
Flu	2 (15.38%)
Throat infection	9 (69.23%)
Chest infection	7 (53.85%)
Sinus infection	6 (46.15%)
Ear infection	3 (23.08%)

[1]IMD= 2019 Index of Multiple Deprivation Decile derived from participant postcodes, 1 is highest deprivation, 10 is lowest deprivation

2. The percentage totals more than 100 because 2 participants (15.38%) had more than one of these conditions

Themes

Eight themes were developed (Table 3). These are described below alongside supporting quotations.

For peer review only

Table 3: Themes from Study 2

Theme	Illustrative Quotations
Excitement and optimism about a novel prevention method	"Then, when this came along it was like lightbulbs going off. I'm thinking, oh my God, this is going to be a way that I can safeguard myself and continue to be active within his life. I'm really excited about the uses of it." (Participant 10)<br>"I would quite happily give it a go." (Participant 11)<br>"A hundred per cent I'd be up for giving it a go." (Participant 5)<br>"I will give it a go I can tell you that now." (Participant 6)
Identifying first signs of infection	"I tend to just feel more rundown, tired, a bit headachy." (Participant 7)<br>"A lot of the times when I'm sneezing it's just because of my hay fever. It was quite difficult to tell." (Participant 9)<br>"If I know it's coming, by the time I'm doing something about it, I guess because my immune system's got no great strength, it's almost like too little, too late." (Participant 10)
Considering use in risky situations	"I can say, 'Well, I've got to go out. There's a chance I may be in contact with other people, so I'll use the spray'. It's like another layer of protection." (Participant 12)<br>"COVID-19 makes it more appealing, actually. I was quite intrigued about whether it would work for COVID." (Participant 11)<br>"I don't know, on a bus, supermarket, at the cinema, at the theatre… Like when you get home from the theatre you could start using it then, even if you haven't had any signs of anything. That was something that I thought was really useful to have. I could see that scenario." (Participant 10)
Consequences of feeling protected	"...that could only be helpful. I'm genuinely interested from those points of view, because I could get protection in the small part of my life where I'm allowing myself to be at risk, plus I think if I felt safer, I might therefore go out more and feel less frightened about the world." (Participant 10)<br>"It just meant that I could get on with things. Did I feel invincible? No, but I felt like I didn't have to worry too much, whereas I think if I was coming down with a cold I would have worried about work and being ill and not being able to complete work. I felt more relaxed, maybe, more confident." (Participant 11)<br>"But then would it encourage more people to actually go out and be slightly more reckless with sprays and masks and protection, washing their hands, touching their face because they're going, 'Oh, I'm using the spray, it's okay'. That's the other side of it." (Participant 12)

For peer review only

Concerns about medicines	“Part of it is because I don't like using medications, and I particularly don't like nasal sprays. I think over the last year or so I've used far too many and I'm a bit fed up of putting things in my nose. I think there's something off-putting about that.” (Participant 11)<br><br>“I mean, to be fair, if it worked and it stopped me taking my medication, I'd much rather use a spray than medicine.” (Participant 8)<br><br>“At the same time, I was like, oh, well if you don't have to ask a doctor and it's not an actual medication is it actually going to work?” (Participant 9)
Unpleasantness and hygiene	“It's not particularly pleasant, is it, watching people sticking things up their noses and their noses run and stuff.” (Participant 11)<br><br>“You spray it up and then it all runs down. That sounds disgusting.” (Participant 4)<br><br>“I was also worried that if I used it, it would pour everywhere. It didn't really.” (Participant 9)<br><br>“I wouldn't [re]use anything that went into an orifice like an inhaler, or something I stuck up my nose, I wouldn't keep it and use it for another time.” (Participant 10)
Familiarity and confidence	“I'm not very good at stuff like that. …I don't think I've ever really tried one [a nasal spray]. I'm just kind of wary of it.” (Participant 9)<br><br>“It's common sense really. I've been using a [different type of] spray for years.” (Participant 4)<br><br>“It's so straightforward using a nasal spray… I wouldn't bother with the video… Particularly at my age range, you've probably used nasal sprays several if not many times over your lifetime so you just would just use it.” (Participant 1)<br><br>“I think [I was] probably arrogant, I probably thought, 'Oh, for goodness sake, I don't need to be shown how to use a nasal spray!"' Although, clearly I did because once I used it as recommended, I didn't get a headache.” (Participant 13)
Reactions to possible or actual side effects	“I think it's good that it's listing the side effects, but they're not severe side effects. Obviously, if they're only very, very small, like causing a headache, you can take some paracetamol for that. If it stops you getting an infection or prolonging the infection, then a headache, just stopping that is very minor.” (Participant 5)<br><br>“I'd rather have that then a full-blown infection. That is on the plus side, even if it can cause a headache.” (Participant 8)<br><br>“I thought I'd try it again, and I did aim it more towards the ear, and although I did get a slight headache, it was much better and it went away very quickly.” (Participant 13).

For peer review only - http://bmjopen.bmj.com/site/about/guidelines.xhtml

Excitement and optimism about a novel prevention method

Overall, participants described positive and optimistic views about using the spray, seeing it as novel and of interest and personal relevance. For a few participants, there was a very pronounced excitement, with the spray seen as a way of transforming their quality of life. Others were more muted in their enthusiasm but still interested and willing to try the spray. Even participants who were not fully convinced that the spray would work, still considered it worth a try.

Participants found the explanations in the Immune Defence digital intervention about how the spray works to be understandable and plausible, in particular how the spray created an inhospitable environment for viruses. These ideas were particularly relevant and persuasive based on understandings about viruses and infection that participants were rapidly developing during the COVID-19 pandemic.

Identifying first signs of infection

Most participants were aware of their early RTI signs and felt able to recognise and react promptly to them by using a spray. However, sometimes participants found it difficult to tell whether a symptom signalled an oncoming infection. The crossover between hay fever and RTI symptoms was a particular area of uncertainty.

A minority of participants also described how they never experienced common early signs of infection and only became aware of oncoming illness through a severe symptom typical of a later stage of an infection (e.g., cough). Some therefore anticipated struggling to intervene in time.

Considering use in risky situations

Participants were particularly interested in using the spray in risky situations to prevent infections. Some participants considered that this mode of use may help to protect against COVID-19, although some remained cautious.

Some participants easily identified risky situations, where they would be happy to use the spray preventatively such as supermarkets, hospital appointments, concerts, airplanes and public transport. However, other participants debated or expressed

For peer review only

uncertainty about what level of exposure would count as 'risky'. For some, most situations were currently considered risky (i.e., during the COVID pandemic). Others felt that if other mitigations were in place (such as social distancing or face masks) the spray was redundant for RTI prevention.

Consequences of feeling protected

A few participants anticipated that the protection against RTIs afforded by the spray would change how they felt, thought and behaved including feeling safer, less fearful more relaxed and more comfortable mixing with people with RTIs. A minority expressed concern that using the spray could lead to negative consequences for infection prevention behaviours. They speculated that other people might adopt less cautious behaviour overall. This concern appeared to be heightened by the COVID-19 context and included worries that, if other people were using the spray, they might be less likely to engage in other preventative behaviours such as masks and social distancing.

Concerns about medicines

Participants appeared to perceive RTI prevention nasal sprays as a form of medicine (the spray is officially a 'medical device'). Conceptualisation of the spray in this way seemed to persist for most participants to some degree despite encountering and understanding our intervention message that the spray is not a medicine and our comparison of regular spray use to regular hand sanitising. In line with perceiving the spray as a form of medicine, participants raised questions and concerns that are typical of medicines. For example, they were interested in ingredients and wanted to check for allergies, interactions or contra-indications with their routine medications. Participants also expressed apprehensions regarding over-use which they felt could lead to side effects, addiction or the spray becoming ineffective.

Participants often discussed trying to avoid using medicines. While this could raise concerns about using the spray, a few considered the spray a means of avoiding needing medication for RTI symptoms or disease exacerbations (e.g., antibiotics, steroids).

For peer review only

Although thinking of the spray as a medicine elicited concerns relating to medicines, thinking of the spray as something without medicine ‘status’ also appeared problematic; a minority of participants expressed slight doubt about how effective the spray could be if it was not a medicine, and not already regularly prescribed or recommended by the NHS.

Unpleasantness and hygiene

A few participants described how actions relating to noses and nasal mucous were unpleasant and socially unacceptable. A few (specifically those unfamiliar with using any type of nasal spray) found that the concept of a nasal spray inactivating and cleaning out viruses raised concerns about a messy and wet procedure. However, those who tried out the spray did not find this occurred. Given their awareness that viruses might be present in the nose, some participants were also concerned about how to use the spray hygienically. For example, they wondered whether germs left on the nozzle could infect them if they used the spray again later.

Familiarity and confidence

There was considerable variability in how much detailed information people felt they needed about exactly how to use the spray. This seemed to relate to lack of confidence and was prominent in participants who had not used any type of nasal spray before. One participant found using a spray daunting, was anxious about getting it right and found detailed instructions reassuring. Conversely, participants who had previously used another type of nasal spray appeared comfortable trying a spray and had fewer questions and concerns, seeing it as obvious and common-sense. However, this confidence could be unhelpful; one confident participant bypassed the instructions, tried the spray using the incorrect technique and experienced strong side effects. They described having thoughts about never using the spray again before realising the value of the technique instructions. Generally, people welcomed access to detailed guidance about spray technique and especially appreciated that the tips were aimed at helping them to reduce chance of side effects.

For peer review only

Reactions to possible or actual side effects

Participants considered knowing about the potential side effects of the spray important, paid keen attention to this information, but overall did not consider them off-putting. Participants stated that they would be willing to try the spray and would review their position and stop using the spray if bad side effects were experienced.

DISCUSSION

For peer review only

This paper is the first published research to explore how people think and feel about using nasal sprays, an emerging area of RTI prevention. Various important perceptions and experiences were identified which are discussed below in terms of their relevance for encouraging people to adopt and persist with this type of RTI prevention approach.

Existing theory and research

Our findings align well with expectancy-value theories of health behaviour such as Health Belief Model[43] and the Necessity-Concerns Framework[36 37]. These theories emphasise implicit cost-benefit analysis; a person adopts and perseveres with preventative health behaviours generally or adherence to a medicine specifically based upon perceived efficacy, necessity and tolerability. We found strong beliefs about necessity in both studies. Study 1 participants wanted to avoid the physical and social impact of RTIs and study 2 participants (with recurrent RTIs or vulnerabilities to severe RTIs) welcomed our information and advice and considered sprays a novel and potentially effective prevention method. Considerable interest in strategies to prevent RTIs has been recently documented in vulnerable and/or recurrent patients[32] but research with younger and/or healthy participants in non-pandemic times reveals weaker or mixed beliefs about the necessity of avoiding infections[1 2 44-48]. Both studies reported here also highlighted a range of beliefs and concerns that could plausibly reduce engagement with using nasal sprays. Concerns around discomfort and regime complexity also arose in studies about nasal irrigation and sprays for sinusitis relief[4 30]. According to expectancy-value theories, reducing

concerns and costs (alongside increasing necessity beliefs) will improve initiation and continuation of the behaviour.

A theoretical review[49] argues that medication adherence should be conceptualised as a type of causal learning and reasoning. People learn about how medications effect outcomes through a dynamic interplay of top-down (pre-existing beliefs and expectations about both specific and broad classes of treatments) and bottom-up processes (personal experiences with symptom change and side effects, particularly early in the course of treatment). This learning influences their ongoing adherence. Causal learning theory[49] predicts that learning a link between an intervention and positive outcomes (and therefore strong adherence) in the context of a nasal spray for RTI prevention could be challenging for several reasons. Firstly, people have limited data on which to reach conclusions from (e.g., several infections per year, rather than daily symptoms). Secondly, other variables confound interpretations of spray efficacy (e.g. other RTI prevention behaviours). Thirdly, the spray may not prevent infections 100% of the time, especially when use is suboptimal (timing, technique, dosage). Our findings about optimism about the spray are positive; people are likely to begin using sprays with expectancies that will facilitate interpreting a link between the spray and positive outcomes. However, some participants described doubt about effectiveness and some highlighted the difficulty of drawing strong conclusions from one's own experience. This, alongside the identified focus on side effects and concerns about using medicines, suggests that causal learning of a treatment benefit may be difficult and this may undermine adherence. An intervention to support and promote nasal spray use will need to help people form not only strong outcome expectancies but also mental models of treatment mechanisms that allow them to conclude that the spray is working (or partially working) to counteract more pessimistic or confusing conclusions that may arise if they are primarily guided by symptom and side-effect experiences.

Finally, perceived ease or difficulty of using the spray and confidence for using it were also prominent within our findings. Social Cognitive Theory highlights self-efficacy as a key predictor of behaviour[50]. Intervention complexity and lack of confidence, alongside poor adherence have also been emphasised in research on nasal irrigation for sinus symptom relief[4 30].

For peer review only

Intervention development

We undertook the two studies reported here as part of developing the Immune Defence nasal spray intervention. Study findings informed the planning of initial intervention content (study 1) and optimisation of that content (study 2). For instance, our intervention content addressed concerns about overusing medicines, side effects and hygiene as well as avoided disgust reactions. We provided persuasive information to challenge fatalism about catching RTIs, helped people to build positive expectations of the spray and to continue to hold these even if it doesn't appear to work every time. We also exploited the importance of recommendation and promoted the benefits of feeling protected. We emphasised the simplicity of spray use (and ensured a straightforward experience via clear, easy instructions) and we presented information to suit both experienced nasal spray users and less confident beginners. Supplementary Materials 6 provides further details about how this study's findings influenced our intervention content.

Strengths, limitations and future research

A key strength of this paper was its combination of findings from different samples and data collection methods allowing insights into a variety of people and experiences. Some of our data reflects experiences of people who were already motivated to buy the spray and who had some experience of using it (study 1), but we also gathered data from people for whom spray as a prevention method is clinically relevant but who did not currently use the spray (study 2). We also collected data from pre-COVID-19 times (study 1) and during the early months of the pandemic (study 2). Study 1 was a large sample but thin, brief data with little contextual information and no knowledge of reviewer demographic and clinical characteristics. Study 2 was a small but well-described sample with thicker, richer data. The think-aloud interview method of prompting participants to comment whilst reading the nasal spray rationale and instructions meant that detail was gathered about understanding and acceptability of key aspects of using the spray. Study 2 involved examination of reactions to the Immune Defence intervention content allowing insight into what is interesting, confusing, concerning, off-putting about the nasal spray as described by a specific rationale and set of instructions. Whilst some

For peer review only

of the detail is therefore particularly pertinent to the Immune Defence nasal spray intervention, the overall themes we present are generalisable to other nasal sprays and similar products, prevention behaviours, instructions and advice. Phase b of Study 2 was designed to explore how people experience beginning to use the spray for the first time. A significant limitation to this, however, is that only 7 participants took part in this phase of the research. They also tried out the spray over just three weeks, in a partial COVID-19 national lockdown and during the summer months. They therefore experienced little exposure to viruses and consequently had limited opportunity to try using the spray in the intended ways. Tracking larger numbers of participants over longer periods will provide a clearer picture of usage and adherence and will be particularly useful for shedding light on factors that may only become apparent over time (e.g. experiencing or not experiencing benefits). Qualitative and quantitative data collection on spray adherence, experiences and beliefs is currently in progress as part of the Immune Defence process evaluation.

Whilst our data suggests nasal sprays for RTI prevention are of interest to clinically higher risk subgroups of the population and considered particularly valuable in the pandemic context, whether lower risk groups (e.g. healthy adults) have similar perceptions has not been established; we have no demographic or clinical data about who the online reviews came from. Healthcare professionals are a specific group who may benefit from extra protection from RTIs and some of the recent and current research around nasal sprays and similar approaches relates to healthcare professionals at risk during procedures and provision of medical care[24]. Findings about lay people's motivations, facilitators, barriers and concerns may not transfer well to healthcare professionals; their medical expertise and the occupational setting may mean different factors are important. Additional research may therefore be needed with this group.

Conclusion

People who suffer frequent or severe infections or who are clinically vulnerable to RTIs are interested in using a nasal spray to prevent RTIs and see this as useful or even a 'game changer'. They also have some doubts and concerns and may expect to encounter (or actually encounter) certain difficulties. Many of the information needs, misunderstandings, concerns and difficulties exposed through the current

For peer review only

research may be remedied by ensuring interventions are designed to help people overcome these issues. This paper therefore exemplifies the benefit of conducting in-depth qualitative research with target users during intervention planning, development and refinement.

For peer review only

Author Contributions

LY, AG and PL conceived the study idea and initial study design with later input from LD, SW, KG, FM, JDD, KB, SRH and DS. SW led data collection with assistance from LD. SW, LD and FM led data analysis with input from all authors at different stages. LD and SW drafted the manuscript. All authors contributed to critically editing and approving the final manuscript.

Other contributors: Kate Martinson managed ethical approvals and recruitment. Thank you also to all our PPI panel members, in particular Hazel Patel, Samantha Richards-Hall and Debs Smith.

Competing interests: none declared.

Data availability statement: No additional data available.

Funding: This study/project is funded by the National Institute for Health Research (NIHR) Programme Grants for Applied Research (PGfAR) Programme. The views expressed are those of the author(s) and not necessarily those of the NIHR or the Department of Health and Social Care.

This study was nested within a NIHR Programme Grant for Applied Research: REducing Common infections in Usual practice for Recurrent Respiratory tract Infections (RECUR). (Prof Paul Little, Dr Adam Geraghty) [RP-PG-0218-20005]. Lucy Yardley is an NIHR Senior Investigator and her research programme is partly supported by NIHR Applied Research Collaboration (ARC)-West, NIHR Health Protection Research Unit (HPRU) for Behavioural Science and Evaluation, and the NIHR Southampton Biomedical Research Centre (BRC).

The research programmes of Lucy Yardley and James Dennison-Day are partly supported by the NIHR Southampton Biomedical Research Centre (BRC). The intervention development methods used for the RECUR/"Immune Defence" intervention were developed with support from the NIHR Southampton Biomedical Research Centre (BRC).

For peer review only

REFERENCES

1. Mayrhuber EA-S, Peersman W, van de Kraats N, et al. "With fever it's the real flu I would say": laypersons' perception of common cold and influenza and their differences-a qualitative study in Austria, Belgium and Croatia. *BMC infectious diseases* 2018;18(1):1-9.
2. Cedraschi C, Saya L, Klein P, et al. Representations of influenza and influenza-like illness in the community-a qualitative study. *BMC family practice* 2013;14(1):1-9.
3. McSweeney L, Rousseau N, Wilson J, et al. Stakeholders' views of recurrent sore throat, tonsillitis and their management: a qualitative interview study for the NA tional Trial of Tonsillectomy IN Adults (NATTINA Part 1). *Clinical Otolaryngology* 2017;42(2):301-06.
4. Leydon GM, McDermott L, Thomas T, et al. 'Well, it literally stops me from having a life when it's really bad': a nested qualitative interview study of patient views on the use of self-management treatments for the management of recurrent sinusitis (SNIFS trial). *BMJ open* 2017;7(11):e017130.
5. Snell LM, Wilson RP, Oeffinger KC, et al. Patient and physician explanatory models for acute bronchitis. *Journal of family practice* 2002;51(12):1035-41.
6. (ONS) OoNS. Sickness absence in the UK labour market 2020 [Available from: https://www.ons.gov.uk/employmentandlabourmarket/peopleinwork/labourproductivity/articles/sicknessabsenceinthelabourmarket/2020.
7. Finley CR, Chan DS, Garrison S, et al. What are the most common conditions in primary care?: Systematic review. *Canadian Family Physician* 2018;64(11):832-40.
8. Morbey R, Elliot A, Harcourt S, et al. Estimating the burden on general practitioner services in England from increases in respiratory disease associated with seasonal respiratory pathogen activity. *Epidemiology & Infection* 2018;146(11):1389-96.
9. Gulliford MC, Dregan A, Moore MV, et al. Continued high rates of antibiotic prescribing to adults with respiratory tract infection: survey of 568 UK general practices. *BMJ open* 2014;4(10):e006245.
10. Nowakowska M, Van Staa T, Mölter A, et al. Antibiotic choice in UK general practice: rates and drivers of potentially inappropriate antibiotic prescribing. *Journal of Antimicrobial Chemotherapy* 2019;74(11):3371-78.
11. Satia I, Cusack R, Greene JM, et al. Prevalence and contribution of respiratory viruses in the community to rates of emergency department visits and hospitalizations with respiratory tract infections, chronic obstructive pulmonary disease and asthma. *PloS one* 2020;15(2):e0228544.
12. Nam HH, Ison MG. Respiratory syncytial virus infection in adults. *bmj* 2019;366
13. Jefferson T, Del Mar CB, Dooley L, et al. Physical interventions to interrupt or reduce the spread of respiratory viruses. *Cochrane database of systematic reviews* 2020(11)
14. Saunders-Hastings P, Crispo JA, Sikora L, et al. Effectiveness of personal protective measures in reducing pandemic influenza transmission: a systematic review and meta-analysis. *Epidemics* 2017;20:1-20.
15. Lee JK, Lam GK, Shin T, et al. Efficacy and effectiveness of high-dose versus standard-dose influenza vaccination for older adults: a systematic review and meta-analysis. *Expert review of vaccines* 2018;17(5):435-43.
16. Polack FP, Thomas SJ, Kitchin N, et al. Safety and efficacy of the BNT162b2 mRNA Covid-19 vaccine. *New England Journal of Medicine* 2020
17. Voysey M, Clemens SAC, Madhi SA, et al. Safety and efficacy of the ChAdOx1 nCoV-19 vaccine (AZD1222) against SARS-CoV-2: an interim analysis of four randomised controlled trials in Brazil, South Africa, and the UK. *The Lancet* 2021;397(10269):99-111.
18. Martineau AR, Jolliffe DA, Hooper RL, et al. Vitamin D supplementation to prevent acute respiratory tract infections: systematic review and meta-analysis of individual participant data. *bmj* 2017;356

For peer review only

19. Jayawardena R, Sooriyaarachchi P, Chourdakis M, et al. Enhancing immunity in viral infections, with special emphasis on COVID-19: A review. *Diabetes & Metabolic Syndrome: Clinical Research & Reviews* 2020;14(4):367-82.
20. Grande AJ, Keogh J, Silva V, et al. Exercise versus no exercise for the occurrence, severity, and duration of acute respiratory infections. *Cochrane Database of Systematic Reviews* 2020(4)
21. Barrett B, Hayney MS, Muller D, et al. Meditation or exercise for preventing acute respiratory infection: a randomized controlled trial. *The Annals of Family Medicine* 2012;10(4):337-46.
22. Cegolon L, Javanbakht M, Mastrangelo G. Nasal disinfection for the prevention and control of COVID-19: A scoping review on potential chemo-preventive agents. *International journal of hygiene and environmental health* 2020;230:113605.
23. Kramer A, Eggers M, Hübner N-O, et al. Virucidal gargling and virucidal nasal spray. *GMS Hygiene and Infection Control* 2021;16
24. Burton MJ, Clarkson JE, Goulao B, et al. Antimicrobial mouthwashes (gargling) and nasal sprays to protect healthcare workers when undertaking aerosol-generating procedures (AGPs) on patients without suspected or confirmed COVID-19 infection. *Cochrane Database of Systematic Reviews* 2020(9)
25. Burton MJ, Clarkson JE, Goulao B, et al. Antimicrobial mouthwashes (gargling) and nasal sprays administered to patients with suspected or confirmed COVID-19 infection to improve patient outcomes and to protect healthcare workers treating them. *Cochrane Database of Systematic Reviews* 2020(9)
26. Casale M, Rinaldi V, Sabatino L, et al. Could nasal irrigation and oral rinse reduce the risk for COVID-19 infection? *International Journal of Immunopathology and Pharmacology* 2020;34:2058738420941757.
27. Radulesco T, Lechien JR, Saussez S, et al. Safety and impact of nasal lavages during viral infections such as SARS-CoV-2. *Ear, Nose & Throat Journal* 2021;100(2_suppl):188S-91S.
28. Posch W, Vosper J, Zaderer V, et al. ColdZyme Maintains Integrity in SARS-CoV-2-Infected Airway Epithelia. *Mbio* 2021;12(2):e00904-21.
29. Yardley L, Morrison L, Bradbury K, et al. The person-based approach to intervention development: application to digital health-related behavior change interventions. *Journal of medical Internet research* 2015;17(1):e4055.
30. Vennik J, Eyles C, Thomas M, et al. Management strategies for chronic rhinosinusitis: a qualitative study of GP and ENT specialist views of current practice in the UK. *BMJ open* 2018;8(12):e022643.
31. Guenezan J, Garcia M, Strasters D, et al. Povidone iodine mouthwash, gargle, and nasal spray to reduce nasopharyngeal viral load in patients with COVID-19: a randomized clinical trial. *JAMA Otolaryngology–Head & Neck Surgery* 2021;147(4):400-01.
32. Dennison L, Williamson, S., Greenwell, K., Handcock, M., Bradbury, K., Vennik, J., Yardley, L., Little, P., and Geraghty, A. W. A. Patient perceptions of vulnerability to recurrent respiratory tract infections and prevention strategies; a qualitative study.
33. Rogers RW. A protection motivation theory of fear appeals and attitude change1. *The journal of psychology* 1975;91(1):93-114.
34. Maddux JE, Rogers RW. Protection motivation and self-efficacy: A revised theory of fear appeals and attitude change. *Journal of experimental social psychology* 1983;19(5):469-79.
35. Bandura A. Health promotion by social cognitive means. *Health education & behavior* 2004;31(2):143-64.
36. Horne R, Weinman J, Hankins M. The beliefs about medicines questionnaire: the development and evaluation of a new method for assessing the cognitive representation of medication. *Psychology and health* 1999;14(1):1-24.
37. Clifford S, Barber N, Horne R. Understanding different beliefs held by adherers, unintentional nonadherers, and intentional nonadherers: application of the necessity–concerns framework. *Journal of psychosomatic research* 2008;64(1):41-46.

For peer review only

38. Leventhal H, Nerenz DR, Steele DJ. Illness representations and coping with health threats: Routledge 2020.
39. Hagger MS, Orbell S. The common sense model of illness self-regulation: A conceptual review and proposed extended model. *Health Psychology Review* 2021:1-31.
40. O'Cathain A, Croot L, Duncan E, et al. Guidance on how to develop complex interventions to improve health and healthcare. *BMJ open* 2019;9(8):e029954.
41. Skivington K, Matthews L, Simpson SA, et al. A new framework for developing and evaluating complex interventions: update of Medical Research Council guidance. *bmj* 2021;374
42. Braun V, Clarke V. Using thematic analysis in psychology. *Qualitative research in psychology* 2006;3(2):77-101.
43. Rosenstock I. Why people use health services. The Milbank Memorial Fund Quarterly, 44, 94-127, 1966.
44. Lundgren B. The Common Cold, Influenza and Immunity in Post-Pandemic Times: Lay representations of Self and Other among older people in Sweden. *Health, culture and society* 2015;8(2):46-59.
45. Yardley L, Miller S, Teasdale E, et al. Using mixed methods to design a web-based behavioural intervention to reduce transmission of colds and flu. *Journal of Health Psychology* 2011;16(2):353-64.
46. Seale H, Mak P. J., Razee, H., & McIntayre, C.(2012). Examining the knowledge, attitudes, and practices of domestic and international university students towards seasonal and pandemic influenza. *BMC Public Health*;12:307.
47. Prior L, Evans MR, Prout H. Talking about colds and flu: the lay diagnosis of two common illnesses among older British people. *Social Science & Medicine* 2011;73(6):922-28.
48. Petricek G, Hoffmann K, Vandenbroucke A, et al. Laypersons' perception of common cold and influenza prevention—a qualitative study in Austria, Belgium and Croatia. *European Journal of General Practice* 2019;25(4):220-28.
49. Rottman BM, Marcum ZA, Thorpe CT, et al. Medication adherence as a learning process: insights from cognitive psychology. *Health psychology review* 2017;11(1):17-32.
50. Bandura A. Health promotion from the perspective of social cognitive theory. *Psychology and health* 1998;13(4):623-49.

For peer review only

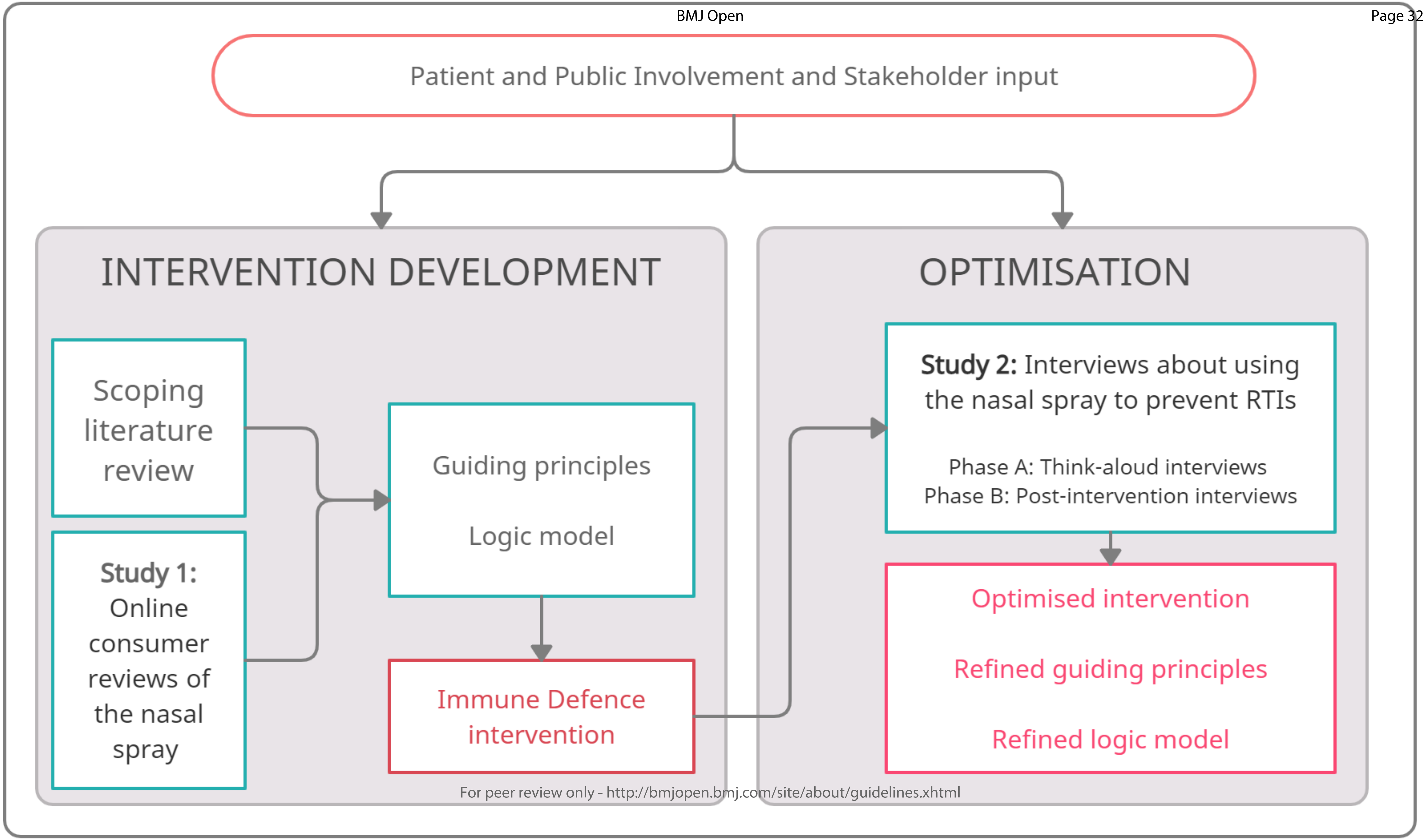
Patient and Public Involvement and Stakeholder input
INTERVENTION DEVELOPMENT
OPTIMISATION
Scoping literature review
Study 1: Online consumer reviews of the nasal spray
Guiding principles
Logic model
Immune Defence intervention
Study 2: Interviews about using the nasal spray to prevent RTIs
Phase A: Think-aloud interviews
Phase B: Post-intervention interviews
Optimised intervention
Refined guiding principles
Refined logic model

NASAL SPRAY INTERVENTION

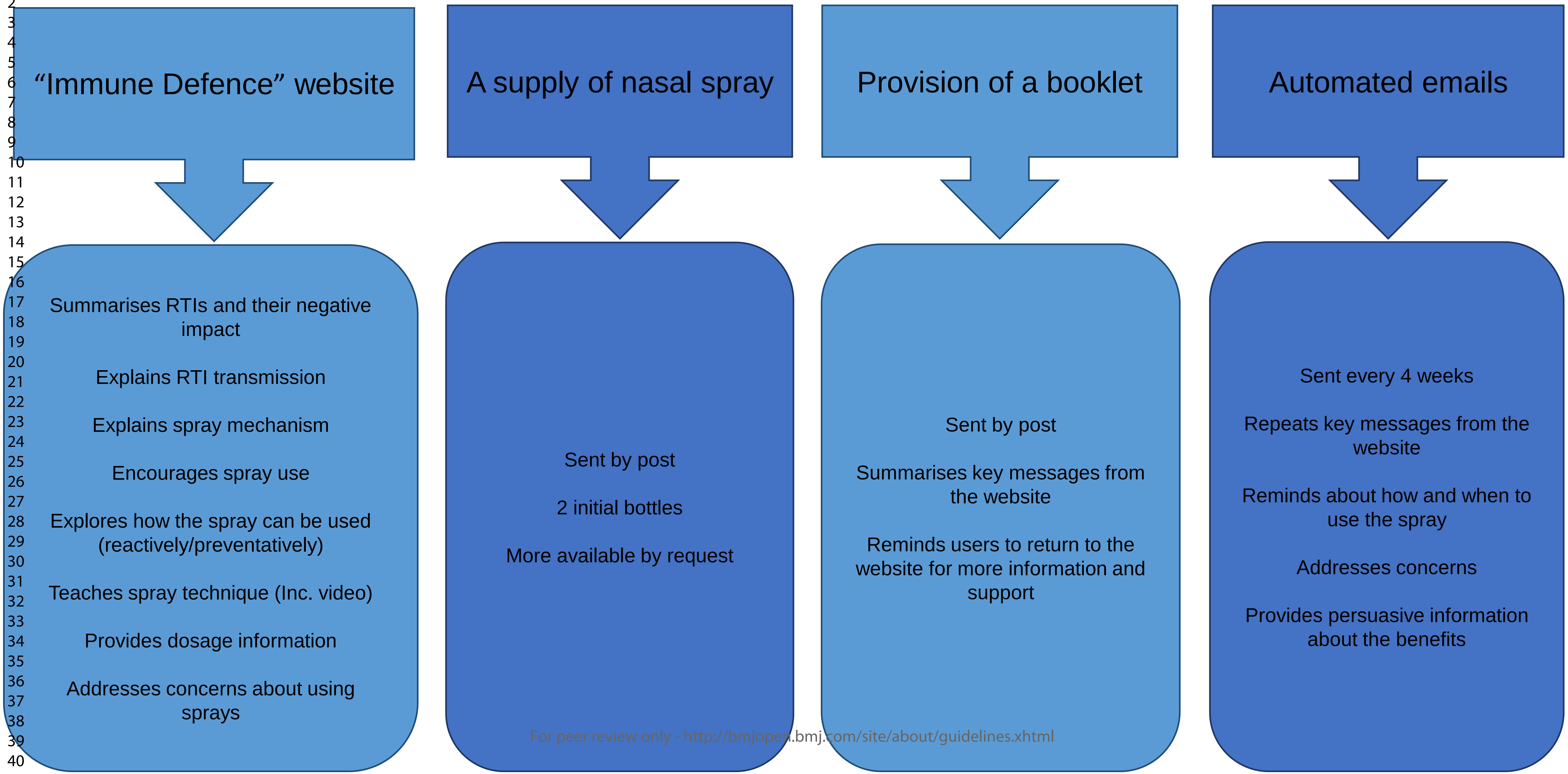

For peer review only - http://bmjopen.bmj.com/site/about/guidelines.xhtml

Supplementary material 1: RECUR (“Immune Defence”) Guiding principles – Nasal sprays

User context	Key design objective	Intervention design features
Our users have a range of nasal spray experience. Some have used sprays before, such as for hayfever, but perhaps not using the techniques we recommend for this spray. Some have never used a nasal spray before.<br><br>The idea of using sprays preventatively is a novel idea for most target users.	To support new and experienced nasal spray users to build skills to develop a correct spray technique.	• Provide an instructional video on a ‘live model’ demonstrating correct spray technique and modelling behaviour to build skills for new users.<br>• Provide an instructional booklet to be kept with the spray for ease of use and a reminder of how to use the spray correctly.<br>• Highlight specific advice which will be helpful for new and experienced sprays users (e.g. do not inhale deeply when the spray is in your nose).<br>• Advise everyone to watch the video including experienced sprays users, highlighting that this spray is administered in a different way.
Our target group perceive the spray as exciting and they are hopeful it will work. However, they may not always experience benefits when they begin using it and this could lead to disappointment and (depending on other factors, including side effects) discontinuation or suboptimal adherence. Some users also have previous experience of trying other prevention strategies with little success, leading to a fatalistic perspective of RTIs.	To support people to set and maintain positive expectations of spray efficacy, even when RTIs still occur.	• Explain the mechanism of how the spray works so that people understand that the aim of the spray is to reduce viral load not prevent viruses entering the body at all.<br>• Emphasise how sprays reduce the duration and severity of an RTI, as well as preventing them in the first place, to avoid feelings of disappointment if people do get an RTI after using the spray.<br>• Address the potential lack of efficacy beliefs by providing advice for future use (e.g. using the spray quickly at first signs of infection, using the correct technique) and motivating the user to keep trying the spray, even if they got an infection.

For peer review only

Concerns about nasal spray side effects are fairly common and may lead to discontinuation of use.	To reduce people's concerns about possible negative effects of spray use.	• Provide reassuring information to address concerns about potential side effects, specifically regarding the mildness and tolerability of the spray.<br>• Change perceptions of side effects – framing them positivity as 'normal' by describing what to expect. Explaining side effect symptoms as a sign that the spray is working effectively (e.g. getting to the areas it needs to in order to work).<br>• Compare side effects to worse outcomes if nasal spray was not used e.g. getting an infection.<br>• Provide advice and skills training on how to optimise your nasal spray technique to avoid side effects.<br>• Advise on how to cope with side effects if they did happen e.g. how to deal with a nosebleed.
People may see nasal sprays as medicines because of their mode of administration and previous experiences with sprays that are medicines (e.g. hayfever, sinus). Many people have concerns about over-use of medicines.	To help develop an alternative way of thinking about the spray, to reassure people about safety and to persuade people that nasal sprays are safe.	• Explain that nasal sprays work in a similar way to handwashing/hand gel. This provides a familiar example of something that is not a medicine but helps prevent infections. Both prevention methods are common behaviours, simple and acceptable, and they neutralise/remove germs/viruses before they can infect you and make you ill.<br>• Address the concern that the spray is a medicine by clarifying that it is not a medicine and that it is safe and non-addictive.

For peer review only

The intervention will be delivered during the COVID-19 pandemic where the threat levels and national recommendations are constantly changing.<br><br>Emerging evidence suggests that nasal sprays may be useful in providing additional protection against viruses like COVID-19, which act similarly to common RTIs but have severe consequences, particularly for vulnerable groups.<br><br>Our target group clearly spot the similarities between COVID and seasonal/normal RTIs. Those using the intervention in a pandemic context may be concerned with either or both COVID/normal RTIs.<br><br>Some of our target group have incorrect beliefs that the spray can replace government COVID recommendations or that the spray is not needed because of other behaviours mitigating the usefulness (e.g. mask wearing, social distancing).	To ensure the intervention is suitable for delivery during rapidly changing COVID-19 pandemic context.	• Be able to quickly update intervention content when needed to reflect latest guidelines and research evidence.<br>• Explain how effective the spray might be for COVID-19.<br>• Correct misconceptions about nasal sprays and COVID-19 by explaining that the spray is another layer of protection to be used with other behaviours to ensure the best protection possible again infections.

For peer review only

Supplementary material 2: Logic model

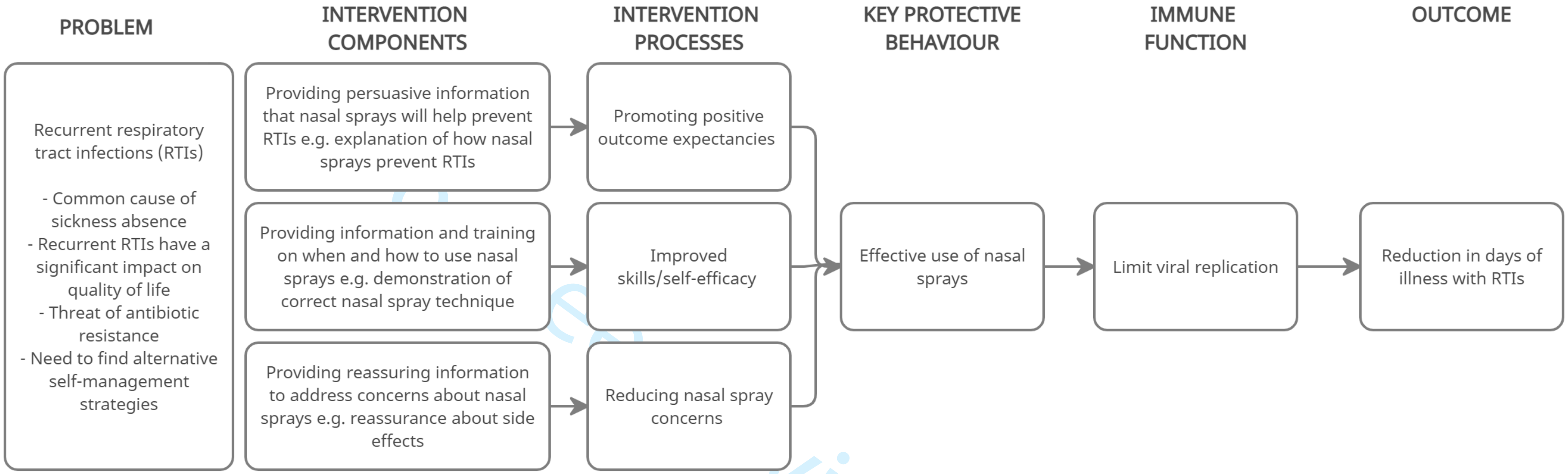

For peer review only - http://bmjopen.bmj.com/site/about/guidelines.xhtml

Supplementary material 3: Table of Changes extract

This is a simplified extract from a "table of changes" used to optimise the Immune Defence nasal spray intervention. It has been edited for clarity for readers outside of research team.

Website Section/content referred to	Original website content wording	Participant comments: positive, or likely to promote engagement/adherence	Participant comments: negative, or likely to impair engagement/adherence	Action/Changes	Final website content wording
"When do I use the spray?"<br><br>Description of first signs of an infection that should trigger use of the spray.	There are 3 ways to use the spray.<br><br>**1.When you first feel an infection coming on.**<br><br>It works best if you use it **as soon as you notice any symptoms.**<br><br>Often people say their first signs are:<br>• Sneezing;<br>• A runny nose;<br>• A tickle in the nose; or<br>• A tickle in the back of the throat	**INTERVIEWER:** So what would you say are your kind of first signs of infection? **PARTICIPANT 1:** Mine are usually sneezing and a tickle. **INTERVIEWER** Yes, so you'd be able to identify yourself in those symptoms? **PARTICIPANT 1:** Oh yes, yes.<br><br>**PARTICIPANT 3:** So I suppose when I first feel an infection coming on is when the sore throat starts. **INTERVIEWER:** So that would be your first sign of an infection happening? **PARTICIPANT 3:** That would be the warning sign, definitely.<br><br>**PARTICIPANT 4:** [participant reads website] "*Often people say the first signs are sneezing*", yes, agree with that; "*runny nose*", yes; "*tickle in the back of the nose or a tickle in the back of the throat*", yes. The other thing is a headache or feeling hot and cold - feeling hot is another sign for me anyway.<br><br>**PARTICIPANT 3**: It's great. It's absolutely everything that I and my family feel and experience.	**PARTICIPANT 7:** I think that makes sense, and you haven't said, 'These are necessarily the signs that you would get when you feel an infection coming on.' You say, 'That's what often people say they are,' so... **INTERVIEWER**: So are your first signs recognisable in there, or not? **PARTICIPANT 7:** No. I don't know. I think I tend to just feel more rundown, tired, a bit headachy. I don't know. I wouldn't say I get a runny nose at all. No, I wouldn't say they are, to be honest!<br><br>**PARTICIPANT 1:** I can't think of any other additional things that would indicate that I had a nasal infection coming on. I would perhaps personally, sometimes I get a thick throat, like the equivalent of catarrh building up… but whether that comes under a tickle, I don't know, but that's what I personally would get as an indication, like just a thickening of the mucus<br><br>**INTERVIEWER**: Would you say those first signs of infection are similar to what you experience, or is it different? **PARTICIPANT 10**: Yes.	We added a catch-all statement about a wide range of early signs of RTIs, acknowledging idiosyncrasies and building confidence in spotting own first signs.	There are 3 ways to use the spray.<br><br>**1.When you first feel an infection coming on.**<br><br>It works best if you use it **as soon as you notice any symptoms.**<br><br>Everyone's first signs of infection are different. Often people say their first signs are:<br>• Sneezing;<br>• A runny nose;<br>• A tickle in the nose;<br>• A tickle in the back of the throat;<br>• Your skin feeling sensitive;

For peer review only

			I think the first signs, they are but the first signs are often an aching, aren't they and sore? I get sore skin and aching li-, just a general ache as a first sign quite often of these bugs. So sometimes they are, but I suppose that might be the difference between colds and flu, I don't know. Sore throat I'd say, rather than a tickle in the back of the throat, but you say often…I'm not sure about these symptoms. Maybe sometimes, but not always. I think for me I often feel achy and have this very funny sensitive skin which seems a bit sore all the time, that would be the first sign, but maybe as I say, that's the difference between flu and cold, I don't know. If that's what people say then that's what most people say then that's a thing, isn't it?		• Having slightly achy muscles; or<br>• Having a mild headache.
"How does the spray work?"<br><br>Comparison of using the nasal spray to washing.<br><br>In various places the content refers to a washing/washing out metaphor and a comparison to washing hands with soap and	It's a bit like using soap when you wash your hands, only better.<br><br>The spray traps viruses and washes them out of the nose; and make the nose and throat **a very unfriendly place for viruses.**<br><br>This means it's **much harder for them to survive**	**PARTICIPANT 4** [Reading website] How does it work? *"It's a bit like using soap and wash your hands only better!*" Oh, I like that.<br><br>**INTERVIEWER:** What sounds particularly appealing about giving it a go? **PARTICIPANT 5**: It says that, well, the whole bit of how does it work? *"The spray traps the viruses and washes them out of your nose. Makes your nose and throat a very unfriendly place for the viruses. This means it's much harder for them to survive".* I'd be willing to give that a go!	**PARTICIPANT 4**: The spray traps the viruses and washes them out of the nose. Ooh, how does it wash it out of the nose? You spray it up and then it all runs down. That sounds disgusting. Do you spray it and then blow out? **INTERVIEWER**: You've got a question there about how to use it essentially, is that right? **PARTICIPANT 4**: It says here just - well, I know you know what it says but, *'Spray traps the viruses and washes them out of the nose.'* All right. Let's read the next sentence. "*Makes the nose and throat a very unfriendly place for viruses. This means it's much harder for them to*	We retained the hand cleaning metaphor but change to a comparison with hand gel rather than soap and water to provide a closer match to the spray action and avoid the procedure sounding difficulty or unpleasant. Hand gel use is currently (early	It's a bit like a hand gel, but specially designed for your nose.<br><br>The spray helps to clean the virus from your nose.<br><br>The spray also makes the nose and throat a very **unfriendly place for viruses.** This means it's much **harder for them survive** so they

For peer review

water to remove germs before they can do harm.	so they can't take hold and make you ill.	**INTERVIEWER:** Does the explanation about how it works make sense to you, about the soap and hands? **PARTICIPANT 3:** Yes, indeed. Yes, I'll say!<br><br>**INTERVIEWER:** and the explanation saying 'it's a bit like using soap when you wash your hands', does that seem to make sense to you? **PARTICIPANT 1:** Well, it does, particularly in the current situation with coronavirus!<br><br>**INTERVIEWER:** in terms of that explanation about how it works, being like soap for washing your hands, does that make sense to you, that explanation? **PARTICIPANT 7:** Yes, it does. I'm wondering if soap changes your pH levels because I've never really thought about that! It makes sense though, yes, and you said it traps viruses and washes them out of the nose, so that makes sense, I think.	*survive so they can't take hold and make you ill".* Right. Well, I'd like to know this washing out of the nose... **INTERVIEWER** Yes, because your first reaction to that was that sounds a bit gross - almost a bit disgusting. **PARTICIPANT 4**: Yes! It's like do I spray the liquid up my nose and then it all runs down my face? Or are you meant to spray it up your nose; then sniff it up; and then you swallow it? Which sounds equally disgusting I might add. I don't like that... The spray traps viruses, absolutely happy with that but washes them out of the nose has all sorts of horrible connotations.<br><br>**INTERVIEWER** You were anticipating almost that it has to come out of the nose essentially whereas this is suggesting... **PARTICIPANT 4**: Yes, exactly. On that video, all you're doing is placing the liquid spray into the nose as the barrier to the virus but there's no washing out. It's like putting some deodorant on. You put it on, and you leave it in place because it's got a job to do.	COVID pandemic) a common anti-infection product people are using with confidence.	can't take hold and make you ill.
Video – instructions how to use the spray	Part of the video demonstrates high risk situations in which you should use the nasal spray. It depicts a person being sneezed on by	-	**INTERVIEWER**: I heard you had a little bit of a giggle at one point. **PARTICIPANT 3**: [Laughs] Yes, that was just the second person sneezed all over the girl who was doing the demonstration.	No change. Content is engaging, enjoyable (and the intended message was clearly understood).	-

	somebody else (acting).				
Video – instructions how to use the spray	Part of the video demonstrates exactly how to prime the spray, insert into nostrils, spray and sniff.	**PARTICIPANT 5:** Yes, no, I think it's good. It's very informative and it's good that it gives people, like it shows people how to correctly use the spray because sometimes, the instructions on the boxes and in the packets and things, they aren't always as clear. **INTERVIEWER**: Do you think you'd find that useful yourself if you were trying out the spray for the first time? **PARTICIPANT 5:** Yes, definitely.	**INTERVIEWER:** we do have a link there to a video. You don't need to click on that. I just wanted to ask you, with the information that's here would you need to click on that link to see the video, or do you think that you'd be okay with the instructions there? **PARTICIPANT 7:** I wouldn't because obviously I've used sprays before. Somebody that hasn't used, and is a bit wary of it, would probably click on it.<br><br>**INTERVIEWER** Would you be inclined to watch the video yourself if you wanted instructions, or would you be more…? **PARTICIPANT 1**: No, to be honest, it's so straightforward using a nasal spray… I wouldn't bother with the video… Particularly at my age range, you've probably used nasal sprays several if not many times over your lifetime so you just would just use it.<br><br>**INTERVIEWER** What did you think about that video? **PARTICIPANT 4**: Yes, it's common sense really. I've been using [another] spray for years... I do keep my head straight. I do do my one or two good puffs. I don't breathe out and spray it everywhere. It's common sense.<br><br>**PARTICIPANT 2** When I first got [the spray], I used it once, even though I didn't have any need to use it and	We attempted to get more people to watch the video, by emphasising how the way of using the spray might be different.<br><br>Given that we know that incorrect use/angle can increase likelihood of side effects, and that our instructions are different to other sprays (e.g. hay fever, sinus medications) it is vital people use it as per instructions rather than according to common sense.	Click **here** to see a short video to help you master the spray technique.<br><br>This video is worth watching even if you have used nasal sprays before. The technique for **this spray** might be a bit different. Using the spray correctly give you the best chance of fighting infections!

			within seconds it had given me a headache, exactly as it did the very first time I used it about a year or so ago on the recommendation that was given to me. I felt, right, I'm not going to use it anymore. The only way I can describe the headache is it's like a freezer headache. It's exactly the same, if you take a bite of an iced lolly or something. That sort of, right between the eyes. I read about how it says that if that happens, you should aim it more towards your ear, rather than straight up…..[later in interview]……**INTERVIEWER**: Why did you decide not to watch the video? **PARTICIPANT 2:**I think I was probably rushing off to do something, or I got distracted or, no, I didn't. I think probably arrogant, I probably thought, 'Oh, for goodness sake, I don't need to be shown how to use a nasal spray.' Although, clearly I did because once I used it as recommended, I didn't get a headache.		

Readers interested in using the Person-Based Approach to intervention development and who wish to use a Table of Changes to assist the process may find resources on this website helpful: https://www.lifeguideonline.org/pba

Supplementary material 4:

Study 2 - Interview Schedules for Phase A and Phase B

Phase A: Think-aloud interviews

Prompts about key pages of intervention content [e.g. menus/ first page etc.]

- What are your first impressions of this page?
- What are you thinking now?
- What made you choose that option?
- What do you think about [this activity, this information, this strategy/tool/idea]?
- Can you tell me a bit more about why you think that?
- [in response to an expression of like/dislike] What is it you like/don't like about that?
- That's really interesting…..
- [picking up on vocalisations/tone of voice etc] I noticed that you paused/groaned/laughed/sighed etc. at……… Can you tell me what you thought about that?

After working through the key pages of intervention content:

- Overall, what do you think about the web pages?
- Can you tell me about anything you thought was particularly good about the web pages?
- Can you tell me anything about the web pages that you were less keen on?
- Which parts did you find most relevant to you? Which parts were the least relevant to you?
- Having looked at the web pages, can you tell me how you feel about trying to use a nasal spray to try to reduce these sorts of infections
- How much of what you've seen today do you think is relevant to coronavirus?*
- How at risk do you feel about getting these infections at the moment?
- What do you feel about the recommendation to use the spray when at high risk and how this applies to coronavirus?*
- What device did you use to look at the website today?
    - If you were using the website over a longer period of time, how would you access the website?
    - Would you use mobile phone at all?

For peer review only

Phase B: Post-intervention interviews

- Can you start by telling me overall how you got on with trying the spray?

Questions if they have tried the spray:

- Can you tell me all about how you found using the nasal spray?
- Can you tell me about anything you liked or found easy?
- Can you tell me about anything you disliked or found difficult?
    - Can you let me know if there was anything you found helped with that?
    - Can you tell me about anything that worried you about using the spray?
- Can you tell me about when you used the nasal spray?
    - When did you think to use it?
    - Can you tell me about whether any situations came up where you could have used the spray (e.g. first symptoms, feeling a risk of catching an infection)? Can you tell about how you decided whether to use the spray?
- Can you tell me about what you thought were the advantages of using the nasal spray?
- Can you tell me about what you thought were the disadvantages of using the nasal spray?
- Can you tell me what it's been like for you trying these activities/changes whilst in (partial) lockdown because of coronavirus/COVID-19*?
    - Explore the context – have they been self-isolating? Shielding?
    - What have your infections been like during this time? (More/less?)
    - What aspects of lockdown have made it easier to try these activities/manage your infections?
    - What aspects of lockdown have made it harder to try these activities/manage your infections?
    - Can you tell me about any information or advice that was difficult for you to follow during lockdown?

Questions if they have not managed to try the spray:

- Can you tell about what you thought about the idea of using a spray to try to prevent infections?
- Can you tell me about anything about the spray that you liked or found easy?
- Can you tell about anything about the spray that seemed off-putting or difficult for you?
- Can you tell me about anything that worried you about using the spray?
- Can you tell me anything you feel would help you in the future with trying the spray?
- Can you tell me about whether any situations came up where you could have used the spray (e.g. first symptoms, feeling a risk of catching an infection)?
- Can you tell about how you decided whether to use the spray?
- Can you tell me about what you thought were the advantages of using the nasal spray?

For peer review only

- Can you tell me about what you thought were the disadvantages of using the nasal spray?
- Can you tell me what it's been like for you trying these activities/changes whilst in (partial) lockdown because of coronavirus/COVID-19?*
    - Explore the context – have they been self-isolating? Shielding?
    - What have your infections been like during this time? (More/less?)
    - What aspects of lockdown have made it easier to try these activities/manage your infections?
    - What aspects of lockdown have made it harder to try these activities/manage your infections?
    - Can you tell me about any information or advice that was difficult for you to follow during lockdown?

Website questions:

- What did you think of website that gave you information and advice about using the nasal spray?
- Can you tell me about anything that you liked about the website?
- Can you tell me about anything that you disliked?
- Can you tell me about anything that you would change in the website?
- Can you tell me about anything that you thought was particularly relevant to you?
- Can you tell me about anything that you thought was not particularly relevant to you?
- Can you tell me about any information or advice that didn't make sense?
- How do you think that could be changed?
- Could you tell me about anything that you thought didn't work properly?
- Can you tell me about whether you went onto the website more than once? (explore why they returned/whether they found what they needed).
- Since looking at the website, how do you feel about infections now?

Spray Instructions:

- On the website, it mentions 3 situations where you should use the spray. What did you think about these instructions?
- On the website, it mentions how often to use the spray in each of these 3 situations. What did you think about these instructions? (prompting around the instructions).
- On the website, there is a video about how to use the spray. What did you think about this?
- Can you tell me what you thought about the paper booklet about the spray? (repeat questions above as necessary- liked, disliked etc).

Open-ended Questions about personal experiences of RTIs:

1. Can you tell me all about your experience of these sorts of infections [repeat list of RTIs if necessary: colds, flu, coughs, chest infections, bronchitis, ear infections, sinusitis, sore throats, throat infections and tonsillitis].

For peer review only

- Prompts:
  - Can you tell me about the types of illnesses you tend to get?
  - Can you tell me about when you tend to get these illnesses?
  - What's it like for you when you have them?
  - How often do you get them?
  - How long do they last?

2. Can you tell me about why you think you get these sorts of illnesses?
   - Prompts:
     - Can you tell me about what you think the causes of these illness?
     - Any other reasons why you think you get them?
3. Can you tell me about things you do to try and stop getting these illnesses?
   - Prompts:
     - What made you decide to use these things? Why is it important for you to x/y/z? (e.g. eat healthy, exercise, get the flu jab)
     - How helpful do you find these things?
     - Why do you think they work?
4. When you have these sorts of illnesses is there anything you do to try and make it go away quicker?
   - Prompts
     - Any things you take, or things you do, or avoid doing?
     - What made you decide to use these things? Why is it important for you to x/y/z?
     - How helpful do you find these things?
     - Why do you think they work?

[*coronavirus question and probing was not in the original interview schedule and was added in for later interviews]

For peer review only

<u>Supplementary material 5: COREQ checklist</u>

Tong et al 2007, 32 item checklist

https://academic.oup.com/intqhc/article/19/6/349/1791966

		**Item**	**Guide questions/description**	**Manuscript section where information can be found**
**Domain 1: Research team and reflexivity**				
Personal Characteristics	1	Interviewer/facilitator	Which author/s conducted the interview or focus group?	Method- data collection - Phase B: Post-intervention interviews
	2	Credentials	What was their occupation at the time of the study?	Method- data collection - Phase B: Post-intervention interviews
	3	Occupation	What was their occupation at the time of the study?	Method- data collection - Phase B: Post-intervention interviews
	4	Gender	Was the researcher male or female?	Method- data collection - Phase B: Post-intervention interviews
	5	Experience and training	What experience or training did the researcher have?	Method- data collection - Phase B: Post-intervention interviews
Relationship with participants	6	Relationship established	Was a relationship established prior to study commencement?	Method- recruitment
	7	Participant knowledge of the interviewer	What did the participants know about the researcher? e.*g. personal goals, reasons for doing the research*	Method- recruitment
	8	Interviewer characteristics	What characteristics were reported about the interviewer/facilitator? e.g. *Bias, assumptions, reasons and interests in the research topic*	Method- data collection<br>Discussion<br>Funding statement
**Domain 2: study design**				

For peer review only

Theoretical framework	9	Methodological orientation and Theory	What methodological orientation was stated to underpin the study? *e.g. grounded theory, discourse analysis, ethnography, phenomenology, content analysis*	Method- data analysis
Participant selection	10	Sampling	How were participants selected? *e.g. purposive, convenience, consecutive, snowball*	Method- recruitment
	11	Method of approach	How were participants approached? e.*g. face-to-face, telephone, mail, email*	Method- recruitment
	12	Sample size	How many participants were in the study?	Method- recruitment
	13	Non-participation	How many people refused to participate or dropped out? Reasons?	n/a<br><br>Not reported in main manuscript for conciseness.<br><br>Our recruitment method does not allow us to know why participants did not respond to our invitation to participate.
	14.	Setting of data collection	Where was the data collected? e.*g. home, clinic, workplace*	Method- data collection
	15.	Presence of non-participants	Was anyone else present besides the participants and researchers?	Not reported in main manuscript for conciseness.<br><br>Participants were asked to be in a quiet room with no interruptions but we do not know for sure if it was always possible as most interviews were via telephone. Field notes and

For peer review only

				interview recordings from one face-to-face interview suggest a spouse was present and commenting occasionally.
	16.	Description of sample	What are the important characteristics of the sample? *e.g. demographic data, date*	Results- participants & Table 2
Data collection	17.	Interview guide	Were questions, prompts, guides provided by the authors? Was it pilot tested?	Method- PPI section, data collection - post-intervention interviews, Supplementary material 4
	18.	Repeat interviews	Were repeat interviews carried out? If yes, how many?	Method- data collection - post-intervention interviews, Table 2.
	19.	Audio/visual recording	Did the research use audio or visual recording to collect the data?	Method- data collection - post-intervention interviews
	20	Field notes	Were field notes made during and/or after the interview or focus group?	Methods- data collection – post-intervention interviews
	21.	Duration	What was the duration of the interviews or focus group?	Methods- data collection - post-intervention interviews
	22.	Data saturation	Was data saturation discussed?	Not reported in main manuscript for conciseness.<br><br>The authors are very cautious about claims of data saturation.<br>Saturation for the current analysis was not aimed for but may have been achieved or approached.<br><br>Recruitment stopped when iterative intervention development was concluded i.e. the research team were satisfied that the interventions were

For peer review only

				as engaging as possible and a range of different viewpoints from patients with different clinical and demographic characteristics had been heard.
	23.	Transcripts returned	Were transcripts returned to participants for comment and/or correction?	n/a<br><br>(member checks with participants were not conducted, professional transcribers transcribed the interviews and researchers checked for accuracy)
**Domain 3: analysis and findings**				
Data analysis	24.	Number of data coders	How many data coders coded the data?	Method- data analysis - post-intervention interviews.
	25.	Description of the coding tree	Did authors provide a description of the coding tree?	Method- data analysis - post-intervention interviews. A coding tree was not used. We present a description of our process of inductive thematic analysis.
	26.	Derivation of themes	Were themes identified in advance or derived from the data?	Method- data analysis - post-intervention interviews.
	27.	Software	What software, if applicable, was used to manage the data?	Method- data analysis - post-intervention interviews.
	28.	Participant checking	Did participants provide feedback on the findings?	n/a<br><br>(member checks were not conducted)
Reporting	29.	Quotations presented	Were participant quotations presented to illustrate the themes / findings? Was each quotation identified? e.*g. participant number*	Findings

For peer review only

	30.	Data and findings consistent	Was there consistency between the data presented and the findings?	Findings
	31.	Clarity of major themes	Were major themes clearly presented in the findings?	Findings
	32.	Clarity of minor themes	Is there a description of diverse cases or discussion of minor themes?	Findings

For peer review only

Supplementary material 6: Themes and how they were used for intervention development

**Study findings**<br><br>**Theme title (and study where it was identified)**	**Summary of theme/finding**	**"Immune Defence" nasal spray Intervention component/content**<br>(NB previous research, theory, stakeholder opinions also fed into these decisions alongside Study 1 and 2 findings)
Motivation to avoid infections (study 1)<br><br>Excitement and optimism about a novel prevention method (study 2)	High motivation to avoid RTI (for a range of health, work, social reasons)<br><br>Explanation of spray mechanism and ways of using generated interest, hope, willingness to try.	These findings, in conjunction with our target group characteristics (recurrent RTIs/vulnerable to RTIs) meant we decided to not include significant content to convince of the necessity of avoiding infections. We kept content about the impact of RTIs and necessity of avoiding them brief and used this section predominantly to show empathy, establish a connection with users and help convince them that the intervention was relevant to them.
Inevitability (study 1)	Beliefs/experiences that RTIs are inevitable and can't be prevented or course altered once they have begun	Acknowledge current feeling and experiences of lack of control/inevitability but then build a convincing rationale for how the spray provides a chance to prevent/avoid RTIS. Describe a novel, interesting, plausible mechanism that people can understand as working in a different way to current/past prevention strategies they may have tried and experienced as ineffective.
Alternative approaches to infection prevention (study 1)	Belief that other approaches are (more) helpful for preventing RTIs	Do not attempt to persuade that any specific existing behaviours/habits/prevention methods are unhelpful/unnecessary, but refer to overall experience of wanting to gain more control and protection from infections.<br><br>Position the spray as an extra protection measure (along with novelty message and convincing rationale about how it works).
Recommendations from others (study 1)	Other people's recommendations are important	Provide a strong message of recommendation. This is given authority by NHS, University involvement and 'meet the team' of experts page and reference to scientific research.

For peer review only

Protection from risky situations (study 1)<br><br>Considering use in risky situations (study 2)	Interest in using spray to protect oneself from RTIs in situations perceived to be high risk<br><br>Considerable interest in using spray to protect oneself from RTIs n situations perceived to be high risk, especially during COVID-19 pandemic.<br><br>Some ability to correctly identify high risk situations but also some difficulty/uncertainty, especially in the context of the COVID-19 pandemic and its restrictions/mitigations.	Provide a positive message about being able to take steps to protect yourself.<br><br>Help users identify high risk situations to use the spray in. Provide examples of when to use the spray (using some examples that study participants spontaneously came up with – e.g. Public transport, aeroplanes, childcare/grandchildren situations).<br><br>Use follow-up intervention emails to revisit/remind about the types of situations and the ability of the spray to work in addition to existing mitigations. We had to edit intervention emails in real time to ensure situations and examples are well aligned with pandemic risk levels, lockdowns, restrictions.<br><br>Description of the spray as an extra layer of protection in addition to existing mitigations (e.g. Face coverings, handwashing).
Ease or difficulty (study 1)<br><br>Familiarity, confidence and information needs (study 2)	Participants vary in how easy or difficult they find using the spray. Overall, it is easy but some aspects of it require attention for best results.<br><br>Depending, in part, on past experiences of nasal sprays people may be under or over-confident in using the spray. This could lead to either anxiety about using the spray or failing to follow the instructions.	Persuasion (text) and demonstration (video) that the spray use is easy, quick and convenient.<br><br>Acknowledgment that it may take more than one use to perfect the technique (e.g. "After a few tries you will work out what feels comfortable for you", "you'll soon get the hang of it").<br><br>Clear instructions to ensure that it is experienced as easy and identified uncertainties and concerns are eliminated.<br><br>Short instructions, supplemented by optional more detail (website: drop down sections; booklet=short infographic style instructions plus reference to website for further information)<br><br>Persuasive text to stop people skipping important information by highlighting why it is useful/new (e.g. "check out this video to see how to use your nasal spray. This video is worth watching even if you have used nasal sprays before. The technique for **this spray**

For peer review only

		might be a bit different. Using the spray correctly give you the best chance of fighting infections!")<br><br>Inclusion of a suggestion to try the spray out when it arrives to boost confidence prior to time it is needed.
Experiencing side effects (study 1)<br><br>Reactions to possible/actual side effects (study 2)	Side effects of the spray are common, milder ones are tolerated if benefits are expected/experienced. Strong side effects can prevent further use.<br><br>Reassuring information about side effects is valued. People describe being willing to try the spray despite minor side effects. Severe side effects seem likely to influence discontinuation.	Explanation that side effects are minor<br><br>Framing of sensations in nose and throat (e.g. tingling, noticeable taste) as normal and a positive sign the spray is working/reaching the right place rather than a side effect.<br><br>Comparisons of mild side effects with more severe and prolonged symptoms of 'full blown infection'.<br><br>Instruction and demonstration on how to avoid the more severe side effects (spray technique).<br><br>Instructions on how to cope with side effects (e.g. position to adopt for nose bleeds, use of saline solution for dry/irritated nose, eating/drinking to eliminate unpleasant taste).
Identifying early signs of infection (Study 2)	Participants often but not always have awareness of first signs of infection and confidence in being able to use the spray in response	Give sufficient information about which first signs are relevant by listing main signs that people recognise as relevant to RTIs (feeling in throat, malaise) but also allowing for idiosyncratic first signs.<br><br>Acknowledge the difficulty distinguishing some symptoms (e.g. Runny nose, sneezing - hayfever & RTI overlap).<br><br>Explain and reassure that it would be advisable and safe to use on a symptom that turned out not to be an RTI symptom.<br><br>Given that we know people may miss first signs, refer to failure to act quickly enough as a possible explanation for situations where

For peer review only

		the spray does not appear to have helped (to try to prevent users from concluding that the spray is ineffective).
Expectations and experiences of success and failure (study 1)<br><br>Excitement and Optimism about a novel prevention method (study 2)	Users experience combinations of success and failure with the spray which then influence the continuation of use.<br><br>Idea of spray elicits interest, hope, willingness to try. For some this is very pronounced, for others it is more muted or sceptical.	Promote excitement, interest, positivity and expectations of success (via convincing explanation of how it works)<br><br>Provide a rationale for how spray use might lead to partial success (not avoiding infections but having shorter and less severe).<br><br>Provide a rationale for why it might not always work (using too late)<br><br>Encourage persistence if it does not appear to work, including attributing failure to using it too late and explaining that it may nonetheless have reduced infection severity and duration.<br><br>Encourage formation of the spray as a low risk / safe / easy intervention suitable for regular use in order to help them to conclude that potential for benefit outweighs concerns, even if they do not experience clear cut evidence of success (i.e. It is like a hand gel, not a medicine, and with no serious side effects)
Excitement and optimism about a novel prevention method (study 2)<br><br>Consequences of feeling protected (study 2)	Idea of spray elicits interest, hope, willingness to try. For some this is very pronounced, for others it is more muted or sceptical.<br><br>Feeling protected may make people feel safer, more confident and more able to participate in valued activities. It could also make people take more risks.	Boost positive expectancies of the spray's psychosocial effects including how the spray can make you feel more confident and in control and that it feels good to feel more protected [emails]<br><br>Alongside positive expectations of spray efficacy, promote continued adherence to COVID-19 regulations/guidance/mitigations; positioning the spray as an additional, not a replacement behaviour.<br><br>Do not refer to or recommend against any current infection control behaviours (e.g. Keeping distance from ill people, good respiratory hygiene, good diet, being physically fit)
Concern about medicines (Study 2)	People see nasal sprays as a medicine, eliciting medication-related	Explicitly position the spray as not a medicine, whilst maintaining expectations that it will be a powerful and effective product.

For peer review only

	concerns such as overuse, allergies, contraindications	Compare it instead to hand gel (noting the similar mechanisms- cleaning away virus before it can cause illness).<br><br>Provide reassuring information about how often it is safe to use it and how it can be used with any prescription and over-the-counter medications.<br><br>Position spray as a means of avoiding using medications such as antibiotics, over-the-counter cold relief.<br><br>NB- we expect concerns related to medicines to persist to some degree in some participants despite our 'not a medicine' message. The spray might, to a layperson, feel like a medicine in terms of its mode of administration and anticipated efficacy. Our content nonetheless promotes beliefs about it being a simple, safe and effective intervention.
Disgust and hygiene (Study 2)	Noses and nasal sprays can be considered disgusting and/or messy and unhygienic	Reassurance that the spray procedure is not wet, messy or unpleasant.<br><br>This required a change (between study 2a and study 2b) from our original description of the spray being not like a medicine but like washing hands with soap and water. We adopted a neater/cleaner explanation (like hand gel). The public were becoming very familiar with hand gel as an important infection control product at this point in the COVID-19 pandemic.<br><br>Emphasise easiness of using the spray.<br><br>Instructions on how to use hygienically.

For peer review only

Using nasal sprays to prevent respiratory tract infections: A QUALITATIVE STUDY OF ONLINE CONSUMER REVIEWS AND PRIMARY CARE PATIENT INTERVIEWS

Journal:	*BMJ Open*
Manuscript ID	bmjopen-2021-059661.R1
Article Type:	Original research
Date Submitted by the Author:	26-May-2022
Complete List of Authors:	Williamson, Sian; University of Southampton, School of Psychology<br>Dennison, Laura; University of Southampton School of Psychology, Psychology<br>Greenwell, Kate; University of Southampton School of Psychology,<br>Denison-Day, James; University of Southampton<br>Mowbray, Fiona; University of Southampton School of Psychology,<br>Richards-Hall, Samantha; No institution, Patient and Public Contributor<br>Smith, Deb; No institution, Patient and Public Contributor<br>Bradbury, Katherine; University of Southampton, Psychology<br>Ainsworth, Ben; University of Bath, Department of Psychology; University of Southampton<br>Little, Paul; University of Southampton, Primary Care and Population Science;<br>Geraghty, Adam; University of Southampton, Primary Care and Population Sciences<br>Yardley, Lucy; University of Southampton, Psychology; University of Bristol, School of Health Sciences
**Primary Subject Heading**:	Respiratory medicine
Secondary Subject Heading:	Public health, Qualitative research
Keywords:	QUALITATIVE RESEARCH, Respiratory infections < THORACIC MEDICINE, PREVENTIVE MEDICINE

SCHOLARONE™
Manuscripts

BMJ

I, the Submitting Author has the right to grant and does grant on behalf of all authors of the Work (as defined in the below author licence), an exclusive licence and/or a non-exclusive licence for contributions from authors who are: i) UK Crown employees; ii) where BMJ has agreed a CC-BY licence shall apply, and/or iii) in accordance with the terms applicable for US Federal Government officers or employees acting as part of their official duties; on a worldwide, perpetual, irrevocable, royalty-free basis to BMJ Publishing Group Ltd ("BMJ") its licensees and where the relevant Journal is co-owned by BMJ to the co-owners of the Journal, to publish the Work in this journal and any other BMJ products and to exploit all rights, as set out in our licence.

The Submitting Author accepts and understands that any supply made under these terms is made by BMJ to the Submitting Author unless you are acting as an employee on behalf of your employer or a postgraduate student of an affiliated institution which is paying any applicable article publishing charge ("APC") for Open Access articles. Where the Submitting Author wishes to make the Work available on an Open Access basis (and intends to pay the relevant APC), the terms of reuse of such Open Access shall be governed by a Creative Commons licence – details of these licences and which Creative Commons licence will apply to this Work are set out in our licence referred to above.

Other than as permitted in any relevant BMJ Author's Self Archiving Policies, I confirm this Work has not been accepted for publication elsewhere, is not being considered for publication elsewhere and does not duplicate material already published. I confirm all authors consent to publication of this Work and authorise the granting of this licence.

For peer review only

USING NASAL SPRAYS TO PREVENT RESPIRATORY TRACT INFECTIONS: A QUALITATIVE STUDY OF ONLINE CONSUMER REVIEWS AND PRIMARY CARE PATIENT INTERVIEWS

Sian Williamson*[1], Laura Dennison*[1], Kate Greenwell[1], James Dennison-Day[1],, Fiona Mowbray[1], Samantha Richards-Hall[5]; Debs Smith[5], Katherine Bradbury[1,4], Ben Ainsworth[6], Paul Little[2] and Adam W A Geraghty[2] Lucy Yardley[1,3]

[1]Centre for Clinical and Community Applications of Health Psychology, Faculty of Environmental and Life Sciences, University of Southampton, Southampton, UK; [2]School of Primary Care, Population Sciences and Medical Education, Faculty of Medicine, University of Southampton, Southampton, UK; [3]School of Psychological Science, University of Bristol, Bristol, UK. [4]NIHR Applied Research Collaboration (ARC) Wessex, Southampton, UK. [5] No affiliation (Patient and Public Contributors), [6] Department of Psychology, University of Bath.

*Joint First Authors

Corresponding Author: Laura Dennison, School of Psychology, University of Southampton, Highfield Campus, Southampton, SO17 1BJ, l.k.dennison@soton.ac.uk

Word count (excluding title page, abstract, references, figures and tables)=4,569

Keywords: Respiratory Tract Infections, Common Cold, Influenza, Prevention, Qualitative Research, Interviews, Thematic Analysis

For peer review only

ABSTRACT

Objectives: Nasal sprays could be a promising approach to preventing Respiratory Tract Infections (RTIs). This study explored lay people's perceptions and experiences of using nasal sprays to prevent RTIs to identify barriers and facilitators to their adoption and continued use.

Design: Qualitative research. Study 1 thematically analysed online consumer reviews of a RTI prevention nasal spray. Study 2 interviewed patients about their reactions to and experiences of a digital intervention that promotes and supports nasal spray use for RTI prevention (reactively: at 'first signs' of infection and preventatively: following possible/probable exposure to infection). Interview transcripts were analysed using thematic analysis.

Setting: Primary Care, UK.

Participants: 407 online customer reviews. 13 purposively recruited primary care patients who had experienced recurrent infections and/or had risk factors for severe infections.

Results: Both studies identified various factors that might influence nasal spray use including: high motivation to avoid RTIs, particularly during the COVID-19 pandemic; fatalistic views about RTIs; beliefs about alternative prevention methods; the importance of personal recommendation; perceived complexity and familiarity of nasal sprays; personal experiences of spray success or failure; tolerable and off-putting side effects; concerns about medicines; and the nose as unpleasant and unhygienic.

Conclusions: People who suffer disruptive, frequent or severe RTIs or who are vulnerable to RTIs are interested in using a nasal spray for prevention. They also have doubts and concerns and may encounter problems. Some of these may be reduced or eliminated by providing nasal sprays users with information and advice that addresses these concerns or helps people overcome difficulties.

For peer review only

ARTICLE SUMMARY: STRENGTHS AND LIMITATIONS OF THIS STUDY

- This is the first research about how people think and feel about using a nasal spray to prevent respiratory tract infections (RTIs) so adopting an exploratory, inductive, qualitative approach allowed insight into key issues.

- The paper benefits from its coverage of multiple *populations*, *data collection approaches* and *contexts.*

- The pandemic context, short study period and season meant Study 2 participants had little exposure to viruses and limited opportunities to try out their sprays.

- The study 2 sample lacked ethnic diversity, tended to have low levels of deprivation, and consisted of more females than men.

- This paper demonstrates the benefit of conducting in-depth qualitative research with target users during intervention planning, development and refinement.

For peer review only

INTRODUCTION

Respiratory tract infections (RTIs) such as the common cold, influenza, bronchitis, tonsillitis and sinusitis are commonly experienced by most adults. Although they tend to be self-limiting, these illnesses are disruptive and unpleasant[1-5], cause substantial workplace sickness absence[6] and contribute significantly to pressures on primary care[7 8]. Consultations for RTIs also result in unnecessary antibiotics prescriptions, thus contributing to antibiotic resistance[9 10].

Typical RTI prevention approaches reduce the likelihood of becoming infected, (e.g., social distancing[11], face-coverings[11] and handwashing[11 12]) or improve individuals' immune responses (e.g.vaccination[13-15], nutrition[16 17], physical activity[18 19]). Prevention approaches can also intervene at early stages of infection by targeting the nose and the mouth as entry points for viruses [20]. These approaches include using mouthwashes and rinses and nasal sprays, douches, and irrigation. Products may be used regularly and/or in responsible to possible/probable exposure. The mechanism of action appears to be mechanical; either forming a barrier or having a washing out action. These products may also alter the environment of the nose and/or throat, reducing the viral load and the chance the virus will survive/thrive [20 21]. The COVID-19 pandemic has prompted a resurgence of interest in these approaches[20 22-27]. Many formulations and products are under investigation, with some promising findings. For example, a systematic review concluded that iota-carrageenan nasal sprays have a good safety profile and powerful antiviral activity against the common cold[21]. A series of recent reviews and commentaries conclude that these approaches should be subject to further evaluation and/or rapid rollout in the COVID-19 pandemic. Various randomised-controlled-trials are ongoing. The RECUR trial (ICTRN17936080) evaluates preventative use of nasal sprays to reduce the frequency, duration and severity of non-pandemic RTIs in recurrent and at-risk primary care patients whilst the ICE-COVID trial[24] evaluates throat and nasal sprays for COVID-19 prevention in healthcare professionals (HCPs).

Along with evidence about efficacy, it is also essential to accrue evidence about the acceptability of these approaches for the people who may eventually be encouraged to adopt them. Kramer and colleagues[20] describe nasal rinsing as "easily

For peer review only

implementable" as a COVID-19 public health measure. However, lay people/patients may not find these approaches easy or acceptable[28].

No published research has investigated views or experiences of using these approaches for preventing RTIs. However, research exists on similar approaches when used for symptom relief. People with chronic rhinosinusitis describe difficulties using steroid nasal sprays including forgetting to use them, and lack of confidence with technique[29]. It may be considered awkward, prohibitively time-consuming[29] and uncomfortable, and, consequently, patients may use these methods irregularly, stopping once relief is gained[4]. Together, these studies indicate that RTI prevention strategies requiring nasal application of a substance may be off-putting for some patients and regular, long-term persistence may be problematic. Identifying concerns and difficulties (along with more positive beliefs and experiences) would allow patient education to be tailored to include persuasive messages and appropriate support to help people overcome barriers.

This paper extends the literature by investigating people's perceptions and experiences of using a nasal spray for preventing RTIs. We report findings from two qualitative studies. The first is an analysis of online customer reviews of a RTI prevention nasal spray. The second study analyses interviews with patients heavily burdened by and/or at higher risk from RTIs about their perceptions of and experiences of using a nasal spray for RTI prevention. Our aim for both studies was to explore how people think and feel about using nasal sprays to prevent RTIs and to identify barriers and facilitators to the adoption and continued use of sprays. If sprays prove effective in trials, it is important to have a behavioural evidence base to guide interventions that support optimal use. The findings will be valuable to researchers and clinicians seeking to develop or implement RTI prevention approaches, especially those involving nasal sprays or similar prophylactic products such as nasal and mouth rinses and washes.

For peer review only

METHOD

Intervention development context

The studies reported in this paper were undertaken as part of the development of a digital behavioural intervention to encourage and support people to use a nasal spray to prevent RTIs (NIHR programme grant RP-PG-0218-20005; 'RECUR'). A randomised-controlled-trial is currently evaluating the efficacy of the nasal spray intervention; within the trial the brand name of the spray is masked. Therefore, this paper simply refers to it as 'the nasal spray'. As a regulated medical device, the safety of the spray has been established. It is available to purchase in the UK and currently retails under £10. The manufacturer instructions advise use at the first signs of a cold. In the intervention under evaluation, participants are also advised to use the spray at first signs of *any* suspected RTI and also in situations where exposure to RTIs is likely (e.g., crowded places, close proximity to infected people).

The intervention development work used the person-based approach[28], which prioritises in-depth qualitative data collection to explore the views and experiences of potential intervention users, in order to understand the context in which users engage with interventions and behaviour change. Figure 1 shows how the studies reported here were used alongside primary qualitative research[30], a scoping review, behaviour change theory (Protection Motivation Theory[31 32], Social Cognitive Theory[33], Necessity-Concerns Framework[34 35], Sense Model[36 37]) and stakeholder and PPI involvement to develop and optimise the intervention. The two studies reported here influenced the development of 'guiding principles'[28] (Supplementary Materials 1) and the articulation of programme theory through a logic model for the intervention[38 39] (Supplementary Materials 2), then enabled iterative changes to the intervention (Supplementary Materials 3).

Figure 1: overview of nasal spray intervention development activities.

[INSERT FIGURE 1 HERE]

For peer review only

Study 1: online consumer reviews of the nasal spray

Data collection

407 customer reviews of the nasal spray were taken from four large, commercial websites (comprising 263, 93, 30 and 21 spray reviews each). The websites were selected based upon having a large number of spray reviews. All reviews were included (positive, negative) except those which focused on supplier-based issues (e.g. damaged product). We also removed reviews that were duplicated across websites. The search for reviews was conducted in August 2019.

Analysis

We used an inductive thematic analysis approach. Although the review data was 'thin' and brief (typically several sentences for each review) we selected this approach to remain open and explorative and to generate broad themes that summarised important topics. Coding was undertaken by SW and FM who separately coded half of the reviews each in NVivo12 and then worked together to review, combine, discuss and refine coding. They then developed preliminary descriptive themes to capture key issues within the data. These were subsequently inspected, reorganised and relabelled by LD and SW.

Study 2: Interviews about using a nasal spray to prevent RTIs

Recruitment

We sought participants who experience frequent or recurrent infections and/or who are at risk of more severe RTIs. Three UK GP practices identified possible participants by searching their patient lists and posting invitations and information sheets to patients who consulted for ≥1 RTI within the last year and were prescribed antibiotics. They also wrote to patients who had asthma, COPD or chronic sinusitis who were at higher risk of RTIs. Patients interested in participating returned reply slips, on which they self-reported their recent RTI history. We then purposively sampled from these responses to prioritise interviewing those with higher RTI frequency and co-morbid health conditions. We also sought variation with regards age and gender. We interviewed 13 participants in total.

For peer review only

Data collection

Interviews took place from April to August 2020, coinciding with the beginning of COVID-19 pandemic. Consequently, interviews were conducted by telephone. Participants provided written consent prior to taking part. Before the interview, participants answered brief questions about demographics and the number and type of RTIs they experienced.

Phase A: Think aloud interviews (n=10).

Participants were emailed a link to our prototype web-based intervention promoting nasal spray use for RTI prevention (Figure 2 provides an overview of this intervention). They worked through the website whilst simultaneously sharing their reactions aloud. The researcher prompted them to verbalise their thoughts and feelings as they encountered different pages, sections, messages, images and features.

Phase B: Post-intervention interviews (n=7)

Participants were emailed a link to the digital intervention (now optimised based on phase A feedback). A nasal spray was posted to them along with a short booklet summarising spray instructions. They were asked to use the website and the spray independently over a period of 2-3 weeks They then participated in an in-depth interview about their experiences. All participants also answered open-ended questions about their personal experiences of RTIs; findings from this part of the interview are published elsewhere[30].

Supplementary Material 4 contains the interview schedules. SW and LD conducted the interviews; both are female postdoctoral researchers with health psychology and qualitative interviewing expertise. Interviews lasted between 46 to 104 minutes and were audio-recorded and transcribed verbatim with identifying details removed. Participants received a £10 voucher to thank them for their time.

Figure 2: Overview of nasal spray intervention

[INSERT FIGURE 2 HERE]

For peer review only

Analysis

We used an inductive thematic analysis approach. Transcripts from phase A and B were analysed together. The analysts familiarised themselves with the audio-recordings and transcripts. Line-by-line coding of the data was conducted in Nvivo12 whereby codes were identified and labelled to capture references to perceptions or experiences of nasal sprays for preventing RTIs. The codes were then reviewed, compared, discussed and progressively clustered and merged in order to create themes. This was an iterative process which progressed to refining and organising final themes that captured important patterns and features in the data. SW and LD led the analysis, and all other authors were involved in interpreting, discussing and finalising themes. The research team have health psychology and medical backgrounds and the lead analysts are experienced qualitative researchers.

Ethics approval

For study 1, ethics and research governance approvals were granted by the University of Southampton (ERGOII:52394).

For study 2, ethics approvals were granted by NHS and the University of Southampton review boards (REC/HRA19/SC/0354; ERGO:48223).

Patient and Public Involvement (PPI)

A panel of PPI contributors with experience of recurrent RTIs and/or health conditions that mean they are vulnerable to frequent or severe infections have inputted into the study planning and conduct, some from the grant application stage. Contributions included editing and improving our participant information sheets, consent forms and interview schedules and participating in pilot interviews helping to interpret findings and drafting this paper and lay summary of the research findings sent to participants. Two members of the PPI panel are co-authors on this paper (DS and SRH).

This research has been reported in line with the COREQ checklist (Supplementary Material 5)

For peer review only

FINDINGS

Study 1: online consumer reviews of the nasal spray

Eight themes about nasal spray experiences were developed from the customer review data. These are described below and supporting quotations are provided in Table 1. The wording of illustrative quotations has been edited slightly to prevent the original reviews and reviewers being identifiable (e.g., through entering the quotation into a search engine). SW reworded the quotations, keeping meaning as close to the original as possible. LD checked and further edited reworded quotes to ensure it retained the meaning and could not be traced back to the original review.

Motivation to avoid infections

Reviewers described strong motivations to avoid becoming ill with cold-like illnesses. For some this was to avoid disruption to responsibilities and routines. Others were focused on avoiding unpleasant or severe symptoms or health complications for themselves or others that they might infect (e.g. vulnerable family members).

Inevitability of infections

Some reviewers were fatalistic about catching colds and similar infections and believed that symptoms would inevitably develop and progress despite using the spray.

Alternative approaches to infection prevention

Some reviewers described alternative, competing or perceived superior approaches to avoiding RTIs. This included measures such as good hand hygiene, healthy eating and vitamin supplements. Some expressed a perceived confidence in the body's own ability to fight off infections.

Recommendations from others

Reviewers sometimes described being influenced to buy and try the spray because of success stories and recommendations from others such as friends, family or HCPs.

For peer review only

Protection from risky situations

Some reviewers described adapting the way that the spray was used, beyond first signs and symptoms of an infection (i.e. recommended use as advised on product instructions). They adopted it as a preventative measure for when they perceived a high threat of infection, for example when travelling or in busy public places.

Ease of spray use

Reviewers often described sprays as quick and convenient to use and easily incorporated into daily life. However, some drew attention to the importance for correct technique and timely usage for efficacy. Some found that this is not always easily achievable.

Experiencing side effects

Reviewers commonly reported side effects including an unpleasant taste or feel in throat or nose, sinus pain, headache, or watery eyes. Side effects differed in severity across reviewers. When describing side effects, reviewers often referred to weighing up the experience of side effects against the impact of having a cold-like infection, reaching a range of conclusions about which was most desirable.

Expectations and experiences of success and failure

Some reviewers expressed confidence in the efficacy of the spray and referred to its ability to completely prevent colds and flu from developing or at least reduce the severity of symptoms and shorten their duration. Some reported lack of success or inconsistent results whereby sometimes infections happened despite use (although sometimes these were perceived as possibly milder than they would have otherwise been). Some reviewers emphasised the difficulties in judging whether the spray worked or not, given that it was uncertain how symptoms would have developed over time without spray use. However, doubts and uncertainties did not necessarily deter future use.

For peer review only

Table 1: themes from Study 1

Theme	Illustrative Quotations
Motivation to avoid infections	"As a mum, I can't afford to be ill – so it's wonderful that I now don't even though the rest of the family do."<br><br>"Because of my COPD I have to be careful cos colds can turn into a chest infection."
Inevitability of infections	"In my opinion, when you've got a cold there is no way to stop it."
Alternative approaches to infection prevention	"My body would probably have got rid of the cold – it usually does with vitamin c, drinking honey and using a salt water spray for my nose."<br><br>"In my opinion, if you don't touch your face (mouth, eyes and nose), this will prevent a cold. Germs live on surfaces for hours, so we need to be aware of this when we are out and about but especially if any of our family have an infection."
Recommendations from others	"I bought the spray because a nurse recommended it."<br><br>"My husband is a strong believer in this stuff."

For peer review only

Protection from risky situations	"I use it for the Tube where lots of people might be unwell - sneezing and stuff. The spray says to use it for when you have a cold coming but I have been using it every day regardless."<br><br>"I purchased it for when I go on holiday, when I usually catch infections when travelling by airplane. Since using it, I've not had any colds on my last two trips."
Ease of spray use	"The spray is easy to use and you can take it anywhere with you. I don't go anywhere without it."<br><br>"The instructions say you should aim towards your ear, and I thought I did do that. It's difficult to do it right."<br><br>"If you don't catch you first signs really early (e.g. the first odd feeling like tickling in the back of your throat) it will be too late. If your nose is already stuffy, it probably won't work."<br><br>"You must use the spray for a couple of days after your symptoms have gone away. If you stop when your symptoms are improving, your infection comes back."
Experiencing side effects	"The negative part is throat pain for 5 minutes or so, but that's the only negative. It's really bad pain but it's worth it to avoid getting a cold."<br><br>"I had extreme side effects. I don't want to have them again so I got rid of it. I reckon it works but the side effects were too bad for me!"
Expectations and experiences of success and failure	"Since the start of the year, I'd been unwell all the time. Then I used the spray at first signs and it stopped my cold (or at least made it tolerable and easier to deal with)."<br><br>"I've used the spray before and believed it had stopped my colds. However, it failed this time even though I followed the instructions exactly! The cold was the worst I've had in ages so now I just don't know if the spray DID work when I used it before."<br><br>"There's no way to be sure if my infection would have continued to get worse without the spray but, if there's any chance it was crucial in stopping the cold, then it's worth it!"

For peer review only

Study 2: Interviews about using a nasal spray to prevent RTIs

Participants

Table 2 describes the Study 2 participant characteristics.

Table 2: Demographic and clinical characteristics of Study 2 participants (n=13)

Characteristic	Summary Statistics
**Type of interview participation, N(%)**	
Think aloud interview only	8 (61.54%)
Post intervention interview only	3 (23.08%)
Both think aloud and post intervention	2 (15.38%)
**Age (years), mean (SD), range**	54.34 (22.24), 18-83
**Gender, N(%)**	
Men	3 (23.1%)
Women	10 (76.9%)
**Marital status, N(%)**	
Married or living with partner	5 (38.46%)
Single	3 (23.08%)
Divorced	2 (15.38%)
Widowed	3 (23.08%)
**Employment status, N(%)**	
In paid work (full or part time, employed, self-employed)	4 (30.77 %)
Retired	4 (30.77%)
Not working because of illness/disability	2 (15.38%)
Other (unemployed, homemaker, student)	3 (23.08%)
**Education (age left education), N(%)**	
16 or before	2 (15.38%)
17 or 18	3 (23.08%)
Over 18	8 (61.54%)
**Deprivation (IMD[1]), MDn (IQR), range**	10 (6.0), 3-10
**Ethnicity, N(%)**	
White British	7 (53.85%)
White Irish	1 (7.69%)
Mixed- White British/Asian	1 (7.69%)
Not provided	4 (30.77%)
**Health Conditions, N(%)** [2]	
Asthma	6 (46.15%)
COPD	2 (15.38%)
Chronic Sinusitis	1 (7.69%)
None of these conditions	7 (53.85%)
**Number of RTIs in last 12 months, Mean (SD), range**	2.92 (1.38), 1-5
**RTIs per year in last 3 years, N(%)**	
≥1	12 (92.31%)
≥3	7 (53.85%)

For peer review only

Types of RTIs experienced at least once in last 12 months, N(%)	
Cold	10 (76.92%)
Flu	2 (15.38%)
Throat infection	9 (69.23%)
Chest infection	7 (53.85%)
Sinus infection	6 (46.15%)
Ear infection	3 (23.08%)

[1]IMD= 2019 Index of Multiple Deprivation Decile, derived from participant postcodes, 1 is highest deprivation, 10 is lowest deprivation

2. The percentage totals more than 100 because 2 participants (15.38%) had more than one of these conditions

Themes

Eight themes were developed (Table 3). These are described below.

Table 3: Themes from Study 2

Theme	Illustrative Quotations
Excitement and optimism about a novel prevention method	"Then, when this came along it was like lightbulbs going off. I'm thinking, oh my God, this is going to be a way that I can safeguard myself and continue to be active within his life. I'm really excited about the uses of it." (Participant 10)<br>"I would quite happily give it a go." (Participant 11)<br>"A hundred per cent I'd be up for giving it a go." (Participant 5)<br>"I will give it a go I can tell you that now." (Participant 6)
Identifying first signs of infection	"I tend to just feel more rundown, tired, a bit headachy." (Participant 7)<br>"A lot of the times when I'm sneezing it's just because of my hay fever. It was quite difficult to tell." (Participant 9)<br>"If I know it's coming, by the time I'm doing something about it, I guess because my immune system's got no great strength, it's almost like too little, too late." (Participant 10)
Considering use in risky situations	"I can say, 'Well, I've got to go out. There's a chance I may be in contact with other people, so I'll use the spray'. It's like another layer of protection." (Participant 12)<br>"COVID-19 makes it more appealing, actually. I was quite intrigued about whether it would work for COVID." (Participant 11)<br>"I don't know, on a bus, supermarket, at the cinema, at the theatre… Like when you get home from the theatre you could start using it then, even if you haven't had any signs of anything. That was something that I thought was really useful to have. I could see that scenario." (Participant 10)

For peer review only

Consequences of feeling protected	"...that could only be helpful. I'm genuinely interested from those points of view, because I could get protection in the small part of my life where I'm allowing myself to be at risk, plus I think if I felt safer, I might therefore go out more and feel less frightened about the world." (Participant 10)<br><br>"It just meant that I could get on with things. Did I feel invincible? No, but I felt like I didn't have to worry too much, whereas I think if I was coming down with a cold I would have worried about work and being ill and not being able to complete work. I felt more relaxed, maybe, more confident." (Participant 11)<br><br>"But then would it encourage more people to actually go out and be slightly more reckless with sprays and masks and protection, washing their hands, touching their face because they're going, 'Oh, I'm using the spray, it's okay'. That's the other side of it." (Participant 12)
Concerns about medicines	"Part of it is because I don't like using medications, and I particularly don't like nasal sprays. I think over the last year or so I've used far too many and I'm a bit fed up of putting things in my nose. I think there's something off-putting about that." (Participant 11)<br><br>"I mean, to be fair, if it worked and it stopped me taking my medication, I'd much rather use a spray than medicine." (Participant 8)<br><br>"At the same time, I was like, oh, well if you don't have to ask a doctor and it's not an actual medication is it actually going to work?" (Participant 9)
Unpleasantness and hygiene	"It's not particularly pleasant, is it, watching people sticking things up their noses and their noses run and stuff." (Participant 11)<br><br>"You spray it up and then it all runs down. That sounds disgusting." (Participant 4)<br><br>"I was also worried that if I used it, it would pour everywhere. It didn't really." (Participant 9)<br><br>"I wouldn't [re]use anything that went into an orifice like an inhaler, or something I stuck up my nose, I wouldn't keep it and use it for another time." (Participant 10)
Familiarity and confidence	"I'm not very good at stuff like that. …I don't think I've ever really tried one [a nasal spray]. I'm just kind of wary of it." (Participant 9)<br><br>"It's common sense really. I've been using a [different type of] spray for years." (Participant 4)<br><br>"It's so straightforward using a nasal spray… I wouldn't bother with the video… Particularly at my age range, you've probably used nasal sprays several if not many times over your lifetime so you just would just use it." (Participant 1)

For peer review only

	“I think [I was] probably arrogant, I probably thought, 'Oh, for goodness sake, I don't need to be shown how to use a nasal spray!”' Although, clearly I did because once I used it as recommended, I didn't get a headache.” (Participant 13)
Reactions to possible or actual side effects	“I think it's good that it's listing the side effects, but they're not severe side effects. Obviously, if they're only very, very small, like causing a headache, you can take some paracetamol for that. If it stops you getting an infection or prolonging the infection, then a headache, just stopping that is very minor.” (Participant 5)<br><br>“I'd rather have that then a full-blown infection. That is on the plus side, even if it can cause a headache.” (Participant 8)<br><br>“I thought I'd try it again, and I did aim it more towards the ear, and although I did get a slight headache, it was much better and it went away very quickly.” (Participant 13).

Excitement and optimism about a novel prevention method

Overall, participants described positive and optimistic views about using the spray, seeing it as novel and of interest and personal relevance. For a few participants, there was a very pronounced excitement, with the spray seen as a way of transforming their quality of life. Others were more muted in their enthusiasm but still interested and willing to try the spray. Even participants who were not fully convinced that the spray would work, still considered it worth a try.

Participants found the explanations in the Immune Defence digital intervention about how the spray works to be understandable and plausible, in particular how the spray created an inhospitable environment for viruses. These ideas were particularly relevant and persuasive based on understandings about viruses and infection that participants were rapidly developing during the COVID-19 pandemic.

Identifying first signs of infection

Most participants were aware of their early RTI signs and felt able to recognise and react promptly to them by using a spray. However, sometimes participants found it difficult to tell whether a symptom signalled an oncoming infection. The crossover between hay fever and RTI symptoms was a particular area of uncertainty.

For peer review only

A minority of participants also described how they never experienced common early signs of infection and only became aware of oncoming illness through a severe symptom typical of a later stage of an infection (e.g., cough). Some therefore anticipated struggling to intervene in time.

Considering use in risky situations

Participants were particularly interested in using the spray in risky situations to prevent infections. Some participants considered that this mode of use may help to protect against COVID-19, although some remained cautious.

Some participants easily identified risky situations, where they would be happy to use the spray preventatively such as supermarkets, hospital appointments, concerts, airplanes and public transport. However, other participants debated or expressed uncertainty about what level of exposure would count as 'risky'. For some, most situations were currently considered risky (i.e., during the COVID pandemic). Others felt that if other mitigations were in place (such as social distancing or face masks) the spray was redundant for RTI prevention.

Consequences of feeling protected

A few participants anticipated that the protection against RTIs afforded by the spray would change how they felt, thought and behaved including feeling safer, less fearful more relaxed and more comfortable mixing with people with RTIs. A minority expressed concern that using the spray could lead to negative consequences for infection prevention behaviours. They speculated that other people (not themselves) might adopt less cautious behaviour overall. This concern appeared to be heightened by the COVID-19 context and included worries that, if other people were using the spray, they might be less likely to engage in other preventative behaviours such as masks and social distancing.

For peer review only

Concerns about medicines

Participants appeared to perceive RTI prevention nasal sprays as a form of medicine (the spray is officially a 'medical device'). Conceptualisation of the spray in this way seemed to persist for most participants to some degree despite encountering and understanding our intervention message that the spray is not a medicine and our comparison of regular spray use to regular hand sanitising. In line with perceiving the spray as a form of medicine, participants raised questions and concerns that are typical of medicines. For example, they were interested in ingredients and wanted to check for allergies, interactions or contra-indications with their routine medications. Participants also expressed apprehensions regarding over-use which they felt could lead to side effects, addiction or the spray becoming ineffective.

Participants often discussed trying to avoid using medicines. While this could raise concerns about using the spray, a few considered the spray a means of avoiding needing medication for RTI symptoms or disease exacerbations (e.g., antibiotics, steroids).

Although thinking of the spray as a medicine elicited concerns relating to medicines, thinking of the spray as something without medicine 'status' also appeared problematic; a minority of participants expressed slight doubt about how effective the spray could be if it was not a medicine, and not already regularly prescribed or recommended by the NHS.

Unpleasantness and hygiene

A few participants described how actions relating to noses and nasal mucous were unpleasant and socially unacceptable. A few (specifically those unfamiliar with using any type of nasal spray) found that the concept of a nasal spray inactivating and cleaning out viruses raised concerns about a messy and wet procedure. However, those who tried out the spray did not find this occurred. Given their awareness that viruses might be present in the nose, some participants were also concerned about how to use the spray hygienically. For example, they wondered whether germs left on the nozzle could infect them if they used the spray again later.

For peer review only

Familiarity and confidence

There was considerable variability in how much detailed information people felt they needed about exactly how to use the spray. This seemed to relate to lack of confidence and was prominent in participants who had not used any type of nasal spray before. One participant found using a spray daunting, was anxious about getting it right and found detailed instructions reassuring. Conversely, participants who had previously used another type of nasal spray appeared comfortable trying a spray and had fewer questions and concerns, seeing it as obvious and common-sense. However, this confidence could be unhelpful; one confident participant bypassed the instructions, tried the spray using the incorrect technique and experienced strong side effects. They described having thoughts about never using the spray again before realising the value of the technique instructions. Generally, people welcomed access to detailed guidance about spray technique and especially appreciated that the tips were aimed at helping them to reduce chance of side effects.

Reactions to possible or actual side effects

Participants considered knowing about the potential side effects of the spray important, paid keen attention to this information, but overall did not consider them off-putting. Participants stated that they would be willing to try the spray and would review their position and stop using the spray if bad side effects were experienced.

For peer review only

DISCUSSION

This paper is the first published research to explore how people think and feel about using nasal sprays, an emerging area of RTI prevention. Various important perceptions and experiences were identified which are discussed below in terms of their relevance for encouraging people to adopt and persist with this type of RTI prevention approach, if trial evidence supports their effectiveness.

Existing theory and research

Our findings align well with expectancy-value theories of health behaviour such as Health Belief Model[40] and the Necessity-Concerns Framework[34 35]. These theories emphasise implicit cost-benefit analysis; a person adopts and perseveres with preventative health behaviours generally or adherence to a medicine specifically based upon perceived efficacy, necessity and tolerability. We found strong beliefs about necessity in both studies. Study 1 participants wanted to avoid the physical and social impact of RTIs and study 2 participants (with recurrent RTIs or vulnerabilities to severe RTIs) welcomed our information and advice and considered sprays a novel and potentially effective prevention method. Considerable interest in strategies to prevent RTIs has been recently documented in vulnerable and/or recurrent patients[30] but research with younger and/or healthy participants in non-pandemic times reveals weaker or mixed beliefs about the necessity of avoiding infections[1 2 41-45]. Both studies reported here also highlighted a range of beliefs and concerns that could plausibly reduce engagement with using nasal sprays. Concerns around discomfort and regime complexity also arose in studies about nasal irrigation and sprays for sinusitis relief[4 29]. According to expectancy-value theories, reducing concerns and costs (alongside increasing necessity beliefs) will improve initiation and continuation of the behaviour.

A theoretical review[46] argues that medication adherence should be conceptualised as a type of causal learning and reasoning. People learn about how medications effect outcomes through a dynamic interplay of top-down (pre-existing beliefs and expectations about treatments) and bottom-up processes (personal experiences with symptom change and side effects, particularly early in the course of treatment). This learning influences their ongoing adherence. Causal learning theory[46] predicts that

For peer review only - http://bmjopen.bmj.com/site/about/guidelines.xhtml

learning a link between an intervention and positive outcomes (and therefore strong adherence) in the context of a nasal spray for RTI prevention could be challenging for several reasons. Firstly, people have limited data on which to reach conclusions from (e.g., several infections per year, rather than daily symptoms). Secondly, other variables confound interpretations of spray efficacy (e.g. other RTI prevention behaviours). Thirdly, sprays may not prevent infections 100% of the time, especially when use is suboptimal (timing, technique, dosage). Our findings about optimism about the spray are positive; people are likely to begin using sprays with expectancies that will facilitate interpreting a link between the spray and positive outcomes. However, some participants described doubt about effectiveness and some highlighted the difficulty of drawing strong conclusions from one's own experience. This, alongside the identified focus on side effects and concerns about using medicines, suggests that causal learning of a treatment benefit may be difficult and this may undermine adherence.

Finally, perceived ease or difficulty of using the spray and confidence for using it were also prominent within our findings. Social Cognitive Theory highlights self-efficacy as a key predictor of behaviour[47]. Intervention complexity and lack of confidence, alongside poor adherence have also been emphasised in research on nasal irrigation for sinus symptom relief[4 29].

Intervention development

We undertook the two studies reported here whilst developing the Immune Defence nasal spray intervention. Study findings informed the planning of initial intervention content (study 1) and optimisation of that content (study 2). For instance, our intervention content addressed concerns about overusing medicines, side effects and hygiene as well as avoided disgust reactions. We provided persuasive information to challenge fatalism about catching RTIs, helped people to build positive expectations of the spray and to continue to hold these even if it doesn't appear to work every time. We promoted the benefits of feeling protected, whilst explaining the importance of continuing other RTI prevention behaviours. We emphasised the simplicity of spray use (and ensured a straightforward experience via clear, easy instructions) and we presented information to suit both experienced nasal spray

users and less confident beginners. Supplementary Materials 6 provides further details about how study findings influenced intervention content.

Strengths, limitations and future research

A key strength of this paper was its combination of findings from different samples and data collection methods allowing insights into a variety of people and experiences. Some of our data reflects experiences of people who were already motivated to buy the spray and who had some experience of using it, but we also gathered data from people for whom RTI prevention is clinically relevant but who did not currently use nasal sprays. We also collected data from pre-COVID-19 and early pandemic contexts.

Study 1 was a large sample but collected and analysed thin, brief data with little contextual information and no knowledge of reviewer demographic and clinical characteristics. Furthermore, the reviews cannot be verified as genuine as they were on commercial websites. However, the details of problems, concerns and doubts that were largely supported (and extended) in Study 2 gives confidence that we have captured genuine data.

Study 2 examined reactions to the Immune Defence intervention content allowing insight into what is interesting, confusing, concerning, off-putting about the nasal spray as described by a *specific rationale and set of instructions*. Whilst some of the detail is therefore particularly pertinent to the Immune Defence nasal spray intervention, the overall themes may be generalisable to other nasal sprays and similar products, prevention behaviours, instructions and advice. Phase b of Study 2 was designed to explore how people experience beginning to use the spray for the first time. A significant limitation, however, is that only 7 participants took part in this phase. They also tried the spray over just three weeks, in a partial COVID-19 national lockdown and during the summer months. They therefore experienced little exposure to viruses and consequently had limited opportunity to use the spray in the intended ways. Tracking more participants over longer periods will provide a clearer picture of usage and adherence and will be particularly useful for shedding light on factors that may only become apparent over time (e.g. experiencing or not experiencing benefits). Qualitative and quantitative data collection on spray

For peer review only - http://bmjopen.bmj.com/site/about/guidelines.xhtml

adherence, experiences and beliefs is currently in progress as part of the Immune Defence process evaluation.

Whilst our findings suggest nasal sprays for RTI prevention are of interest to clinically higher risk subgroups and considered particularly valuable in the pandemic context, whether lower risk groups (e.g. healthy adults) have similar perceptions has not been established. Furthermore, some of the recent and current trials of nasal sprays and similar approaches relate specifically to HCPs at risk during provision of medical care[24]. Findings about lay people's motivations, facilitators and barriers may not transfer well to HCPs; their expertise and the occupational setting may mean different factors are important. Additional research may therefore be needed with these groups.

Conclusion

People who suffer frequent or severe infections or who are clinically vulnerable to RTIs are interested in using a nasal spray to prevent RTIs and see this as useful or even a 'game changer'. They also have some doubts and concerns and may expect to encounter (or actually encounter) certain difficulties. Many of the information needs, misunderstandings, concerns and difficulties exposed through the current research may be remedied by ensuring interventions are designed to help people overcome these issues.

For peer review only

Author Contributions

LY, AG and PL conceived the study idea and initial study design with later input from BA, LD, SW, KG, FM, JDD, KB, SRH and DS. SW led data collection with assistance from LD. SW, LD and FM led data analysis with input from all authors at different stages. LD and SW drafted the manuscript. All authors contributed to critically editing and approving the final manuscript.

Other contributors: Kate Martinson managed ethical approvals and recruitment. Thank you also to all our PPI panel members, in particular Hazel Patel, Samantha Richards-Hall and Debs Smith.

Competing interests: none declared.

Data availability statement: No additional data available.

Funding: This study/project is funded by the National Institute for Health Research (NIHR) Programme Grants for Applied Research (PGfAR) Programme. The views expressed are those of the author(s) and not necessarily those of the NIHR or the Department of Health and Social Care.

This study was nested within a NIHR Programme Grant for Applied Research: REducing Common infections in Usual practice for Recurrent Respiratory tract Infections (RECUR). (Prof Paul Little, Dr Adam Geraghty) [RP-PG-0218-20005]. Lucy Yardley is an NIHR Senior Investigator and her research programme is partly supported by NIHR Applied Research Collaboration (ARC)-West, NIHR Health Protection Research Unit (HPRU) for Behavioural Science and Evaluation, and the NIHR Southampton Biomedical Research Centre (BRC).

The research programmes of Lucy Yardley and James Dennison-Day are partly supported by the NIHR Southampton Biomedical Research Centre (BRC). The intervention development methods used for the RECUR/"Immune Defence" intervention were developed with support from the NIHR Southampton Biomedical Research Centre (BRC).

For peer review only

REFERENCES

1. Mayrhuber EA-S, Peersman W, van de Kraats N, et al. "With fever it's the real flu I would say": laypersons' perception of common cold and influenza and their differences-a qualitative study in Austria, Belgium and Croatia. *BMC Infectious Diseases* 2018;18(1):1-9.
2. Cedraschi C, Saya L, Klein P, et al. Representations of influenza and influenza-like illness in the community-a qualitative study. *BMC Family Practice* 2013;14(1):1-9.
3. McSweeney L, Rousseau N, Wilson J, et al. Stakeholders' views of recurrent sore throat, tonsillitis and their management: a qualitative interview study for the NA tional Trial of Tonsillectomy IN Adults (NATTINA Part 1). *Clinical Otolaryngology* 2017;42(2):301-06.
4. Leydon GM, McDermott L, Thomas T, et al. 'Well, it literally stops me from having a life when it's really bad': a nested qualitative interview study of patient views on the use of self-management treatments for the management of recurrent sinusitis (SNIFS trial). *BMJ open* 2017;7(11):e017130.
5. Snell LM, Wilson RP, Oeffinger KC, et al. Patient and physician explanatory models for acute bronchitis. *Journal of family practice* 2002;51(12):1035-41.
6. Statistics OoN. Sickness absence in the UK labour market. 2020 [Available from: https://www.ons.gov.uk/employmentandlabourmarket/peopleinwork/labourproductivity/articles/sicknessabsenceinthelabourmarket/2020.
7. Finley CR, Chan DS, Garrison S, et al. What are the most common conditions in primary care?: Systematic review. *Canadian Family Physician* 2018;64(11):832-40.
8. Morbey R, Elliot A, Harcourt S, et al. Estimating the burden on general practitioner services in England from increases in respiratory disease associated with seasonal respiratory pathogen activity. *Epidemiology & Infection* 2018;146(11):1389-96.
9. Gulliford MC, Dregan A, Moore MV, et al. Continued high rates of antibiotic prescribing to adults with respiratory tract infection: survey of 568 UK general practices. *BMJ open* 2014;4(10):e006245.
10. Nowakowska M, Van Staa T, Mölter A, et al. Antibiotic choice in UK general practice: rates and drivers of potentially inappropriate antibiotic prescribing. *Journal of Antimicrobial Chemotherapy* 2019;74(11):3371-78.
11. Jefferson T, Del Mar CB, Dooley L, et al. Physical interventions to interrupt or reduce the spread of respiratory viruses. *Cochrane database of systematic reviews* 2020(11)
12. Saunders-Hastings P, Crispo JA, Sikora L, et al. Effectiveness of personal protective measures in reducing pandemic influenza transmission: A systematic review and meta-analysis. *Epidemics* 2017;20:1-20.
13. Lee JK, Lam GK, Shin T, et al. Efficacy and effectiveness of high-dose versus standard-dose influenza vaccination for older adults: a systematic review and meta-analysis. *Expert review of vaccines* 2018;17(5):435-43.
14. Polack FP, Thomas SJ, Kitchin N, et al. Safety and efficacy of the BNT162b2 mRNA Covid-19 vaccine. *New England Journal of Medicine* 2020
15. Voysey M, Clemens SAC, Madhi SA, et al. Safety and efficacy of the ChAdOx1 nCoV-19 vaccine (AZD1222) against SARS-CoV-2: an interim analysis of four randomised controlled trials in Brazil, South Africa, and the UK. *The Lancet* 2021;397(10269):99-111.
16. Martineau AR, Jolliffe DA, Hooper RL, et al. Vitamin D supplementation to prevent acute respiratory tract infections: systematic review and meta-analysis of individual participant data. *bmj* 2017;356
17. Jayawardena R, Sooriyaarachchi P, Chourdakis M, et al. Enhancing immunity in viral infections, with special emphasis on COVID-19: A review. *Diabetes & Metabolic Syndrome: Clinical Research & Reviews* 2020;14(4):367-82.

For peer review only

18. Grande AJ, Keogh J, Silva V, et al. Exercise versus no exercise for the occurrence, severity, and duration of acute respiratory infections. *Cochrane Database of Systematic Reviews* 2020(4)
19. Barrett B, Hayney MS, Muller D, et al. Meditation or exercise for preventing acute respiratory infection: a randomized controlled trial. *The Annals of Family Medicine* 2012;10(4):337-46.
20. Kramer A, Eggers M, Hübner N-O, et al. Virucidal gargling and virucidal nasal spray. *GMS Hygiene and Infection Control* 2021;16
21. Bichiri D, Rente AR, Jesus Â. Safety and efficacy of iota-carrageenan nasal spray in treatment and prevention of the common cold. *Medicine and pharmacy reports* 2021;94(1):28.
22. Cegolon L, Javanbakht M, Mastrangelo G. Nasal disinfection for the prevention and control of COVID-19: A scoping review on potential chemo-preventive agents. *International journal of hygiene and environmental health* 2020;230:113605.
23. Burton MJ, Clarkson JE, Goulao B, et al. Antimicrobial mouthwashes (gargling) and nasal sprays administered to patients with suspected or confirmed COVID-19 infection to improve patient outcomes and to protect healthcare workers treating them. *Cochrane Database of Systematic Reviews* 2020(9)
24. Burton MJ, Clarkson JE, Goulao B, et al. Antimicrobial mouthwashes (gargling) and nasal sprays to protect healthcare workers when undertaking aerosol-generating procedures (AGPs) on patients without suspected or confirmed COVID-19 infection. *Cochrane Database of Systematic Reviews* 2020(9)
25. Casale M, Rinaldi V, Sabatino L, et al. Could nasal irrigation and oral rinse reduce the risk for COVID-19 infection? *International Journal of Immunopathology and Pharmacology* 2020;34:2058738420941757.
26. Radulesco T, Lechien JR, Saussez S, et al. Safety and impact of nasal lavages during viral infections such as SARS-CoV-2. *Ear, Nose & Throat Journal* 2021;100(2_suppl):188S-91S.
27. Posch W, Vosper J, Zaderer V, et al. ColdZyme maintains integrity in SARS-CoV-2-Infected airway epithelia. *MBio* 2021;12(2):e00904-21.
28. Yardley L, Morrison L, Bradbury K, et al. The person-based approach to intervention development: application to digital health-related behavior change interventions. *Journal of medical Internet research* 2015;17(1):e4055.
29. Vennik J, Eyles C, Thomas M, et al. Management strategies for chronic rhinosinusitis: a qualitative study of GP and ENT specialist views of current practice in the UK. *BMJ open* 2018;8(12):e022643.
30. Dennison L, Williamson S, Greenwell K, et al. Patient perceptions of vulnerability to recurrent respiratory tract infections and prevention strategies: a qualitative study. *BMJ open* 2022;12(4):e055565.
31. Rogers RW. A protection motivation theory of fear appeals and attitude change1. *The journal of psychology* 1975;91(1):93-114.
32. Maddux JE, Rogers RW. Protection motivation and self-efficacy: A revised theory of fear appeals and attitude change. *Journal of experimental social psychology* 1983;19(5):469-79.
33. Bandura A. Health promotion by social cognitive means. *Health education & behavior* 2004;31(2):143-64.
34. Horne R, Weinman J, Hankins M. The beliefs about medicines questionnaire: the development and evaluation of a new method for assessing the cognitive representation of medication. *Psychology and health* 1999;14(1):1-24.
35. Clifford S, Barber N, Horne R. Understanding different beliefs held by adherers, unintentional nonadherers, and intentional nonadherers: application of the necessity–concerns framework. *Journal of psychosomatic research* 2008;64(1):41-46.
36. Leventhal H, Nerenz DR, Steele DJ. Illness representations and coping with health threats. Handbook of Psychology and Health (Volume IV): Routledge 2020:219-52.
37. Hagger MS, Orbell S. A meta-analytic review of the common-sense model of illness representations. *Psychology and health* 2003;18(2):141-84.

For peer review only

38. O'Cathain A, Croot L, Duncan E, et al. Guidance on how to develop complex interventions to improve health and healthcare. *BMJ open* 2019;9(8):e029954.
39. Skivington K, Matthews L, Simpson SA, et al. A new framework for developing and evaluating complex interventions: update of Medical Research Council guidance. *bmj* 2021;374
40. Rosenstock IM. Why people use health services. *The Milbank Quarterly* 2005;83(4)
41. Lundgren B. The Common Cold, Influenza and Immunity in Post-Pandemic Times: Lay representations of Self and Other among older people in Sweden. *Health, culture and society* 2015;8(2):46-59.
42. Yardley L, Miller S, Teasdale E, et al. Using mixed methods to design a web-based behavioural intervention to reduce transmission of colds and flu. *Journal of Health Psychology* 2011;16(2):353-64.
43. Seale H, Mak JP, Razee H, et al. Examining the knowledge, attitudes and practices of domestic and international university students towards seasonal and pandemic influenza. *BMC Public Health* 2012;12(1):1-6.
44. Prior L, Evans MR, Prout H. Talking about colds and flu: the lay diagnosis of two common illnesses among older British people. *Social Science & Medicine* 2011;73(6):922-28.
45. Petricek G, Hoffmann K, Vandenbroucke A, et al. Laypersons' perception of common cold and influenza prevention—a qualitative study in Austria, Belgium and Croatia. *European Journal of General Practice* 2019;25(4):220-28.
46. Rottman BM, Marcum ZA, Thorpe CT, et al. Medication adherence as a learning process: insights from cognitive psychology. *Health Psychology Review* 2017;11(1):17-32.
47. Bandura A. Health promotion from the perspective of social cognitive theory. *Psychology and health* 1998;13(4):623-49.

For peer review only

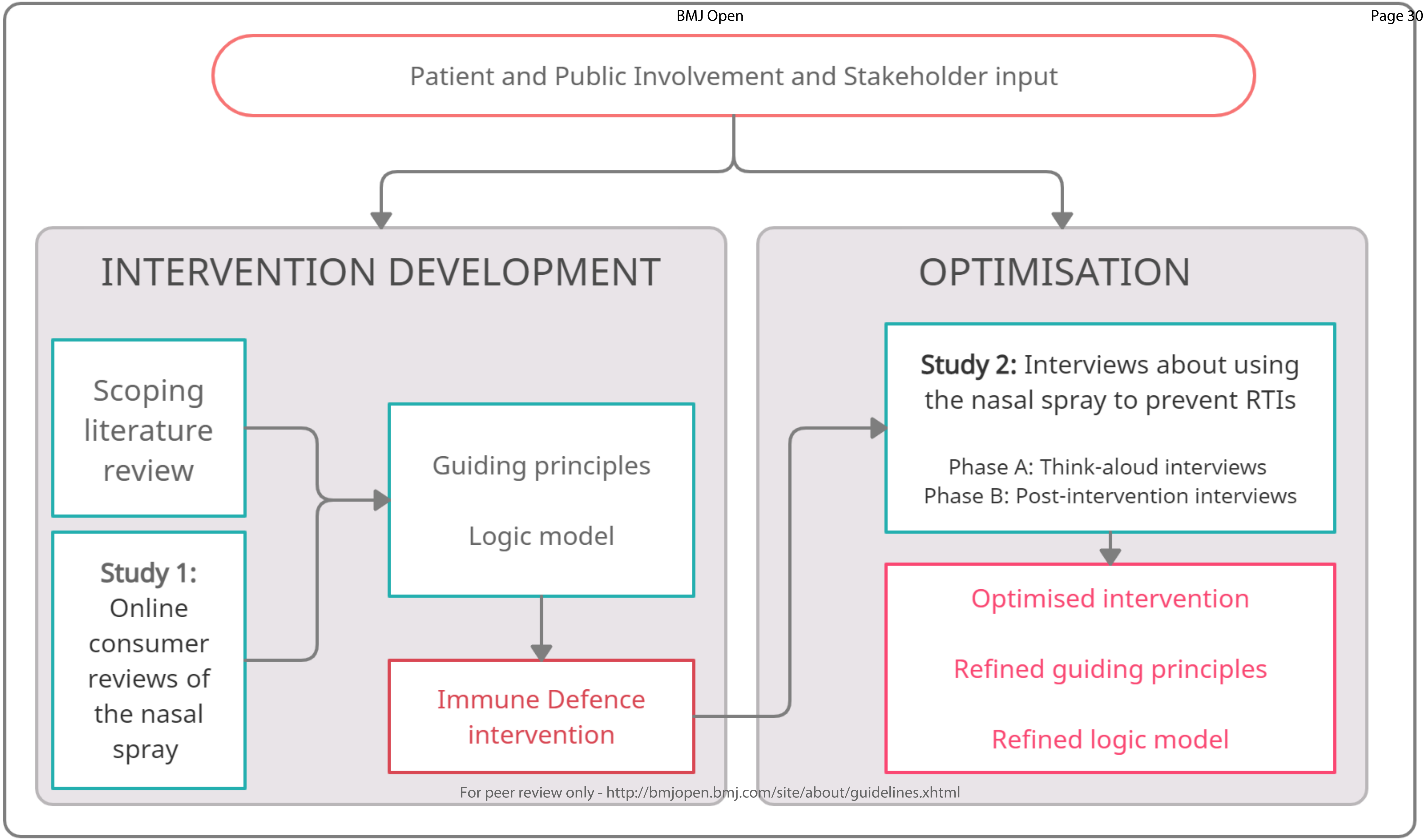
Patient and Public Involvement and Stakeholder input
INTERVENTION DEVELOPMENT
Scoping literature review
Study 1: Online consumer reviews of the nasal spray
Guiding principles
Logic model
Immune Defence intervention
OPTIMISATION
Study 2: Interviews about using the nasal spray to prevent RTIs
Phase A: Think-aloud interviews
Phase B: Post-intervention interviews
Optimised intervention
Refined guiding principles
Refined logic model

NASAL SPRAY INTERVENTION

"Immune Defence" website

Summarises RTIs and their negative impact

Explains RTI transmission

Explains spray mechanism

Encourages spray use

Explores how the spray can be used (reactively/preventatively)

Teaches spray technique (Inc. video)

Provides dosage information

Addresses concerns about using sprays

A supply of nasal spray

Sent by post

2 initial bottles

More available by request

Provision of a booklet

Sent by post

Summarises key messages from the website

Reminds users to return to the website for more information and support

Automated emails

Sent every 4 weeks

Repeats key messages from the website

Reminds about how and when to use the spray

Addresses concerns

Provides persuasive information about the benefits

For peer review only - http://bmjopen.bmj.com/site/about/guidelines.xhtml

Supplementary material 1: RECUR ("Immune Defence") Guiding principles – Nasal sprays

User context	Key design objective	Intervention design features
Our users have a range of nasal spray experience. Some have used sprays before, such as for hayfever, but perhaps not using the techniques we recommend for this spray. Some have never used a nasal spray before.<br><br>The idea of using sprays preventatively is a novel idea for most target users.	To support new and experienced nasal spray users to build skills to develop a correct spray technique.	• Provide an instructional video on a 'live model' demonstrating correct spray technique and modelling behaviour to build skills for new users.<br>• Provide an instructional booklet to be kept with the spray for ease of use and a reminder of how to use the spray correctly.<br>• Highlight specific advice which will be helpful for new and experienced sprays users (e.g. do not inhale deeply when the spray is in your nose).<br>• Advise everyone to watch the video including experienced sprays users, highlighting that this spray is administered in a different way.
Our target group perceive the spray as exciting and they are hopeful it will work. However, they may not always experience benefits when they begin using it and this could lead to disappointment and (depending on other factors, including side effects) discontinuation or suboptimal adherence. Some users also have previous experience of trying other prevention strategies with little success, leading to a fatalistic perspective of RTIs.	To support people to set and maintain positive expectations of spray efficacy, even when RTIs still occur.	• Explain the mechanism of how the spray works so that people understand that the aim of the spray is to reduce viral load not prevent viruses entering the body at all.<br>• Emphasise how sprays reduce the duration and severity of an RTI, as well as preventing them in the first place, to avoid feelings of disappointment if people do get an RTI after using the spray.<br>• Address the potential lack of efficacy beliefs by providing advice for future use (e.g. using the spray quickly at first signs of infection, using the correct technique) and motivating the user to keep trying the spray, even if they got an infection.

For peer review only

Concerns about nasal spray side effects are fairly common and may lead to discontinuation of use.	To reduce people's concerns about possible negative effects of spray use.	• Provide reassuring information to address concerns about potential side effects, specifically regarding the mildness and tolerability of the spray.<br>• Change perceptions of side effects – framing them positivity as 'normal' by describing what to expect. Explaining side effect symptoms as a sign that the spray is working effectively (e.g. getting to the areas it needs to in order to work).<br>• Compare side effects to worse outcomes if nasal spray was not used e.g. getting an infection.<br>• Provide advice and skills training on how to optimise your nasal spray technique to avoid side effects.<br>• Advise on how to cope with side effects if they did happen e.g. how to deal with a nosebleed.
People may see nasal sprays as medicines because of their mode of administration and previous experiences with sprays that are medicines (e.g. hayfever, sinus). Many people have concerns about over-use of medicines.	To help develop an alternative way of thinking about the spray, to reassure people about safety and to persuade people that nasal sprays are safe.	• Explain that nasal sprays work in a similar way to handwashing/hand gel. This provides a familiar example of something that is not a medicine but helps prevent infections. Both prevention methods are common behaviours, simple and acceptable, and they neutralise/remove germs/viruses before they can infect you and make you ill.<br>• Address the concern that the spray is a medicine by clarifying that it is not a medicine and that it is safe and non-addictive.

For peer review only

The intervention will be delivered during the COVID-19 pandemic where the threat levels and national recommendations are constantly changing.<br><br>Emerging evidence suggests that nasal sprays may be useful in providing additional protection against viruses like COVID-19, which act similarly to common RTIs but have severe consequences, particularly for vulnerable groups.<br><br>Our target group clearly spot the similarities between COVID and seasonal/normal RTIs. Those using the intervention in a pandemic context may be concerned with either or both COVID/normal RTIs.<br><br>Some of our target group have incorrect beliefs that the spray can replace government COVID recommendations or that the spray is not needed because of other behaviours mitigating the usefulness (e.g. mask wearing, social distancing).	To ensure the intervention is suitable for delivery during rapidly changing COVID-19 pandemic context.	• Be able to quickly update intervention content when needed to reflect latest guidelines and research evidence.<br>• Explain how effective the spray might be for COVID-19.<br>• Correct misconceptions about nasal sprays and COVID-19 by explaining that the spray is another layer of protection to be used with other behaviours to ensure the best protection possible again infections.

For peer review only

Supplementary material 2: Logic model

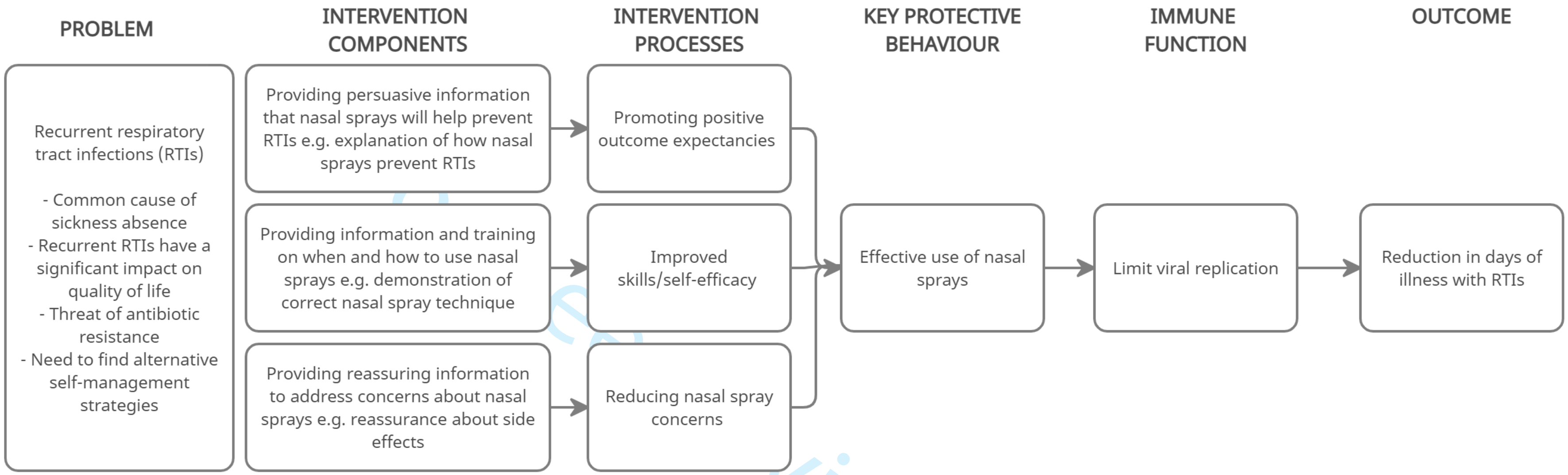

Supplementary material 3: Table of Changes extract

This is a simplified extract from a "table of changes" used to optimise the Immune Defence nasal spray intervention. It has been edited for clarity for readers outside of research team.

Website Section/content referred to	Original website content wording	Participant comments: positive, or likely to promote engagement/adherence	Participant comments: negative, or likely to impair engagement/adherence	Action/Changes	Final website content wording
"When do I use the spray?"<br><br>Description of first signs of an infection that should trigger use of the spray.	There are 3 ways to use the spray.<br><br>**1.When you first feel an infection coming on.**<br><br>It works best if you use it **as soon as you notice any symptoms.**<br><br>Often people say their first signs are:<br>• Sneezing;<br>• A runny nose;<br>• A tickle in the nose; or<br>• A tickle in the back of the throat	**INTERVIEWER:** So what would you say are your kind of first signs of infection? **PARTICIPANT 1:** Mine are usually sneezing and a tickle. **INTERVIEWER** Yes, so you'd be able to identify yourself in those symptoms? **PARTICIPANT 1:** Oh yes, yes.<br><br>**PARTICIPANT 3:** So I suppose when I first feel an infection coming on is when the sore throat starts. **INTERVIEWER:** So that would be your first sign of an infection happening? **PARTICIPANT 3:** That would be the warning sign, definitely.<br><br>**PARTICIPANT 4:** [participant reads website] "*Often people say the first signs are sneezing*", yes, agree with that; "*runny nose*", yes; "*tickle in the back of the nose or a tickle in the back of the throat*", yes. The other thing is a headache or feeling hot and cold - feeling hot is another sign for me anyway.<br><br>**PARTICIPANT 3**: It's great. It's absolutely everything that I and my family feel and experience.	**PARTICIPANT 7:** I think that makes sense, and you haven't said, 'These are necessarily the signs that you would get when you feel an infection coming on.' You say, 'That's what often people say they are,' so... **INTERVIEWER**: So are your first signs recognisable in there, or not? **PARTICIPANT 7:** No. I don't know. I think I tend to just feel more rundown, tired, a bit headachy. I don't know. I wouldn't say I get a runny nose at all. No, I wouldn't say they are, to be honest!<br><br>**PARTICIPANT 1:** I can't think of any other additional things that would indicate that I had a nasal infection coming on. I would perhaps personally, sometimes I get a thick throat, like the equivalent of catarrh building up… but whether that comes under a tickle, I don't know, but that's what I personally would get as an indication, like just a thickening of the mucus<br><br>**INTERVIEWER**: Would you say those first signs of infection are similar to what you experience, or is it different? **PARTICIPANT 10**: Yes.	We added a catch-all statement about a wide range of early signs of RTIs, acknowledging idiosyncrasies and building confidence in spotting own first signs.	There are 3 ways to use the spray.<br><br>**1.When you first feel an infection coming on.**<br><br>It works best if you use it **as soon as you notice any symptoms.**<br><br>Everyone's first signs of infection are different. Often people say their first signs are:<br>• Sneezing;<br>• A runny nose;<br>• A tickle in the nose;<br>• A tickle in the back of the throat;<br>• Your skin feeling sensitive;

For peer review only

			I think the first signs, they are but the first signs are often an aching, aren't they and sore? I get sore skin and aching li-, just a general ache as a first sign quite often of these bugs. So sometimes they are, but I suppose that might be the difference between colds and flu, I don't know. Sore throat I'd say, rather than a tickle in the back of the throat, but you say often…I'm not sure about these symptoms. Maybe sometimes, but not always. I think for me I often feel achy and have this very funny sensitive skin which seems a bit sore all the time, that would be the first sign, but maybe as I say, that's the difference between flu and cold, I don't know. If that's what people say then that's what most people say then that's a thing, isn't it?		• Having slightly achy muscles; or<br>• Having a mild headache.
"How does the spray work?"<br><br>Comparison of using the nasal spray to washing.<br><br>In various places the content refers to a washing/washing out metaphor and a comparison to washing hands with soap and	It's a bit like using soap when you wash your hands, only better.<br><br>The spray traps viruses and washes them out of the nose; and make the nose and throat **a very unfriendly place for viruses.**<br><br>This means it's **much harder for them to survive**	**PARTICIPANT 4** [Reading website] How does it work? *"It's a bit like using soap and wash your hands only better!"* Oh, I like that.<br><br>**INTERVIEWER:** What sounds particularly appealing about giving it a go? **PARTICIPANT 5**: It says that, well, the whole bit of how does it work? *"The spray traps the viruses and washes them out of your nose. Makes your nose and throat a very unfriendly place for the viruses. This means it's much harder for them to survive".* I'd be willing to give that a go!	**PARTICIPANT 4**: The spray traps the viruses and washes them out of the nose. Ooh, how does it wash it out of the nose? You spray it up and then it all runs down. That sounds disgusting. Do you spray it and then blow out? **INTERVIEWER**: You've got a question there about how to use it essentially, is that right? **PARTICIPANT 4**: It says here just - well, I know you know what it says but, *'Spray traps the viruses and washes them out of the nose.'* All right. Let's read the next sentence. "*Makes the nose and throat a very unfriendly place for viruses. This means it's much harder for them to*	We retained the hand cleaning metaphor but change to a comparison with hand gel rather than soap and water to provide a closer match to the spray action and avoid the procedure sounding difficulty or unpleasant. Hand gel use is currently (early	It's a bit like a hand gel, but specially designed for your nose.<br><br>The spray helps to clean the virus from your nose.<br><br>The spray also makes the nose and throat a very **unfriendly place for viruses.** This means it's much **harder for them survive** so they

For peer review only

water to remove germs before they can do harm.	so they can't take hold and make you ill.	**INTERVIEWER:** Does the explanation about how it works make sense to you, about the soap and hands? **PARTICIPANT 3:** Yes, indeed. Yes, I'll say!<br><br>**INTERVIEWER:** and the explanation saying 'it's a bit like using soap when you wash your hands', does that seem to make sense to you? **PARTICIPANT 1:** Well, it does, particularly in the current situation with coronavirus!<br><br>**INTERVIEWER:** in terms of that explanation about how it works, being like soap for washing your hands, does that make sense to you, that explanation? **PARTICIPANT 7:** Yes, it does. I'm wondering if soap changes your pH levels because I've never really thought about that! It makes sense though, yes, and you said it traps viruses and washes them out of the nose, so that makes sense, I think.	*survive so they can't take hold and make you ill".* Right. Well, I'd like to know this washing out of the nose... **INTERVIEWER** Yes, because your first reaction to that was that sounds a bit gross - almost a bit disgusting. **PARTICIPANT 4**: Yes! It's like do I spray the liquid up my nose and then it all runs down my face? Or are you meant to spray it up your nose; then sniff it up; and then you swallow it? Which sounds equally disgusting I might add. I don't like that... The spray traps viruses, absolutely happy with that but washes them out of the nose has all sorts of horrible connotations.<br><br>**INTERVIEWER** You were anticipating almost that it has to come out of the nose essentially whereas this is suggesting... **PARTICIPANT 4**: Yes, exactly. On that video, all you're doing is placing the liquid spray into the nose as the barrier to the virus but there's no washing out. It's like putting some deodorant on. You put it on, and you leave it in place because it's got a job to do.	COVID pandemic) a common anti-infection product people are using with confidence.	can't take hold and make you ill.
Video – instructions how to use the spray	Part of the video demonstrates high risk situations in which you should use the nasal spray. It depicts a person being sneezed on by	-	**INTERVIEWER**: I heard you had a little bit of a giggle at one point. **PARTICIPANT 3**: [Laughs] Yes, that was just the second person sneezed all over the girl who was doing the demonstration.	No change. Content is engaging, enjoyable (and the intended message was clearly understood).	-

	somebody else (acting).				
Video – instructions how to use the spray	Part of the video demonstrates exactly how to prime the spray, insert into nostrils, spray and sniff.	**PARTICIPANT 5:** Yes, no, I think it's good. It's very informative and it's good that it gives people, like it shows people how to correctly use the spray because sometimes, the instructions on the boxes and in the packets and things, they aren't always as clear. **INTERVIEWER**: Do you think you'd find that useful yourself if you were trying out the spray for the first time? **PARTICIPANT 5:** Yes, definitely.	**INTERVIEWER:** we do have a link there to a video. You don't need to click on that. I just wanted to ask you, with the information that's here would you need to click on that link to see the video, or do you think that you'd be okay with the instructions there? **PARTICIPANT 7:** I wouldn't because obviously I've used sprays before. Somebody that hasn't used, and is a bit wary of it, would probably click on it.<br><br>**INTERVIEWER** Would you be inclined to watch the video yourself if you wanted instructions, or would you be more…? **PARTICIPANT 1**: No, to be honest, it's so straightforward using a nasal spray… I wouldn't bother with the video… Particularly at my age range, you've probably used nasal sprays several if not many times over your lifetime so you just would just use it.<br><br>**INTERVIEWER** What did you think about that video? **PARTICIPANT 4**: Yes, it's common sense really. I've been using [another] spray for years... I do keep my head straight. I do do my one or two good puffs. I don't breathe out and spray it everywhere. It's common sense.<br><br>**PARTICIPANT 2** When I first got [the spray], I used it once, even though I didn't have any need to use it and	We attempted to get more people to watch the video, by emphasising how the way of using the spray might be different.<br><br>Given that we know that incorrect use/angle can increase likelihood of side effects, and that our instructions are different to other sprays (e.g. hay fever, sinus medications) it is vital people use it as per instructions rather than according to common sense.	Click **here** to see a short video to help you master the spray technique.<br><br>This video is worth watching even if you have used nasal sprays before. The technique for **this spray** might be a bit different. Using the spray correctly give you the best chance of fighting infections!

			within seconds it had given me a headache, exactly as it did the very first time I used it about a year or so ago on the recommendation that was given to me. I felt, right, I'm not going to use it anymore. The only way I can describe the headache is it's like a freezer headache. It's exactly the same, if you take a bite of an iced lolly or something. That sort of, right between the eyes. I read about how it says that if that happens, you should aim it more towards your ear, rather than straight up…..[later in interview]……**INTERVIEWER**: Why did you decide not to watch the video? **PARTICIPANT 2:**I think I was probably rushing off to do something, or I got distracted or, no, I didn't. I think probably arrogant, I probably thought, 'Oh, for goodness sake, I don't need to be shown how to use a nasal spray.' Although, clearly I did because once I used it as recommended, I didn't get a headache.		

Readers interested in using the Person-Based Approach to intervention development and who wish to use a Table of Changes to assist the process may find resources on this website helpful: https://www.lifeguideonline.org/pba

Supplementary material 4:

Study 2 - Interview Schedules for Phase A and Phase B

Phase A: Think-aloud interviews

Prompts about key pages of intervention content [e.g. menus/ first page etc.]

- What are your first impressions of this page?
- What are you thinking now?
- What made you choose that option?
- What do you think about [this activity, this information, this strategy/tool/idea]?
- Can you tell me a bit more about why you think that?
- [in response to an expression of like/dislike] What is it you like/don't like about that?
- That's really interesting…..
- [picking up on vocalisations/tone of voice etc] I noticed that you paused/groaned/laughed/sighed etc. at……… Can you tell me what you thought about that?

After working through the key pages of intervention content:

- Overall, what do you think about the web pages?
- Can you tell me about anything you thought was particularly good about the web pages?
- Can you tell me anything about the web pages that you were less keen on?
- Which parts did you find most relevant to you? Which parts were the least relevant to you?
- Having looked at the web pages, can you tell me how you feel about trying to use a nasal spray to try to reduce these sorts of infections
- How much of what you've seen today do you think is relevant to coronavirus?*
- How at risk do you feel about getting these infections at the moment?
- What do you feel about the recommendation to use the spray when at high risk and how this applies to coronavirus?*
- What device did you use to look at the website today?
    - If you were using the website over a longer period of time, how would you access the website?
    - Would you use mobile phone at all?

For peer review only

Phase B: Post-intervention interviews

- Can you start by telling me overall how you got on with trying the spray?

Questions if they have tried the spray:

- Can you tell me all about how you found using the nasal spray?
- Can you tell me about anything you liked or found easy?
- Can you tell me about anything you disliked or found difficult?
    - Can you let me know if there was anything you found helped with that?
    - Can you tell me about anything that worried you about using the spray?
- Can you tell me about when you used the nasal spray?
    - When did you think to use it?
    - Can you tell me about whether any situations came up where you could have used the spray (e.g. first symptoms, feeling a risk of catching an infection)? Can you tell about how you decided whether to use the spray?
- Can you tell me about what you thought were the advantages of using the nasal spray?
- Can you tell me about what you thought were the disadvantages of using the nasal spray?
- Can you tell me what it's been like for you trying these activities/changes whilst in (partial) lockdown because of coronavirus/COVID-19*?
    - Explore the context – have they been self-isolating? Shielding?
    - What have your infections been like during this time? (More/less?)
    - What aspects of lockdown have made it easier to try these activities/manage your infections?
    - What aspects of lockdown have made it harder to try these activities/manage your infections?
    - Can you tell me about any information or advice that was difficult for you to follow during lockdown?

Questions if they have not managed to try the spray:

- Can you tell about what you thought about the idea of using a spray to try to prevent infections?
- Can you tell me about anything about the spray that you liked or found easy?
- Can you tell about anything about the spray that seemed off-putting or difficult for you?
- Can you tell me about anything that worried you about using the spray?
- Can you tell me anything you feel would help you in the future with trying the spray?
- Can you tell me about whether any situations came up where you could have used the spray (e.g. first symptoms, feeling a risk of catching an infection)?
- Can you tell about how you decided whether to use the spray?
- Can you tell me about what you thought were the advantages of using the nasal spray?

For peer review only

- Can you tell me about what you thought were the disadvantages of using the nasal spray?
- Can you tell me what it's been like for you trying these activities/changes whilst in (partial) lockdown because of coronavirus/COVID-19?*
  - Explore the context – have they been self-isolating? Shielding?
  - What have your infections been like during this time? (More/less?)
  - What aspects of lockdown have made it easier to try these activities/manage your infections?
  - What aspects of lockdown have made it harder to try these activities/manage your infections?
  - Can you tell me about any information or advice that was difficult for you to follow during lockdown?

Website questions:

- What did you think of website that gave you information and advice about using the nasal spray?
- Can you tell me about anything that you liked about the website?
- Can you tell me about anything that you disliked?
- Can you tell me about anything that you would change in the website?
- Can you tell me about anything that you thought was particularly relevant to you?
- Can you tell me about anything that you thought was not particularly relevant to you?
- Can you tell me about any information or advice that didn't make sense?
- How do you think that could be changed?
- Could you tell me about anything that you thought didn't work properly?
- Can you tell me about whether you went onto the website more than once? (explore why they returned/whether they found what they needed).
- Since looking at the website, how do you feel about infections now?

Spray Instructions:

- On the website, it mentions 3 situations where you should use the spray. What did you think about these instructions?
- On the website, it mentions how often to use the spray in each of these 3 situations. What did you think about these instructions? (prompting around the instructions).
- On the website, there is a video about how to use the spray. What did you think about this?
- Can you tell me what you thought about the paper booklet about the spray? (repeat questions above as necessary- liked, disliked etc).

Open-ended Questions about personal experiences of RTIs:

1. Can you tell me all about your experience of these sorts of infections [repeat list of RTIs if necessary: colds, flu, coughs, chest infections, bronchitis, ear infections, sinusitis, sore throats, throat infections and tonsillitis].

For peer review only

- Prompts:
  - Can you tell me about the types of illnesses you tend to get?
  - Can you tell me about when you tend to get these illnesses?
  - What's it like for you when you have them?
  - How often do you get them?
  - How long do they last?

2. Can you tell me about why you think you get these sorts of illnesses?
   - Prompts:
     - Can you tell me about what you think the causes of these illness?
     - Any other reasons why you think you get them?
3. Can you tell me about things you do to try and stop getting these illnesses?
   - Prompts:
     - What made you decide to use these things? Why is it important for you to x/y/z? (e.g. eat healthy, exercise, get the flu jab)
     - How helpful do you find these things?
     - Why do you think they work?
4. When you have these sorts of illnesses is there anything you do to try and make it go away quicker?
   - Prompts
     - Any things you take, or things you do, or avoid doing?
     - What made you decide to use these things? Why is it important for you to x/y/z?
     - How helpful do you find these things?
     - Why do you think they work?

[*coronavirus question and probing was not in the original interview schedule and was added in for later interviews]

For peer review only

Supplementary material 5: COREQ checklist

Tong et al 2007, 32 item check list: https://academic.oup.com/intqhc/article/19/6/349/1791966

		Item	Guide questions/description	Manuscript section where information can be found
**Domain 1: Research team and reflexivity**				
Personal Characteristics	1	Interviewer/facilitator	Which author/s conducted the interview or focus group?	Method- data collection – Phase B: Post-intervention interviews (page 8)
	2	Credentials	What was their occupation at the time of the study?	Method- data collection – Phase B: Post-intervention interviews (page 8)
	3	Occupation	What was their occupation at the time of the study?	Method- data collection – Phase B: Post-intervention interviews (page 8)
	4	Gender	Was the researcher male or female?	Method- data collection – Phase B: Post-intervention interviews (page 8)
	5	Experience and training	What experience or training did the researcher have?	Method- data collection – Phase B: Post-intervention interviews (page 8)
Relationship with participants	6	Relationship established	Was a relationship established prior to study commencement?	Method- recruitment – Study 2 (page 7)
	7	Participant knowledge of the interviewer	What did the participants know about the researcher? e.g. *personal goals, reasons for doing the research*	Method- recruitment – Study 2 (page 7)
	8	Interviewer characteristics	What characteristics were reported about the interviewer/facilitator? e.g. *Bias, assumptions, reasons and interests in the research topic*	Method- data collection (page 8)<br>Funding statement (page 25)
**Domain 2: study design**				
Theoretical framework	9	Methodological orientation and Theory	What methodological orientation was stated to underpin the study? *e.g. grounded theory, discourse analysis, ethnography, phenomenology, content analysis*	Method (page 6, page 7, page 9)
Participant selection	10	Sampling	How were participants selected? *e.g. purposive, convenience, consecutive, snowball*	Method- recruitment (page 7)
	11	Method of approach	How were participants approached? e.g. *face-to-face, telephone, mail, email*	Method- recruitment (page 7)
	12	Sample size	How many participants were in the study?	Method- recruitment (page 7)
	13	Non-participation	How many people refused to participate or dropped out? Reasons?	n/a

				Not reported in main manuscript for conciseness.<br><br>Our recruitment method does not allow us to know why participants did not respond to our invitation to participate.
	14.	Setting of data collection	Where was the data collected? e.g. *home, clinic, workplace*	Method- data collection (page 7, page 8)
	15.	Presence of non-participants	Was anyone else present besides the participants and researchers?	Not reported in main manuscript for conciseness.<br><br>Participants were asked to be in a quiet room with no interruptions but we do not know for sure if it was always possible as most interviews were via telephone. Field notes and interview recordings from one face-to-face interview suggest a spouse was present and commenting occasionally.
	16.	Description of sample	What are the important characteristics of the sample? *e.g. demographic data, date*	Findings – Table 2 (page 14)
Data collection	17.	Interview guide	Were questions, prompts, guides provided by the authors? Was it pilot tested?	Method- PPI section (page 9), data collection – post-intervention interviews (page 8); Supplementary Material 4
	18.	Repeat interviews	Were repeat interviews carried out? If yes, how many?	Method- data collection – post-intervention interviews (page 8), Table 2 (page 14).
	19.	Audio/visual recording	Did the research use audio or visual recording to collect the data?	Method- data collection – post-intervention interviews (page 8)
	20	Field notes	Were field notes made during and/or after the interview or focus group?	Method- data collection – post-intervention interviews (page 8)
	21.	Duration	What was the duration of the interviews or focus group?	Method- data collection – post-intervention interviews (page 8)
	22.	Data saturation	Was data saturation discussed?	Not reported in main manuscript for conciseness.<br><br>The authors are very cautious about claims of data saturation.<br>Data saturation for the current analysis was not aimed for but may have been achieved or approached.<br><br>Recruitment ceased when iterative intervention development was concluded i.e. the research team were satisfied that the interventions were as engaging

For peer review only

				as possible and a range of different viewpoints from patients with different clinical and demographic characteristics had been heard and used.
	23.	Transcripts returned	Were transcripts returned to participants for comment and/or correction?	n/a<br><br>(member checks with participants were not conducted, professional transcribers transcribed the interviews and researchers checked for accuracy)
**Domain 3: analysis and findings**				
Data analysis	24.	Number of data coders	How many data coders coded the data?	Method- data collection – analysis (page 7, page 9)
	25.	Description of the coding tree	Did authors provide a description of the coding tree?	Method- data collection – analysis (page 7, page 9) A coding tree was not used. We present a description of our process of inductive thematic analysis.
	26.	Derivation of themes	Were themes identified in advance or derived from the data?	Method- data collection – analysis (page 7, page 9)
	27.	Software	What software, if applicable, was used to manage the data?	Method- data collection – analysis (page 7, page 9)
	28.	Participant checking	Did participants provide feedback on the findings?	n/a<br><br>(member checks were not conducted)
Reporting	29.	Quotations presented	Were participant quotations presented to illustrate the themes / findings? Was each quotation identified? e.*g. participant number*	Findings Table 1 (page 12) and throughout the findings section.
	30.	Data and findings consistent	Was there consistency between the data presented and the findings?	Findings (page 10)
	31.	Clarity of major themes	Were major themes clearly presented in the findings?	Findings (page 10)
	32.	Clarity of minor themes	Is there a description of diverse cases or discussion of minor themes?	n/a

For peer review only

Supplementary material 6: Themes and how they were used for intervention development

**Study findings**<br><br>**Theme title (and study where it was identified)**	**Summary of theme/finding**	**"Immune Defence" nasal spray Intervention component/content**<br>(NB previous research, theory, stakeholder opinions also fed into these decisions alongside Study 1 and 2 findings)
Motivation to avoid infections (study 1)<br><br>Excitement and optimism about a novel prevention method (study 2)	High motivation to avoid RTI (for a range of health, work, social reasons)<br><br>Explanation of spray mechanism and ways of using generated interest, hope, willingness to try.	These findings, in conjunction with our target group characteristics (recurrent RTIs/vulnerable to RTIs) meant we decided to not include significant content to convince of the necessity of avoiding infections. We kept content about the impact of RTIs and necessity of avoiding them brief and used this section predominantly to show empathy, establish a connection with users and help convince them that the intervention was relevant to them.
Inevitability (study 1)	Beliefs/experiences that RTIs are inevitable and can't be prevented or course altered once they have begun	Acknowledge current feeling and experiences of lack of control/inevitability but then build a convincing rationale for how the spray provides a chance to prevent/avoid RTIS. Describe a novel, interesting, plausible mechanism that people can understand as working in a different way to current/past prevention strategies they may have tried and experienced as ineffective.
Alternative approaches to infection prevention (study 1)	Belief that other approaches are (more) helpful for preventing RTIs	Do not attempt to persuade that any specific existing behaviours/habits/prevention methods are unhelpful/unnecessary, but refer to overall experience of wanting to gain more control and protection from infections.<br><br>Position the spray as an extra protection measure (along with novelty message and convincing rationale about how it works).
Recommendations from others (study 1)	Other people's recommendations are important	Provide a strong message of recommendation. This is given authority by NHS, University involvement and 'meet the team' of experts page and reference to scientific research.

For peer review only

Protection from risky situations (study 1)<br><br>Considering use in risky situations (study 2)	Interest in using spray to protect oneself from RTIs in situations perceived to be high risk<br><br>Considerable interest in using spray to protect oneself from RTIs n situations perceived to be high risk, especially during COVID-19 pandemic.<br><br>Some ability to correctly identify high risk situations but also some difficulty/uncertainty, especially in the context of the COVID-19 pandemic and its restrictions/mitigations.	Provide a positive message about being able to take steps to protect yourself.<br><br>Help users identify high risk situations to use the spray in. Provide examples of when to use the spray (using some examples that study participants spontaneously came up with – e.g. Public transport, aeroplanes, childcare/grandchildren situations).<br><br>Use follow-up intervention emails to revisit/remind about the types of situations and the ability of the spray to work in addition to existing mitigations. We had to edit intervention emails in real time to ensure situations and examples are well aligned with pandemic risk levels, lockdowns, restrictions.<br><br>Description of the spray as an extra layer of protection in addition to existing mitigations (e.g. Face coverings, handwashing).
Ease or difficulty (study 1)<br><br>Familiarity, confidence and information needs (study 2)	Participants vary in how easy or difficult they find using the spray. Overall, it is easy but some aspects of it require attention for best results.<br><br>Depending, in part, on past experiences of nasal sprays people may be under or over-confident in using the spray. This could lead to either anxiety about using the spray or failing to follow the instructions.	Persuasion (text) and demonstration (video) that the spray use is easy, quick and convenient.<br><br>Acknowledgment that it may take more than one use to perfect the technique (e.g. "After a few tries you will work out what feels comfortable for you", "you'll soon get the hang of it").<br><br>Clear instructions to ensure that it is experienced as easy and identified uncertainties and concerns are eliminated.<br><br>Short instructions, supplemented by optional more detail (website: drop down sections; booklet=short infographic style instructions plus reference to website for further information)<br><br>Persuasive text to stop people skipping important information by highlighting why it is useful/new (e.g. "check out this video to see how to use your nasal spray. This video is worth watching even if you have used nasal sprays before. The technique for **this spray**

For peer review only

		might be a bit different. Using the spray correctly give you the best chance of fighting infections!")<br><br>Inclusion of a suggestion to try the spray out when it arrives to boost confidence prior to time it is needed.
Experiencing side effects (study 1)<br><br>Reactions to possible/actual side effects (study 2)	Side effects of the spray are common, milder ones are tolerated if benefits are expected/experienced. Strong side effects can prevent further use.<br><br>Reassuring information about side effects is valued. People describe being willing to try the spray despite minor side effects. Severe side effects seem likely to influence discontinuation.	Explanation that side effects are minor<br><br>Framing of sensations in nose and throat (e.g. tingling, noticeable taste) as normal and a positive sign the spray is working/reaching the right place rather than a side effect.<br><br>Comparisons of mild side effects with more severe and prolonged symptoms of 'full blown infection'.<br><br>Instruction and demonstration on how to avoid the more severe side effects (spray technique).<br><br>Instructions on how to cope with side effects (e.g. position to adopt for nose bleeds, use of saline solution for dry/irritated nose, eating/drinking to eliminate unpleasant taste).
Identifying early signs of infection (Study 2)	Participants often but not always have awareness of first signs of infection and confidence in being able to use the spray in response	Give sufficient information about which first signs are relevant by listing main signs that people recognise as relevant to RTIs (feeling in throat, malaise) but also allowing for idiosyncratic first signs.<br><br>Acknowledge the difficulty distinguishing some symptoms (e.g. Runny nose, sneezing - hayfever & RTI overlap).<br><br>Explain and reassure that it would be advisable and safe to use on a symptom that turned out not to be an RTI symptom.<br><br>Given that we know people may miss first signs, refer to failure to act quickly enough as a possible explanation for situations where

For peer review only

		the spray does not appear to have helped (to try to prevent users from concluding that the spray is ineffective).
Expectations and experiences of success and failure (study 1)<br><br>Excitement and Optimism about a novel prevention method (study 2)	Users experience combinations of success and failure with the spray which then influence the continuation of use.<br><br>Idea of spray elicits interest, hope, willingness to try. For some this is very pronounced, for others it is more muted or sceptical.	Promote excitement, interest, positivity and expectations of success (via convincing explanation of how it works)<br><br>Provide a rationale for how spray use might lead to partial success (not avoiding infections but having shorter and less severe).<br><br>Provide a rationale for why it might not always work (using too late)<br><br>Encourage persistence if it does not appear to work, including attributing failure to using it too late and explaining that it may nonetheless have reduced infection severity and duration.<br><br>Encourage formation of the spray as a low risk / safe / easy intervention suitable for regular use in order to help them to conclude that potential for benefit outweighs concerns, even if they do not experience clear cut evidence of success (i.e. It is like a hand gel, not a medicine, and with no serious side effects)
Excitement and optimism about a novel prevention method (study 2)<br><br>Consequences of feeling protected (study 2)	Idea of spray elicits interest, hope, willingness to try. For some this is very pronounced, for others it is more muted or sceptical.<br><br>Feeling protected may make people feel safer, more confident and more able to participate in valued activities. It could also make people take more risks.	Boost positive expectancies of the spray's psychosocial effects including how the spray can make you feel more confident and in control and that it feels good to feel more protected [emails]<br><br>Alongside positive expectations of spray efficacy, promote continued adherence to COVID-19 regulations/guidance/mitigations; positioning the spray as an additional, not a replacement behaviour.<br><br>Do not refer to or recommend against any current infection control behaviours (e.g. Keeping distance from ill people, good respiratory hygiene, good diet, being physically fit)
Concern about medicines (Study 2)	People see nasal sprays as a medicine, eliciting medication-related	Explicitly position the spray as not a medicine, whilst maintaining expectations that it will be a powerful and effective product.

For peer review only

	concerns such as overuse, allergies, contraindications	Compare it instead to hand gel (noting the similar mechanisms- cleaning away virus before it can cause illness).<br><br>Provide reassuring information about how often it is safe to use it and how it can be used with any prescription and over-the-counter medications.<br><br>Position spray as a means of avoiding using medications such as antibiotics, over-the-counter cold relief.<br><br>NB- we expect concerns related to medicines to persist to some degree in some participants despite our 'not a medicine' message. The spray might, to a layperson, feel like a medicine in terms of its mode of administration and anticipated efficacy. Our content nonetheless promotes beliefs about it being a simple, safe and effective intervention.
Disgust and hygiene (Study 2)	Noses and nasal sprays can be considered disgusting and/or messy and unhygienic	Reassurance that the spray procedure is not wet, messy or unpleasant.<br><br>This required a change (between study 2a and study 2b) from our original description of the spray being not like a medicine but like washing hands with soap and water. We adopted a neater/cleaner explanation (like hand gel). The public were becoming very familiar with hand gel as an important infection control product at this point in the COVID-19 pandemic.<br><br>Emphasise easiness of using the spray.<br><br>Instructions on how to use hygienically.

For peer review only
